# Interventions promoting uptake of water, sanitation and hygiene (WASH) technologies in low‐ and middle‐income countries: An evidence and gap map of effectiveness studies

**DOI:** 10.1002/cl2.1194

**Published:** 2021-10-08

**Authors:** Hannah Chirgwin, Sandy Cairncross, Dua Zehra, Hugh Sharma Waddington

**Affiliations:** ^1^ International Initiative for Impact Evaluation (3ie) London International Development Centre London UK; ^2^ London School of Hygiene and Tropical Medicine London UK; ^3^ University College London London UK; ^4^ London School of Hygiene and Tropical Medicine and International Initiative for Impact Evaluation (3ie) London International Development Centre London UK

## Abstract

**Background:**

Lack of access to and use of water, sanitation and hygiene (WASH) cause 1.6 million deaths every year, of which 1.2 million are due to gastrointestinal illnesses like diarrhoea and acute respiratory infections like pneumonia. Poor WASH access and use also diminish nutrition and educational attainment, and cause danger and stress for vulnerable populations, especially for women and girls. The hardest hit regions are sub‐Saharan Africa and South Asia. Sustainable Development Goal (SDG) 6 calls for the end of open defecation, and universal access to safely managed water and sanitation facilities, and basic hand hygiene, by 2030. WASH access and use also underpin progress in other areas such as SDG1 poverty targets, SDG3 health and SDG4 education targets. Meeting the SDG equity agenda to “leave none behind” will require WASH providers prioritise the hardest to reach including those living remotely and people who are disadvantaged.

**Objectives:**

Decision makers need access to high‐quality evidence on what works in WASH promotion in different contexts, and for different groups of people, to reach the most disadvantaged populations and thereby achieve universal targets. The WASH evidence map is envisioned as a tool for commissioners and researchers to identify existing studies to fill synthesis gaps, as well as helping to prioritise new studies where there are gaps in knowledge. It also supports policymakers and practitioners to navigate the evidence base, including presenting critically appraised findings from existing systematic reviews.

**Methods:**

This evidence map presents impact evaluations and systematic reviews from the WASH sector, organised according to the types of intervention mechanisms, WASH technologies promoted, and outcomes measured. It is based on a framework of intervention mechanisms (e.g., behaviour change triggering or microloans) and outcomes along the causal pathway, specifically behavioural outcomes (e.g., handwashing and food hygiene practices), ill‐health outcomes (e.g., diarrhoeal morbidity and mortality), nutrition and socioeconomic outcomes (e.g., school absenteeism and household income). The map also provides filters to examine the evidence for a particular WASH technology (e.g., latrines), place of use (e.g., home, school or health facility), location (e.g., global region, country, rural and urban) and group (e.g., people living with disability). Systematic searches for published and unpublished literature and trial registries were conducted of studies in low‐ and middle‐income countries (LMICs). Searches were conducted in March 2018, and searches for completed trials were done in May 2020. Coding of information for the map was done by two authors working independently. Impact evaluations were critically appraised according to methods of conduct and reporting. Systematic reviews were critically appraised using a new approach to assess theory‐based, mixed‐methods evidence synthesis.

**Results:**

There has been an enormous growth in impact evaluations and systematic reviews of WASH interventions since the International Year of Sanitation, 2008. There are now at least 367 completed or ongoing rigorous impact evaluations in LMICs, nearly three‐quarters of which have been conducted since 2008, plus 43 systematic reviews. Studies have been done in 83 LMICs, with a high concentration in Bangladesh, India, and Kenya. WASH sector programming has increasingly shifted in focus from what technology to supply (e.g., a handwashing station or child's potty), to the best way in which to do so to promote demand. Research also covers a broader set of intervention mechanisms. For example, there has been increased interest in behaviour change communication using psychosocial “triggering”, such as social marketing and community‐led total sanitation. These studies report primarily on behavioural outcomes. With the advent of large‐scale funding, in particular by the Bill & Melinda Gates Foundation, there has been a substantial increase in the number of studies on sanitation technologies, particularly latrines. Sustaining behaviour is fundamental for sustaining health and other quality of life improvements. However, few studies have been done of intervention mechanisms for, or measuring outcomes on sustained adoption of latrines to stop open defaecation. There has also been some increase in the number of studies looking at outcomes and interventions that disproportionately affect women and girls, who quite literally carry most of the burden of poor water and sanitation access. However, most studies do not report sex disaggregated outcomes, let alone integrate gender analysis into their framework. Other vulnerable populations are even less addressed; no studies eligible for inclusion in the map were done of interventions targeting, or reporting on outcomes for, people living with disabilities. We were only able to find a single controlled evaluation of WASH interventions in a health care facility, in spite of the importance of WASH in health facilities in global policy debates. The quality of impact evaluations has improved, such as the use of controlled designs as standard, attention to addressing reporting biases, and adequate cluster sample size. However, there remain important concerns about quality of reporting. The quality and usefulness of systematic reviews for policy is also improving, which draw clearer distinctions between intervention mechanisms and synthesise the evidence on outcomes along the causal pathway. Adopting mixed‐methods approaches also provides information for programmes on barriers and enablers affecting implementation.

**Conclusion:**

Ensuring everyone has access to appropriate water, sanitation, and hygiene facilities is one of the most fundamental of challenges for poverty elimination. Researchers and funders need to consider carefully where there is the need for new primary evidence, and new syntheses of that evidence. This study suggests the following priority areas:

- Impact evaluations incorporating understudied outcomes, such as sustainability and slippage, of WASH provision in understudied places of use, such as health care facilities, and of interventions targeting, or presenting disaggregated data for, vulnerable populations, particularly over the life‐course and for people living with a disability;
- Improved reporting in impact evaluations, including presentation of participant flow diagrams; and
- Synthesis studies and updates in areas with sufficient existing and planned impact evaluations, such as for diarrhoea mortality, ARIs, WASH in schools and decentralisation. These studies will preferably be conducted as mixed‐methods systematic reviews that are able to answer questions about programme targeting, implementation, effectiveness and cost‐effectiveness, and compare alternative intervention mechanisms to achieve and sustain outcomes in particular contexts, preferably using network meta‐analysis.

## 1 PLAIN LANGUAGE SUMMARY

### 1.1 There is a substantial body of evidence on the effectiveness of WASH interventions—Investment in reviews is needed

Lack of access to and use of water, sanitation and hygiene (WASH) causes 1.6 million deaths every year, of which 1.2 million due to gastrointestinal illnesses like diarrhoea and acute respiratory infections like pneumonia. Poor WASH also diminishes nutrition and educational attainment, and causes danger and stress for vulnerable populations, especially for women and girls.

Sustainable Development Goal (SDG) 6 calls for the end of open defecation, and universal access to safely managed water and sanitation facilities, and basic hand hygiene, by 2030. WASH access and use also underpin progress in other areas such as SDG1 poverty targets, SDG3 health and SDG4 education targets.

### 1.2 What is this evidence and gap map about?

There has been substantial growth in the evidence base for WASH interventions in recent years, with increased attention to behaviour change.

This evidence and gap map (EGM) is based on a framework of intervention mechanisms and outcomes along the causal pathway, specifically behavioural outcomes, ill‐health outcomes, nutrition, and socioeconomic outcomes.

**What is the aim of this evidence and gap map (EGM)?**

The aim of this EGM is to show all the available evidence from systematic reviews and impact evaluations of what works in water, sanitation and hygiene (WASH) promotion in low‐ and middle‐income countries.

### 1.3 What studies are included?

The map includes 367 rigorous impact evaluations of WASH interventions in low‐ and middle‐income countries (LMICs), nearly three‐quarters of which have been conducted since 2008, plus 43 systematic reviews.

WASH impact evaluations have been done in 83 LMICs, covering over 5 million participants. There is a high concentration in Bangladesh, Kenya and India, each having over 50 studies.

### 1.4 What are the included studies about?

Over the past 15 years, the focus of impact evaluation research has shifted from WASH technology provision to promotional interventions. There has been an increase in studies of behaviour change communication, particularly for hand hygiene using social marketing and community‐led total sanitation.

Carer‐reported diarrhoeal illness among children remains the standard health impact measure used, and is by far the most commonly reported outcome. The map includes 186 studies measuring diarrhoea morbidity.

Analysis of mortality is less common: just 27 studies have examined impacts on child survival in LMICs, despite mortality being the main component of the global burden of disease due to inadequate WASH. Only 35 studies measure acute respiratory infection.

The most commonly reported behaviours are handwashing, water treatment and handling, and latrine use. Nearly 50 studies specifically collected data on handwashing before food preparation, and over 20 report other hygiene behaviours. There are also five studies of menstrual hygiene management.

The opportunity costs of women and children's time spent collecting water, or illness in childhood due to inadequate access to water, sanitation and hygiene, include education and economic impacts. Twenty‐three studies measured various aspects of time savings and alternative uses of time due to water supply improvements. However, only six studies measured labour market outcomes.

### 1.5 What do the findings of the map mean?

The map shows there is a sizeable evidence base for WASH interventions. Researchers and funders should consider carefully where there is the need for new primary evidence, and new syntheses of that evidence. This study suggests the following priority areas:

- Impact evaluations of interventions targeting understudied outcomes, such as sustainability and slippage, in understudied places of use, such as health care facilities, and among populations that are disadvantaged;
- Improved reporting in impact evaluations including full reporting of participant flows, as per CONSORT guidance, and clearer reporting about intervention and control conditions, including the availability of water supply (accessibility and reliability);
- Natural experiments that can measure the impacts of WASH on mortality rigorously, ethically and with sufficient statistical power;
- New and updated systematic reviews in areas with sufficient impact evaluations, such as for diarrhoea mortality, acute respiratory infections, time use, WASH in schools, and decentralisation;
- More high confidence systematic reviews, which systematically incorporate unpublished studies, and use mixed methods to analyse intervention processes and outcomes along the causal pathway.

### 1.6 How up‐to‐date is this EGM?

The authors searched for studies published up to May 2020.

## 2 BACKGROUND

### 2.1 Introduction

Water, sanitation and hygiene (WASH) are human rights that underpin the most basic of needs. Most fundamentally, WASH affects the likelihood of survival beyond early childhood, and also determines whether basic needs for human life—such as nutrition, excretion and safety—as well as higher order needs—like dignity, productivity, and happiness—are met. Yet, according to the WHO and UNICEF Joint Monitoring Programme (JMP), 2 billion people do not have safe, readily available water at home, and 4.5 billion lack access to safely managed sanitation services (WHO/UNICEF, 2019a). Worldwide, nearly a billion people practice open defecation. Rural, poor and vulnerable households have particularly limited access to adequate facilities and inequities are often regionally focused. People in sub‐Saharan Africa have the worst rates of access to improved drinking water sources and hygiene, where 400 million people use surface water or only have access to improved water sources that take more than 30 min round‐trip to collect. Of the 1.4 billion people who defecate in the open or use unimproved or shared sanitation facilities, 505 million are living in South Asia (of which 375 million are in India) and 546 million are in sub‐Saharan Africa.

**Figure 1:**
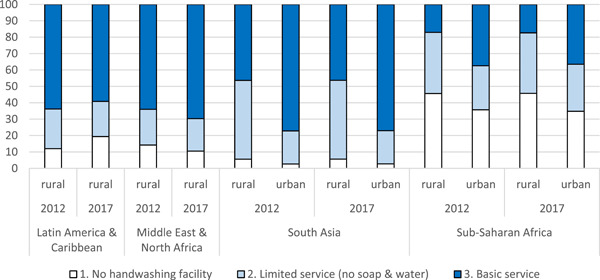
Household hygiene access (% of population using service). Data not available for EAP (rural and urban), and urban LAC and MENA. *Source*: data collected from https://washdata.org/. EAP, East Asia and the Pacific; LAC, Latin America and the Caribbean; MENA, Middle East and North Africa

Available data on access to hygiene facilities (Figure 1), indicates the biggest share of people without access to even basic hygiene facilities, defined as fixed or mobile handwashing facilities with soap and water, is in sub‐Saharan Africa and South Asia, where no significant improvements in coverage were made in 2012–2017. Over 80% of rural Africans, 530 million people, do not use a handwashing facility or use limited services without soap and water. Over half of rural South Asians, 640 million, also have no or limited handwashing services. No data are available for handwashing in East Asia and the Pacific. Furthermore, those lacking access to basic handwashing facilities in Latin America and the Caribbean has increased, from 46 to 52 million people.

**Figure 2:**
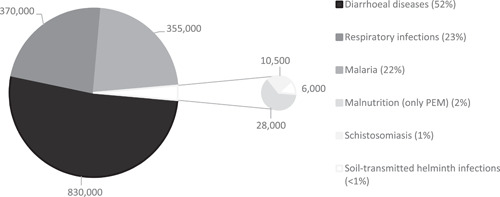
Annual global mortality due to inadequate WASH. PEM protein energy management. *Source*: data from Prüss‐Ustün et al. ( 2019). PEM, protein energy management; WASH, water, sanitation and hygiene

The consequences are far‐reaching. Limited, or no, access to safe facilities for eliminating human waste, obtaining sufficient water for drinking or practicing hygienic washing and food preparation practices exposes individuals to higher levels of deadly infection. Inadequate WASH can contribute to the outbreak and chronic presence of preventable infections like acute respiratory infections (ARIs; Aiello et al., 2008; Rabie & Curtis, 2006) such as pneumonia and, recently, COVID‐19,^1^ diarrhoeal disease (Cairncross et al., 2010; De Buck et al., 2017; Waddington et al., 2009; Wolf et al., 2018), parasitic worm infections (e.g., Ascaris, Trichuris and hookworm infections) (Freeman et al., 2017; Ziegelbauer et al., 2012), water‐washed skin and eye infections like trachoma (Freeman et al., 2017; Rabiu et al., 2012). It may also cause tropical enteropathy, a subclinical disorder where the lining of the gut wall is damaged by repeated bouts of enteric infection until it is unable to absorb nutrients adequately (Humphrey, 2009; Shiffman et al., 1978). Chronic high infection rates are among the leading causes of undernutrition and death in children in developing countries (Cairncross et al., 2014). According to recent Global Burden of Disease estimates, inadequate WASH is associated with 1.6 million deaths per year, due to diarrhoea, ARI, malnutrition due to protein energy management and, as a result of water mismanagement, malaria (Figure 2). Diarrhoea alone kills 850,000 people every year, 300,000 of which are children aged under 5 (Prüss‐Ustün et al., 2019).

Beyond the potentially life‐threatening consequences of ARIs like influenza and enteric infections like diarrhoea, poor access and use of WASH may also affect social and economic outcomes, both directly and through a ripple effect. This may include diminished educational attainment (Hennegan et al., 2017), with implications for life‐time earnings (Hutton et al., 2006; Turley et al., 2013) at the end of the causal pathway. Inadequate access disproportionately affects groups who are disadvantaged, but women and girls are particularly affected by the danger and stress of having limited access to WASH facilities. They often carry the majority of the burden associated with collecting water—including time, calories spent, musculoskeletal injuries, road casualties, and risks of assault and attack by “pests and pervert” (Campbell et al., 2014). For example, they can be placed in high‐risk situations when using unsafe places to defecate at nighttime (Cairncross & Valdmanis, 2006; Cairncross et al., 2010; Sahoo et al., 2015; Sorenson et al., 2011). Women and adolescent girls also experience hardships where inadequate WASH facilities constrain menstrual hygiene management causing urinary tract infections (Torondel et al., 2018) and absence from school and work (Hennegen et al., 2016; Sumpter & Torondel, 2013).

While WASH programmes, policy, and research has typically focused on people's needs at home, there is an increasing understanding of the importance of WASH infrastructure in institutions that provide public services, especially schools and health facilities. WHO/UNICEF (2019b) estimated that 26% of health care facilities globally do not have access to an improved water source, 21% lack sanitation services, and 16% do not have water and soap for handwashing. A lack of WASH infrastructure can increase care‐related infections, birth complications, and water‐related disease outbreaks, as well as discouraging the uptake of services (Benova et al., 2014; WHO, 2015). WHO/UNICEF (2018) also established a global baseline for drinking water, sanitation and hygiene in schools. The recent monitoring report indicated one‐third of schools lack adequate access to safe water and sanitation, and 20% had no access to sanitation at all, and only 57% have basic handwashing facilities, which may affect school attendance, learning, and gender equality (WHO/UNICEF, 2020).

There are also concerns about sustainability in access. The United Nations (2019) predicts the global population to reach 8.5 billion by 2030 and 9.7 billion by 2050, increasing the demand and competition for basic services and resources including clean water. Additionally, greater climate variability associated with global climate change are expected to trigger extreme weather events such as cyclones, flooding and drought. More frequent and more severe disasters cause loss and damage of supplies, making sustained access to WASH all the more challenging (Global Water Partnership and UNICEF, 2014). Taking the case of slums in sub‐Saharan Africa, for example, recurrent floods and insufficient stormwater drainage has been known not only to contaminate clean water supplies—leading to outbreaks of cholera (WaterAid, 2017)—but also to trigger disease outbreaks like malaria from stagnant water (Zehra et al., 2019). In Sub‐Saharan Africa alone, 59% of the urban population currently resides in slums, and it is estimated that 1.2 billion residents will be slum dwellers by 2050 (UN Habitat, 2016). Coupled with the increased climate hazards, the growth in both global and urban populations points to a major challenge for ensuring populations have adequate and safe WASH facilitation in years to come.

In 2015, more than 150 world leaders adopted the new 2030 Agenda for Sustainable Development, which set new goals for 2030 that build upon, and go even further, than the Millennium Development Goals. Sustainable Development Goal (SDG) 6 aims to “ensure the availability and sustainable management of water and sanitation for all” by 2030 (UN Water, 2018). It includes goals to:

- End open defecation by ensuring that everyone has access to at least a basic toilet and safe waste disposal system.
- Provide universal access to safe, and affordable, drinking water.
- Provide universal access to basic hygiene facilities.
- Pay attention to the specific needs of women and vulnerable populations.
- Basic drinking water, single‐sex basic sanitation and basic handwashing facilities in schools and Monitor WASH in schools and health facilities.
- Expand international cooperation and strengthen the capacity of local and national bodies to manage their water and sanitation systems.

WASH also underpins progress in a number of other areas such as SDG1 poverty targets, SDG3 health targets and SDG4 education targets (Table 1). A number of strategic global initiatives were established to improve WASH agency coordination, to avoid duplication of effort and promote synergies in activities, and the monitoring and evaluation of activities and outcomes, to promote evidence‐based decision making. Two major initiatives to coordinate monitoring progress are the WHO/UNICEF JMP, which provides data and an annual report on access to and use of water and sanitation since 1990, and, the WHO and UN Water's Global Analysis and Assessment of Sanitation and Drinking‐Water, which monitors global resource flows and policy commitments since 2008.^2^

**Table 1:** SDGs relevant to water, sanitation and hygiene for consumption in households and public facilities

SDG	Target definition	Indicator
6.1	To provide safe and affordable drinking water for all by 2030	Proportion of population using safely managed drinking water that is from an improved drinking water source, located on premises, available when needed and free from contamination
6.2	To provide adequate and equitable sanitation for all and end open defaecation by 2030, ensuring that everyone has access to at least a basic toilet and safe waste disposal system, paying special attention to the needs of women, girls and vulnerable people	Proportion of population using safely managed sanitation services, defined as an improved facility where excreta is treated and disposed of in situ or off‐site
6.2	Provide universal access to a basic hand washing facility with soap and water by 2030	Proportion of population using a hand‐washing facility with soap and water
6.3	Improve water quality by, among others, halving the proportion of untreated wastewater and substantially increasing recycling and safe reuse globally by 2030	Proportion of wastewater safely treated and proportion of water bodies with good ambient water quality
6.4	Substantially increase water‐use efficiency and address water scarcity by 2030	Freshwater withdrawal as a proportion of available freshwater resources
6.A	Expand international cooperation and capacity‐building support to developing countries in water‐ and sanitation‐related activities and programmes by 2030, including water harvesting, desalination, water efficiency, wastewater treatment, recycling and reuse technologies	Amount of water‐ and sanitation‐related official development assistance that is part of a government‐coordinated spending plan
6.B	Support and strengthen participation of local communities in improving water and sanitation management	Proportion of local administrative units with established and operational policies and procedures for participation of local communities in water and sanitation management
1.4	To ensure all men and women, in particular the poor and vulnerable, have access to basic services by 2030	Proportion of people living in households with access to basic services (including water, sanitation and hygiene)
3.3	End epidemics of AIDS, tuberculosis, malaria and NTDs and combat hepatitis, waterborne diseases and other communicable diseases by 2030	Tuberculosis, malaria and hepatitis B incidence and number of people requiring interventions against NTDs
3.9	To reduce substantially deaths and illnesses from hazardous chemicals and water pollution and contamination by 2030	Mortality rate attributed to unsafe water, unsafe sanitation and lack of hygiene
4.A	Build and upgrade education facilities that are child, disability and gender sensitive and provide safe, nonviolent, inclusive and effective learning environments for all	Proportion of schools with, amongst others, basic drinking water, single‐sex basic sanitation and basic handwashing facilities (as per the WASH indicator definitions)

Abbreviations: NTD, neglected tropical disease; SDG, Sustainable Development Goal.

*Source*: United Nations (undated).

**Figure 3:**
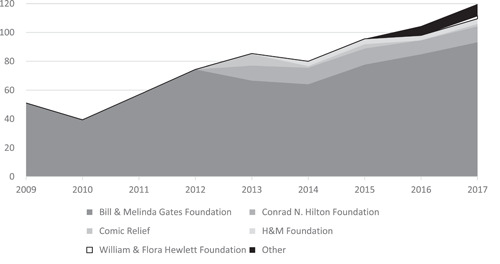
Private donor disbursements to water and sanitation. 2017 US$ millions. *Source*: Creditor Reporting System https://stats.oecd.org/

Meeting the SDG equity agenda to “leave no‐one behind” will require decision makers to prioritise the hardest to reach including people living remotely and those who are disadvantaged (e.g., children, the elderly, displaced populations, and people living with disability). These decision makers need access to high quality evidence on the effects of WASH promotion approaches in different contexts, for different groups of people. There is increasing recognition of the role of rigorous evidence in facilitating efficiency improvements to meet development targets (e.g., Waddington et al., 2018). Major contributions to building the rigorous evidence base have been made since the International Year of Sanitation (2008). Rivalling traditional Development Assistance Committee donors in contributing resources for WASH programming and research are private philanthopists, of which the biggest by far is the Gates Foundation (Bill and Melinda Gates Foundation). The Gates Foundation provided US$93 million (2016 prices) to the sector in 2017 (Figure 3).

Global policy decisions should draw on systematic evidence (e.g., systematic reviews, meta‐analyses and evidence maps) that examines the totality of evidence and not the results of single studies or chosen groups of studies. This is because single studies, while important, are only able to provide evidence on the extent to which WASH programmes can help people overcome challenges in the context in which they are implemented. For example, two recent, high‐profile randomised controlled trials (RCTs) in Bangladesh and Kenya were not able to detect statistically precise effects of combined or single water, sanitation and hygiene interventions on child linear growth (Luby et al., 2018; Null et al., 2018). However, the studies have been criticised due to inefficacy of the interventions provided (Wilson‐Jones et al., 2019) and limited generalisability to other contexts (Coffey & Spears, 2018). Single studies like impact evaluations are often not reported in readily accessible formats, providing transparent information about interventions received and unbiased information about programme effectiveness (Pickering et al., 2019). On the other hand, high quality systematic reviews critically appraise and corroborate the findings from individual studies, as well as providing a steer to decision makers about which findings are generalisable and which are more context specific (Higgins & Green, 2011; Petticrew & Roberts, 2006; Waddington & White, 2012; Waddington et al., 2012). However, many systematic reviews are not done to standards of high confidence (Lewin et al., 2009) and many done on WASH topics are not done for policy audiences, are limited to studies published in peer review journals, and/or focus on technologies rather than WASH intervention mechanisms, the latter being the currency of WASH programming bodies.

### 2.2 Existing evidence maps and systematic reviews

Evidence maps incorporating WASH interventions and outcomes are already available. For example, a systematic scoping review produced a map of evidence on potential consequences for maternal health due to inadequate WASH (Campbell et al., 2015). An initial evidence and gap map (EGM) of household and community WASH technology promotion in LMICs was produced by 3ie and the Department of Disease Control at London School of Hygiene and Tropical Medicine (Waddington et al., 2014). The map was limited to quantitative causal studies (impact evaluations) and systematic reviews. The present study is an update of that map including updates to the searches, scope, quality appraisal and stakeholder engagement (Waddington et al., 2018). Specifically, it reorients the presentation from WASH technologies to intervention mechanisms. It also incorporates behaviour change as a primary outcome, to reflect the increasing focus on behaviour change in the literature (Aunger & Curtis, 2016; Waddington et al., 2009). It includes WASH promotion for use in private (household and yard) and public domains (including communities, schools and health facilities) (Cairncross et al., 1996), to reflect the policy focus on these areas by the JMP (WHO/UNICEF, 2017).^3^ The systematic reviews have been critically appraised using an improved tool that accounts not just for quality of searching, coding and meta‐analysis, but also incorporation of theory, qualitative evidence and quantitative evidence along the causal pathway. The stakeholder engagement included major WASH providers, such as WSSCC, now called the Sanitation and Hygiene Fund, who funded the study.

### 2.3 EGM: Definition and purpose

A standard systematic review is often completed within 12–24 months (Waddington et al., 2018), but often takes longer. Reviews can take a long time to produce findings, quickly becoming outdated in such a way that they fail to answer the questions they have been commissioned for in a timely manner (Whitty, 2015). One way to speed up the process of knowledge translation from systematic searches is the evidence map. Evidence mapping is an approach to present the extent of evidence on a topic in a user‐friendly format (Saran & White, 2019). Approaches like EGMs (Snilstveit et al., 2016) present a collection of evaluations (and evaluation syntheses) on a particular topic, in the form of an intervention‐outcome matrix (usually showing interventions on the vertical axis and outcomes along the horizontal axis), which can be filtered by study categories (e.g., place of intervention, type of participant and study design).

Evidence mapping has proven incredibly popular with researchers and development organisations (Phillips et al., 2017). It is an attempt to democratise access to information on scientific studies, which are frequently collected in journal articles and technical reports that are physically or technically inaccessible to decision makers, as well as communicate that information in a participatory format. The format is participatory because the user interface enables filtering and some aspects of quality assessment to be viewed according to the user's needs. Maps are therefore sometimes envisioned as tools for policymakers (Snilstveit et al., 2016). At the very least they are useful for researchers to identify existing studies, and commissioners of research to prioritise conducting new primary and synthesis studies (Saran & White, 2019). However, evidence maps are not a substitute for systematic reviews since they are often not designed to critically appraise or extract policy‐relevant findings from primary studies. They are still a very useful way of scoping future review topics and provide a more efficient way of communicating primary research gaps than “empty reviews”.^4^

### 2.4 Objectives

There is a long history of impact evaluation and systematic review of WASH interventions and exposures in low‐ and middle‐income countries (LMICs). For example, Wagner and Lanoix (1959) and Feachem et al. (1978) published evaluations of water supply in, respectively, Brazil and Lesotho. Briscoe et al. (1986) articulated standards for health impact evaluations in WASH. WHO's *Minimum Evaluation Procedure* (1983) argued that evaluations should focus on the functioning of the facilities, and their use, which have greater diagnostic power to improve a programme than health impact evaluations. These standards informed what may be called the “first generation” of health impact evaluations in the WASH sector—that is, the application of rigorous methods like RCTs to quantify the effects of WASH service provision on disease outcomes. The use of systematic review and meta‐analysis to synthesise the findings from summative evaluations also gained prominence during this period (starting with Esrey et al., 1985). We are now over a decade into a “second generation” of WASH impact evaluation research, during which evaluators have focused on intervention mechanisms aiming to alter behaviour and measure broader behavioural outcomes.

The overarching aim of this EGM is to democratise access to information on the WASH sector studies conducted in LMICs by identifying, mapping, and describing the existing and ongoing, and the gaps in, empirical research on the effectiveness of interventions to improve the consumption of water, sanitation and hygiene at home as well as in communities, schools, and health facilities. The map includes supply‐side intervention mechanisms to promote access to water, sanitation or hygiene services (e.g., direct provision, private sector involvement, capacity building), and demand‐side intervention mechanisms promoting use of services (e.g., consumer behaviour change communication [BCC], subsidies and microloans).

It also aims to go beyond “diarrhoea reductionism” (Chambers & von Medeazza, 2014) by incorporating behaviour change (e.g., water treatment practices, open defecation and time use), indicators of ill‐health (e.g., respiratory infection, enteric infections), nutritional status, mortality, and socioeconomic outcomes (e.g., education, income and safety) as primary outcomes.^5^

The map is envisioned as a tool for policymakers, practitioners, and researchers to identify existing work that they can use, as well as help to more efficiently commission, and conduct, new studies. It addresses five objectives:

(1) To provide a conceptual framework linking WASH intervention mechanisms (incorporating “what”, “how”, “where” and “for whom”) with behavioural, health and socioeconomic outcomes.
(2) To conduct a census of existing (and planned) evidence from impact evaluations and systematic reviews of programmes aiming to promote access to, and use of, WASH services in private and public spaces in LMICs, including homes, communities, schools and health facilities.
(3) To incorporate studies (and systematic reviews of studies) using statistical methods to attribute and quantify changes in behaviour and quality of life outcomes resulting from WASH interventions, including studies using randomised assignment (RCTs), nonrandomised studies (NRS) designed prospectively and retrospectively, and natural experiments using observational data.
(4) To present critically appraised and synthesised knowledge from systematic reviews of WASH evidence to help policy decision making.
(5) To identify gaps in existing evidence where new primary studies could be undertaken and gaps where new systematic reviews could be done.

By doing the above, the EGM aims to inform policy based on systematic evidence, as well as shape the direction of future WASH impact evaluation and synthesis research.

## 3 CONCEPTUAL FRAMEWORK

### 3.1 Scope

Before the early‐2000s, the focus of WASH impact evaluation research was principally on efficacy—that is, research centred on understanding the consequences of providing a technology at zero or negligible cost. WASH technologies were grouped into four related single WASH technology categories: water supply, water treatment, sanitation and hygiene (Esrey et al., 1991).

Over the last 15 years, and particularly in the years following the International Year of Sanitation (2008) and the subsequent influx of resources from major funders, policy and research has increasingly focused on the effectiveness of demand‐side promotional approaches targeting uptake and adoption. Different approaches have been used to promote demand‐side behaviour change in the context of water and sanitation provision. For example, directive information and education communication, social marketing and subsidies have been traditionally popular means of promoting sanitation and hygiene demand. These have been criticised as inadequate methods for triggering the level of widespread behavioural, and social, change required to achieve significant improvements (e.g., Chambers, 2009; Jenkins & Sugden, 2006). Instead, approaches grounded in behavioural science have increased in popularity.

**Figure 4:**
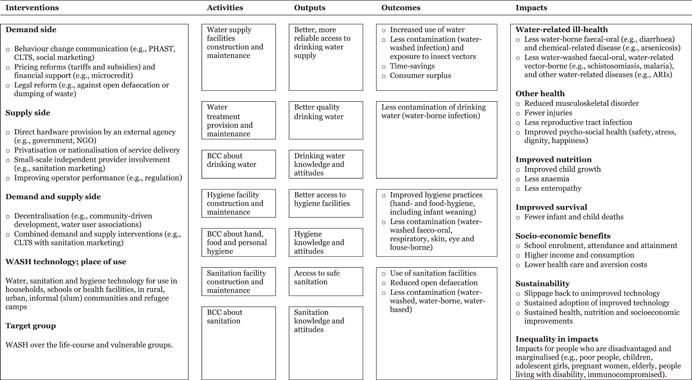
WASH sector simplified causal pathway. Outcomes are usually defined as depending on participant behaviour, whereas outputs are usually the direct consequences of WASH provision. In this schema, therefore, outcomes are behavioural, whereas outputs providing access to WASH are often technological. However, some intervention mechanisms aim to stimulate access by encouraging behaviour (e.g., construction of latrines or wells), so the distinction is not always clear cut. WASH, water, sanitation and hygiene

A conceptual framework linking WASH interventions with outcomes along the causal pathway is depicted in Figure 4. The framework was developed based on a review of the academic and policy literature, and in consultation with researchers, WASH practitioners and WASH programming organisations. Sector interventions are presented to the left of the figure: on the supply side, water and sanitation hardware provision by external agencies, improved operator performance, private sector participation and contracting out; on the demand side, behaviour change intervention mechanisms, pricing reforms and financial support; decentralisation combines demand and supply side elements. Quality of life impacts—water‐related health, other health and socioeconomic outcomes—are presented on the right. The causal pathway shows how interventions are turned into impacts, through activities (construction of new facilities, behaviour change campaigns), outputs (better access to, quality of, knowledge of, and attitudes towards WASH services and practices) and intermediate outcomes (behaviour change relating to access and use of improved WASH services).

The figure is highly simplified and excludes underlying assumptions. Links in the causal pathway between interventions and outcomes are not automatic. For example, latrine building may not reduce open defaecation or contamination in the public domain. Factors limiting use include the cleanliness and smell of the facilities, or concerns about how frequently the pit will need to be emptied. Latrine provision may also not improve health and nutrition where open defecation is practised in densely populated areas (Geruso & Spears, 2018; Kar & Chambers, 2008). Children may be afraid of going into dark places or of falling into latrine pits, creating further hazard since young children's excreta contain the most pathogens (Curtis et al., 1995).

In addition, preventive technologies tend to be adopted more slowly as benefits are difficult to observe (Rogers, 2005). This applies particularly to WASH technologies whose main benefit is to reduce diseases, the prevalence of which may typically be infrequent (or effects unobserved) outside of epidemics. In contrast, where the benefits of a technology are easily observed by those directly affected, such as poor women and children collecting water every day, adoption is likely to be rapid where access can be adequately provided. In this case, it is more likely that underinvestment in the technology would be explained by systemic undervaluation of the benefits and costs (including opportunity costs) for the affected groups, both by public authorities and household decision makers.

Sustaining impacts and achieving impacts at scale requires the continued wide acceptance and adoption of the new technology, which may require additional promotional approaches. Sustainability and scalability of impacts are therefore central issues for policy and practice. Sustainability of impacts requires continued adherence by beneficiaries, solutions to “slippage” in behaviour, and financial barriers to uptake, as well as technical solutions to ensure service reliability. Scalability requires that impacts measured in small‐scale efficacy settings (the “ideal settings” measured in many field trials) are achievable in the context of programme effectiveness (“real world” settings) where fidelity of implementation becomes crucial (Bamberger et al., 2010).

The conceptual framework was used to inform how the interventions and outcomes were defined for the map and provided a logical consistency to their presentation.

### 3.2 Description of interventions

WASH interventions have four main components: “what”, “how”, “where” and “for whom”. “What” describes the WASH technology (a hardware or practice) that the programme participants gain access to (e.g., a latrine). “How” describes the intervention mechanism (e.g., a promotional campaign to motivate people to construct or purchase a latrine). “Where” describes the place of use of the technology (the household, community (shared), school or health facility). “For whom” indicates any aspect to ensure the intervention, technology or its place of use is suitable for the needs of different groups of participants (e.g., children, adolescent girls, pregnant women, people with disabilities, or people living with HIV). The principal intervention category is the intervention mechanism (the “how”), with the technology and place of use being provided as a combined filter (the “what” and “where”).

#### 3.2.1 “How”: Intervention mechanisms

Intervention mechanisms were defined so that personal and household WASH promotional approaches would be comprehensively included, and the categories defined mutually exclusive. Table 2 presents the main categories and subdivisions. Mechanisms for providing WASH technologies can be categorised into demand and supply side intervention mechanisms.^6^ Demand side intervention mechanisms include: BCC, such as health education and psychosocial “triggering”; subsidies and microloans for consumers; and legal measures targeting consumers.^7^ For example, psychosocial triggering uses psychosocial factors, principally emotions, like disgust or the desire to be a good parent (Biran et al., 2014) or social pressure, rather than reason, to motivate behaviour change among WASH consumers (De Buck et al., 2017). It aims to promote demand for a WASH technology among consumers and may use directive or participatory methods. An example of a directive approach is social marketing, which motivates social change through a combination of product (technology used to meet a need), promotion (to increase desirability and acceptability), place (installation in an appropriate place for users) and price (the cost for users takes into account affordability) (Cairncross, 2004; Evans et al., 2014). These are often implemented at community level such as in schools and health facilities via approaches such as community health clubs to promote demand (Waterkeyn & Cairncross, 2005). Participatory, bottom‐up approaches are also being rapidly scaled up, including participatory hygiene and sanitation transformation and community‐led total sanitation (CLTS). In CLTS the community is facilitated to discuss how they would like sanitation practices to change, identify problem areas (e.g., “walks of shame”), and use social cohesion and pressure to motivate people to construct latrines and stop practising open defecation (Kar & Chambers, 2008).

**Table 2:** WASH intervention mechanisms

Intervention type	Mechanism of delivery	Definition
Demand‐side	Health education	Directive hygiene, and sometimes sanitation, education where participants are provided with new knowledge or skills to improve their health based on reasoning. These information campaigns may be provided through television, radio, theatre or printed media; provided directly to specific households or through sessions at community meetings, schools or other places; or provided directly to community leaders or health workers
	Directive triggering (e.g., social marketing)	Psychosocial “triggering” covers approaches that use emotional and social cues, pressure, or motivation to encourage community members to change behaviours. Directive mechanisms are typically social marketing campaigns, which use commercial marketing techniques to promote the adoption of beneficial behaviours. They can also include other styles of campaign that use emotional or social triggers rather than information
	Participatory triggering (e.g., CLTS)	Participatory mechanisms are typically a community‐based approach and promote behaviour change through consultation with the community, a two‐way dialogue, and joint‐decision making. For example, CLTS uses this mechanism
	Subsidies and microfinance	All intervention mechanisms that use pricing reform or financial mechanisms to promote the uptake of WASH technologies. This includes subsidies, vouchers, microcredit, and other forms of microfinance, aimed at consumers
	Legal reform	Intervention mechanisms that enact or implement legal reforms proscribing open defaecation, discharge of contaminated water or dumping of waste.
Supply‐side	Direct hardware provision	The provision of any WASH hardware for free and which has been chosen by an external authority. This includes interventions where new or improved water supplies are constructed, handwashing stations are built, soap is handed out, water purifiers given away, latrines provided, or sewer connections installed by external actors (e.g., government or an NGO)
	Improving operator performance	Intervention mechanisms aiming to improve the functioning of the current service provider. This includes improving accountability, oversight or regulation, capacity building and output‐based aid
	Utility ownership	Interventions to change ownership (e.g., privatisation or nationalisation of utilities, public‐private partnerships)
	Small‐scale independent provider involvement	Intervention mechanisms to encourage small‐scale independent organisations, including nonprofits, to become the providers of WASH facilities and services on a commercial basis (e.g., sanitation marketing)
Combined interventions	Decentralisation	Focuses on putting the community at the centre of the planning, design, implementation, and operations of their service provider. Examples include community driven development, also called Social Funds, which are supposed to use a participatory approach to community decision making, provide block grants with cost sharing, and a component of local institutional strengthening to fully decentralise provision. Other approaches to involving the community but keeping government ownership include water user associations
	Combinations of intervention mechanisms	Intervention mechanisms combining multiple demand‐side (e.g., health education with subsidies), supply‐side (e.g., hardware provision with privatisation) or combining demand‐ and supply‐side mechanisms (e.g., CLTS and sanitation marketing)

Abbreviations: CLTS, community‐led total sanitation; WASH, water, sanitation and hygiene.

On the supply side, intervention mechanisms include: direct provision of technology by an external body (e.g., government, NGO); improving operator performance (e.g., institutional reform, capacity building, operator financing, legal regulation of providers, and accountability); privatisation and nationalisation of service delivery; and promoting small‐scale independent provider (SSIP) involvement (e.g., sanitation marketing through microloans and capacity building for providers). Direct provision of hardware covers all intervention mechanisms where WASH technology is provided at zero capital cost to users (e.g., Feachem et al., 1978). Hardware may be for use in private (household and yard) or public spaces (shared facilities, WASH in health facilities and schools, places of work, commerce, recreation, streets, fields and transit hubs). Measures to improve service provider performance, such as enaction and implementation of water quality standards (Cairncross et al., 1996), government regulation of private utility providers (e.g., Ministry of Foreign Affairs, 2011), and reforms to operator financing (e.g., output‐based aid or payment‐by‐results) (Trémolet & Evans, 2010). Stimulating involvement of the private sector, including privatisation (e.g., Galiani et al., 2005) (or renationalisation), contracting out to encourage involvement of the private sector and SSIPs, including nonprofits, in WASH services provision (Sansom et al., 1999), and capacity building of independent providers. As an example of the latter, sanitation marketing aims to increase availability of sanitation technology and maintenance services (such as pit emptying), by training local artisans to produce sanitation products that are suitable for the varying needs of consumers (e.g., Cameron et al., 2013).

A final group of interventions intervening on demand and supply sides includes decentralisation (Poulos et al., 2006). Decentralised delivery places community representatives at the core of the planning, design, implementation, and operation of the WASH service provider. For example, community‐driven development (CDD) uses a participatory approach, block grants with cost sharing, and often a component of local institutional strengthening (White et al., 2018). Another approach is Water User Associations, where management is devolved to the community group while government retains some powers (e.g., Barde, 2017; Waddington et al., 2019). Demand and supply intervention mechanisms may also be combined, on the demand side (e.g., where financing is combined with health messaging or BCC) and in combinations of demand‐ and supply‐side (e.g., direct provision with BCC).

#### 3.2.2 “What” and “where”: WASH technologies and places of use

The quality of water supply, sanitation and hygiene facilities—that is, the extent to which they are likely to provide drinking water of sufficient quantities for basic needs, enable hygienic hand‐washing and food preparation, and safe removal of excrement from the human environment—is dependent on the type of facility as shown in the WASH ladders (Table 3).

**Table 3:** Water, sanitation and hygiene ladders showing WASH technologies

	Drinking water	Sanitation	Hygiene
Improved facilities: safely managed	Improved facilities that:	Improved facilities where waste products are either:	Undefined
	Are accessible on premises, and Provide water when needed, and Provide water free from contamination.	Treated and disposed in situ, or Temporarily stored and then emptied and transported to off‐site treatment centre, or Transported through sewer with wastewater and treated off‐site	
Improved facilities: basic	Improved sources that require less than 30 min round‐trip to collect (including queueing time). These include piped supplies:	Improved facilities provided at the household level. These include networked sanitation:	Fixed or mobile handwashing facilities with soap and water:
	Tap water in the dwelling, yard, or plot Public standposts/pipes	Flush and pour flush toilets connected to sewers	Handwashing facilities defined as a sink with tap water, buckets with taps, tippy‐taps, and jugs or basins designated for handwashing Soap includes bar soap, liquid soap, powder detergent, and soapy water
	And nonpiped supplies:	And on‐site sanitation:	
	Boreholes/tubewells Protected wells and springs Rainwater Packaged water, including bottled water and sachet water Delivered water, including trucks and small carts.	Flush or pour flush toilets connected to septic tanks or pits Pit latrines with slabs Composting toilets, including twin pit latrines and container‐based systems.	
Limited facilities	Improved sources of the above types requiring more than 30 min to collect including queueing time.	Improved facilities of the above types shared by two or more households.	Handwashing facilities without soap and water (e.g., ash, soil, sand or other handwashing agent)
Unimproved facilities	Nonpiped supplies:	On‐site sanitation or shared facilities of the following types:	Undefined
	Unprotected wells and springs.	Pit latrines without slabs Hanging latrines bucket latrines	
No facilities	Surface water (e.g., drinking water directly from a river, pond, canal or stream)	Open defecation (disposal of human faeces in open spaces or with solid waste)	No handwashing facility on premises

*Source*: Waddington and Cairncross (2021) drawing on WHO/UNICEF (2017, 2019a) and https://washdata.org/monitoring.

An important dimension of the technology is the social and physical environment where the participants interact with it. Categorising WASH by place of use emphasises the differential effect, and potentially different causal pathways, of providing the same technology in different locations (Table 4). Place of use affects acceptability and convenience to users, and therefore adoption rates, as well as how the intervention disrupts the causal chain of disease transmission.

**Table 4:** WASH technologies and subcategories

Technology	Subcategories	Definition
Water supply	Household provision, community provision, at a school or health facility	Provision of a new or improved water supply or distribution system. Community provision includes shared boreholes or standpipes. Household provision includes water piped directly to the home and yard, or individual rainwater tanks, or standpipes in rural areas. Other important places of provision include schools and health care facilities
Water treatment and storage	Household provision, community provision, at a school or health facility	Supplies for, and information on, water treatments either to remove microbial contaminants or to maintain water quality by enabling safe water storage practices. Examples of water treatment technologies include filtration, chlorination, flocculation, solar disinfection, boiling, and pasteurising. Water quality improvements are most commonly undertaken at the household level. Household provision covers practices at home (POU) and when transporting water privately from a communal water point (e.g., provision of hygienic water containers). Community provision includes treatment provided at a communal water access point or protection of water from contamination at source (e.g., a protected spring). Other subcategories cover practices in schools and health care facilities
Sanitation hardware	Latrines for household use, communal use, at a school, or health facility	New or improved hardware for latrines or other means of excreta disposal. This is divided into communal or shared latrines (used by two or more households), those in a private home (used by a single household), and those in schools and health care facilities
	Safe waste disposal system	Connecting the existing means of excreta disposal to a public sewerage or other drainage system. It could also cover a desludging or faecal waste management service, comprising the collection, transportation and treatment of waste from latrine pits and septic tanks
Hygiene	Handwashing supplies for household use, at a school or health facility	Soap, similar products (e.g., hand sanitiser), and/or a handwashing station with information on how to correctly use them. These can be provided for use at home, at a school, or at a health facility
	Improved handwashing practices	Knowledge of the best practices for handwashing. This is a software and therefore its use is not tied to a specific physical location
	Menstrual care	Knowledge of the best practices for menstrual hygiene management and the supply, or use, of sanitary products, including pads and tampons
	Other improved practices including personal and food hygiene	Knowledge of the best practices for other hygiene techniques or procedures (including face washing and latrine cleaning), as well as the supply, or use, of toilet paper or other hygiene products. This category also includes personal food hygiene practices beyond handwashing at appropriate times. This includes covering and storing food properly and washing dishes effectively
Combined	Combined WSS	Programmes that provide water supply and sanitation technologies
	Hygiene combined with water and/or sanitation	All other programmes that provide multiple technologies. These may be tied to a physical location depending on the technology (e.g., latrines and handwashing supplies at a school)

Abbreviations: POU, point‐of‐use; WSS, water supply and sanitation.

For example, hygiene behaviour change is more likely to break disease transmission in households, whereas investments in infrastructure such as drains and excreta disposal systems are more likely to affect disease transmission in public spaces (Cairncross et al., 1996). Factors of control are likely to be weaker in community settings than institutional settings, such as schools and day care centres, where simple hygiene messaging and “behavioural nudges” are more likely to be effective (e.g., Ryan et al. 2001). The four main spaces in which WASH technologies are provided are the home and yard (for use by an individual household), the community (spaces shared by two or more households, including fields, streets and places of work, commerce and recreation), at school, and at a health facility. The evidence map therefore includes filters for the WASH technology provided and the place of use, as well as location (rural, urban, informal (peri‐) urban settlement and refugee camp).

#### 3.2.3 “For whom”: WASH technology users

**Figure 5:**
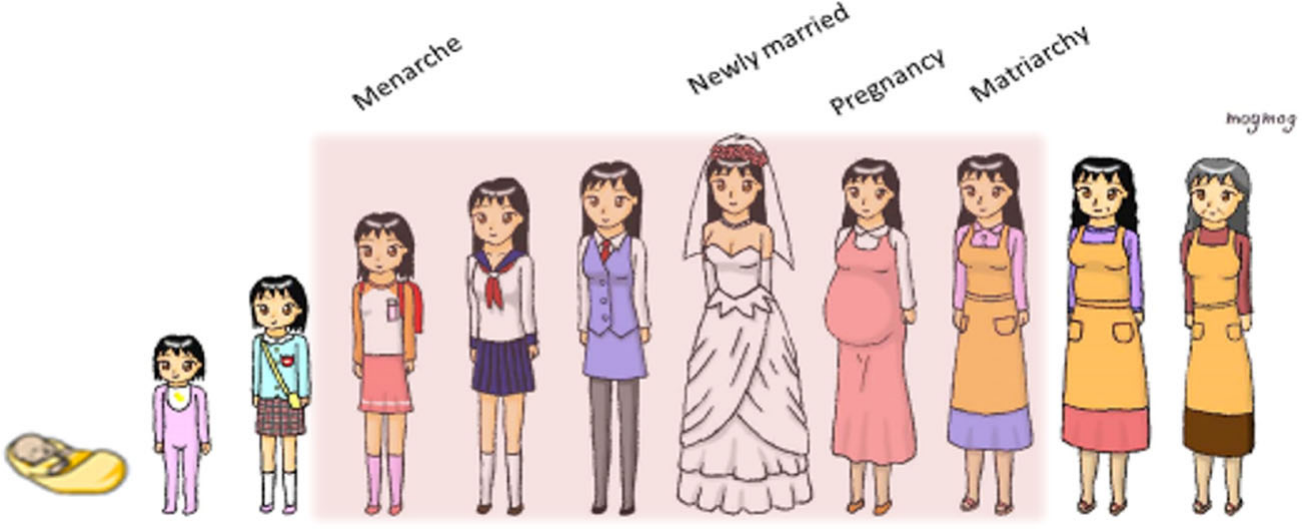
Female reproductive health over the life course. *Source*: Water Supply and Sanitation Collaborative Council

The final relevant dimension for the intervention is the suitability of the WASH technology to different users. For example, women's needs change over their life‐cycle, hence WASH service provision needs to be suitable for different points in the reproductive life‐cycle, including menarche (e.g., separate toilets for girls at school, promotion of menstrual hygiene management approaches) and maternity (e.g., WASH in health facilities, promotion of hygienic weaning practices) (Figure 5). Caruso et al. (2017) define sanitation insecurity as “[i]nsufficient and uncertain access to socio‐cultural and social environments that respect and respond to the sanitation needs of individuals, and to adequate physical spaces and resources for independently, comfortably, safely, hygienically, and privately urinating, defecating, and managing menses with dignity at any time of day or year as needs arise” (p. 9). Other group who are disadvantaged or vulnerable may also have particular needs, such as water and sanitation facilities for the elderly and infirm, or drinking water treatment for immunocompromised people (e.g., those living with HIV). For example, walkways may need to be constructed to prevent falling and elevated seats or rails installed to help elderly people, those with disabilities, and pregnant women (Caruso et al., 2017).

### 3.3 Description of outcome categories

The consequences of WASH interventions can be grouped into intermediate outcomes (behaviours) and quality of life outcomes (water‐related ill‐health, other health, nutrition, mortality and socioeconomic outcomes). Table 5 lists the outcome constructs included in the map, together with examples of indicators used to measure them. Unlike the initial WASH evidence map (Waddington et al., 2014), primary outcomes also included behaviours:

a. Time use (e.g., measured or reported time spent collecting water, defaecating, undertaking childcare, working, sleeping);
b. Water quantity used, and quality of water supply (e.g., free of chemical contamination such as arsenic);
c. Water treatment practices (e.g., reported or measured chlorination);
d. Latrine use or defaecation practices (including construction of facilities for “triggering” intervention mechanisms);
e. Hygienic behaviour (e.g., observed hand washing practices, measurement of hand contamination);
f. Sustainability or slippage back to practices like open defaecation (measured 12 months post‐intervention) and
g. Willingness‐to‐pay.

**Table 5:** Outcome categories and indicators

Outcome	Subcategory	Example indicators
Behavioural	Water supply behaviour	Water source used
		Water source free of chemical or pathogen contamination
		Litres of water collected or consumed daily
	Time use	Time spent collecting water
	Water treatment and storage practices	Observed or reported water treatment practices
		Residual chlorine in drinking water
		Microbial contamination of water
	Construction, use, and maintenance of latrines	Latrine construction (for triggering intervention mechanisms)
		Reported or observed use of latrines
		Observed cleanliness of latrines
	Open defecation	Reported or observed frequency of open defecation
	Hygiene behaviour	Observed or reported handwashing
		Reported use of sanitary pads or other sanitary product
		Observed covering of food
		Microbial contamination of hands, food, fomites etc.
	Willingness‐to‐pay	Amount willing to pay for a product or service, for example, water filter, desludging service or piped water supply
	Sustainability and slippage	Behavioural outcomes measured a year or more after the completion of intervention activities
Water‐related ill‐health	Diarrhoeal disease	Self‐ or carer‐reported diarrhoea defined as three or more loose or watery stools in a 24‐h period over a 2‐, 7‐ or 14‐day recall
		Frequency of medical treatment for diarrhoea (hospital records)
	Acute respiratory infection	Self‐ or carer‐reported cold or flu‐like symptoms defined, for example, as sore‐throat, cough or fever over a 2‐, 7‐ or 14‐day recall
	Other water‐related infections	Trachoma measured as observed inturning eye‐lash or ocular chlamydia test
		Number of parasitic worm eggs in stool
		Malaria infection
Other health	Drudgery, pain, and musculoskeletal disorders	Reported number of days when pain prevented the accomplishment of daily tasks
	Psychosocial health	Self‐reported happiness
		Self‐reported insecurity
		Self‐reported shame or dignity
		Reported feeling unsafe or vulnerable in the last month when collecting water or defecating
Nutrition	Nutrient intake and growth	Height‐for‐age *z* score (HAZ), weight‐for‐age *z* score (WAZ), weight‐for‐height *z* score (WHZ)
		Body mass index (BMI)
		Rate of anaemia
		Chronic malabsorption and food wastage (enteropathy)
Mortality	All‐cause or cause‐specific mortality	All‐cause mortality or mortality due to ARIs, diarrhoea or other infection among neonates, infants or under‐5s
Socioeconomic	Education and cognitive development	Number of days missed from school
		Scores on school tests
		Children reaching a developmental milestone
		Time spent on adult education
	Labour market and employment	Employment, including employment of women
		Wage rate
	Income, consumption, poverty	Household income in the last month
		Proportion of households living in extreme poverty
		Healthcare and aversion costs
		Time poverty
	Political engagement	Electoral outcomes
		Number of women participating in local government

### 3.4 Description of the geographic, population and study design categories

The EGM also contains several filters to further break down the evidence. There are two filters that provide geographic information at the region and country level, as well as a population filter that allows a user to explore specific target groups. The populations covered are rural, slum (or informal settlement), urban, humanitarian crisis, refugee camp, and measurement of outcomes among groups who have been marginalised including people people living with HIV and people living with disabilities. The final filter is study design, which categorises the impact evaluations into three broad categories: RCTs, nonrandomised design and natural experiments using observational data with “selection on unobservables” (see below).

### 3.5 Stakeholder engagement

Evidence maps should ideally be developed in a participatory way, by drawing on end users at the study design phase through stakeholder engagement processes. Stakeholder engagement was sought from organisations providing sector policy and programmes implementation and/or support, on the design of the evidence matrix and the inclusion criteria. Stakeholders included staff at the Aga Khan Foundation, Sanitation and Hygiene Applied Research for Equity (SHARE) consortium, WaterAid and WSSCC. The preliminary findings were also presented to staff at WSSCC.

## 4 METHODS

### 4.1 Criteria for inclusion and exclusion in this review

A protocol for the WASH evidence map was published in the Campbell library (Waddington et al., 2018). Table 6 summarises the criteria for inclusion of populations, interventions, comparators, outcomes and study designs (PICOS), as well as language and time frame.

**Table 6:** Summary of inclusion criteria

Criteria	Definition
Populations	Human populations in LMICs, as defined by the World Bank at the time the research was carried out. Populations of any age, sex, gender, disability or socioeconomic status were included. Populations in epidemics (e.g., cholera outbreak) were excluded
Interventions	Demand‐side (behaviour change communication, subsidies, microloans, legal measures), supply‐side (direct hardware provision, privatisation and nationalisation, small‐scale independent provider involvement, improved operator performance), or combinations of demand and/or supply (e.g., decentralisation)
	Technology and place of use: water supply, water quality, sanitation, and/or hygiene in the household, community, school or health facility
Comparators	Impact evaluations where the comparison/control group receives no intervention (standard WASH access), a different WASH intervention, a double‐blind placebo (e.g., nonfunctioning water filter), a single‐blind (e.g., school textbooks), or a pipeline (wait‐list)
Outcomes	Behaviour, health, and socioeconomic outcomes. Studies that only reported measures of knowledge or attitudes were excluded. WTP was included where based on real purchase decisions
Study design	Randomised controlled trials, prospective and retrospective nonrandomised studies, natural experiments, and systematic reviews. For time use outcomes only: the above plus reflexive controls. For mortality outcomes only: the above plus case‐control designs
Language	Studies in English, French, Spanish and Portuguese. Studies in other languages were included where an English translation was available
Time frame	No study was excluded based on date of publication

Abbreviations: LMIC, low‐ and middle‐income country; WTP, willingness‐to‐pay.

#### 4.1.1 Type of populations

Studies were included on any population in LMICs, as defined by the World Bank at the time the research was carried out. Populations of any age, sex, gender, disability or socioeconomic status were included, provided the study was conducted in endemic conditions found regularly in LMICs. Hence, studies that were conducted under outbreak conditions, such as cholera epidemics, were excluded (e.g., Daniels et al., 1999; Snow, 1855).

#### 4.1.2 Types of interventions

Studies were included that measured receipt of a clearly defined WASH intervention.^8^ Tables 2 and 4 summarise eligible WASH intervention mechanisms and technologies. The evidence map covers interventions to promote WASH for household and personal consumption. It excludes interventions in food hygiene in the workplace such as a market (e.g., Sobel et al., 1998), methods to control faecal contamination by animals in the yard (e.g., Oberhelman et al., 2006), and vector control methods such as fly spraying (e.g., Chavasse et al., 1999; Emerson et al., 1999). Interventions primarily supporting farms or businesses such as dam construction (e.g., Duflo & Pande, 2007) were also excluded, as were interventions for groundwater or irrigation management (e.g., Meenakshi et al., 2013). Likewise, flood and drought management interventions and river, lake, coastal zone and wetlands management were omitted. The map also excludes studies where there was no clear intervention being provided, such as the association between shared versus private sanitation on diarrhoea (Baker et al., 2016). Exclusion of nonintervention studies unfortunately omitted studies measuring uncommon outcomes like preterm births and low birth weight (Olusanya & Ofovwe, 2010). Finally, the map excluded cointerventions with a major non‐WASH component. This typically excluded deworming chemotherapy (e.g., Miguel & Kremer, 2004) and nutrition interventions (e.g., Humphrey et al., 2019), although any WASH‐only trial arms without cointerventions of such studies were included (e.g., Luby et al., 2018; Null et al., 2018). WASH interventions at medical facilities were included if they met the above intervention definitions. Studies on medicalised hygiene (such as sterilising wounds) were excluded.

#### 4.1.3 Types of outcome measures

Studies measuring outcomes among human populations were included. Studies that collected outcomes on environmental or animal subjects were excluded, for example, those measuring the efficacy of water treatment technology in the environment (e.g., Crump et al., 2004). Table 5 lists eligible outcomes, which included behaviours (e.g., time savings from improved water availability), water‐related ill‐health (e.g., diarrhoea and ARIs), other ill‐health (e.g., musculoskeletal injury, stress), nutrition (e.g., height‐for‐age), mortality, and socioeconomic outcomes (e.g., income, education, employment). To be included, the outcome needed to clearly relate to a WASH intervention mechanism or exposure. For example, where some programme evaluations of CDD—an approach that is used to provide projects in multiple sectors such as infrastructure, education and health—did not give estimates of outcomes separately for WASH projects, these outcomes were excluded from the map (e.g., diarrhoeal infection in Beath & Enikolopov, 2013).

All measures of intervention outputs, such as participant knowledge, awareness and attitudes were excluded from the map, which is concerned with programme effectiveness, rather than implementation. Facility access was only included when an intervention mechanism promoted construction of latrine, water supply or hand‐washing facilities. This means, for example, that studies of direct hardware provision reporting only latrine ownership, or knowledge resulting from hygiene education, or awareness and attitudes (e.g., Stockman et al., 2007), were omitted. In contrast, studies (and outcomes in such studies) were included that measured latrine ownership (e.g., Guiteras, Levinsohn, et al., 2015) in the context of latrine promotional intervention mechanisms (CLTS, subsidies).

Willingness‐to‐pay was included where the method used real purchase decisions and was excluded where based on hypothetical scenarios which are unreliable, partly because survey respondents may strategically overstate it (Null et al., 2012; Whittington, 2002). Where studies collected data on pre‐existing health status, which was subsequently used in stratified analysis, such as reporting differential effects on stunted versus nonstunted children (Luby et al., 2005), pre‐existing health was not included as an outcome or population filter.

Adverse outcomes were eligible but no studies reported any; for example, the effect of hygiene on reducing microbial exposure, potentially leading to rise in allergy and auto‐immune diseases, also known as the “hygiene hypothesis” (Bloomfield et al., 2016; Strachan, 2000).

#### 4.1.4 Types of evidence

The evidence map includes impact evaluations and systematic reviews of the effectiveness of WASH intervention mechanisms and technologies. Impact evaluations were defined as programme evaluations or field experiments that used quantitative approaches applied to experimental or observational data to measure the average effect of participating in a programme relative to a control or comparison group (counterfactual) representing what would have happened to the same group in the absence of the programme. Eligible impact evaluations also tested different intervention mechanisms or technologies (i.e., active controls). Both completed and on‐going impact evaluations and systematic reviews were searched for and included.

The following study designs were included:

(1) Studies explicitly described as systematic reviews or meta‐analyses, which synthesised evidence on effectiveness of WASH intervention mechanisms or exposures, and described methods used for searching, data collection. For completeness, two early literature reviews on which much of the subsequent systematic review literature is based, were also included (Esrey et al., 1985, 1991). Systematic reviews of effectiveness were eligible, not those addressing other aspects such as prevalence of child faeces disposal in LMICs (Gil et al., 2004).
(2) Prospective quantitative evaluations where participants were assigned to intervention(s) at individual or cluster levels:
  a. RCTs with randomised assignment of units at individual and household level (e.g., Han & Hlaing, 1989), or with cluster assignment at a higher level (village, township, school or health facility) (e.g., Clasen et al., 2014a), quasi‐RCTs using quasi‐randomised assignment of units (e.g., alternation of clusters listed alphabetically), and studies using randomised encouragement, providing promotional information about an intervention or technology that is universally available (e.g., Devoto et al., 2012).
  b. NRS with assignment of units based on practitioner or participant selection and contemporaneous measurement of outcomes by investigators at pre‐ and posttest in treatment and comparison groups,^9^ or contemporaneous measurement by investigators in treatment and comparison group at posttest only. These include prospective cohort studies that used methods such as statistical matching (e.g., propensity score matching) (e.g., Reese et al., 2017), or direct control for confounding in adjusted analysis (e.g., Cole et al., 2012). Studies of interventions that used “naïve” matching of communities were eligible where outcomes were compared to a group receiving an intervention to a geographically separate comparison group, and some information was reported on the treated and comparison groups (e.g., Shiffman et al., 1978). However, cross‐sectional studies that analysed the relationship between WASH technology interventions and outcomes, which compared self‐selected participants within the same group, but did not use any methods to control for confounding (e.g., Gross et al., 1989) were excluded.
  c. NRS with measurement by investigators in treatment group at least six time points pre‐ and posttest (interrupted time‐series [ITS]) (Fretheim et al., 2015).
  d. Cross‐over trials where treatment and control or comparison are swapped (e.g., Kirchhoff et al., 1985).
(3) NRS designed retrospectively—that is, after intervention has occurred—with selection on observables, including nonrandomised pipeline design (e.g., Cairncross & Cliff, 1987), studies using cross‐section data (e.g., Khan, 1987) and studies using panel data or pseudo‐panels of repeated cross‐sections with an intervention and comparison group, using methods to match individuals and groups statistically or control for observable confounding in adjusted analysis (e.g., Galiani et al., 2005).
(4) Natural experiments designed retrospectively with selection on unobservables:
  a. Natural experiments using exogenous treatment assignment rules, including randomised natural experiments (with assignment by public lottery), and natural experiments where assignment was by random errors in implementation.
  b. Regression discontinuity designs (RDDs) with prospective assignment to intervention and comparison groups based on a threshold on a continuous variable (e.g., number of cases of disease in a community, poverty index) or a physical threshold such as an administrative boundary (e.g., Spears, 2013; Ziegelhofer, 2012).
  c. Studies using multistage or multivariate approaches with identification of compliers based on exogenous variation (e.g., instrumental variables [IV]).
  In addition, the following study designs were included for specific outcomes:
(5) For time savings, reflexive controls with prospective measurement at pre‐ and posttest (but no comparison group). Time savings associated with improved WASH are immediate outcomes, a very short way along the causal pathway from intervention, for which the expected effect is large and confounding is unlikely (Victora et al., 2004). Reflexive control studies were excluded that measured other outcomes like water treatment behaviour (e.g., Makutsa et al., 2001), hygiene (Onyango‐Ouma et al., 2005), latrine use (e.g., Murthy et al., 1990), urinary arsenic levels (Chen et al., 2007), diarrhoea pathogens (e.g., Kariuki et al., 2012) and incidence of gastro‐intestinal disease (e.g., Zaheer et al., 1962).
(6) For studies measuring mortality, case‐control designs, and other types of studies of WASH exposures, were included, provided they referred to a specific intervention. This is because of ethical concerns around collecting mortality data in prospective intervention studies. Any other eligible intervention studies reporting mortality were also included. However, any case control studies analysing mortality not associated with a particular WASH intervention were excluded (e.g., Hoque et al., 1999; Victora et al., 1988). Other outcomes reported in case control studies were excluded. This excluded, for example, a set of studies examining the relationship between shared versus private latrine and diarrhoea morbidity in Bangladesh, the Gambia, India, Kenya, Mali, Mozambique and Pakistan (Baker et al., 2016). Case control studies using modelling to estimate diarrhoea‐related mortality were also excluded (e.g., Birmingham et al., 1997).

Study designs that were not related to a clearly defined intervention were excluded (e.g., Feachem et al., 1978; Jalan & Ravallion, 2003; Root, 2001; Wagner & Lanoix, 1959), or those without a comparator receiving a different intervention or service (e.g., Israel, 2007). This excluded some natural experiments where no intervention could be identified (Geruso & Spears, 2018).

Studies, or components of studies, that collected and analysed purely qualitative evidence were excluded. For example, in a controlled study of slum upgrading by Parikh and McRobie (2009) in Gujarat, India, women reported saving time and labour, and having fewer back problems, as a result of no longer having to carry buckets of water. This information was collected using qualitative interviews and presented in quotation rather than quantitatively; therefore, it was excluded.

#### 4.1.5 Search

Systematic searches were done for both published and “grey” (i.e., nonpeer reviewed) literature. The existing electronic database searches for the 2014 EGM and a 2017 systematic review, on hygiene and sanitation behaviour change intervention mechanisms (De Buck et al., 2017), were updated to March 2018. Searches were also run to cover the rest of the extended scope, particularly water behaviour change and WASH in health care facilities. All search word lists were developed by an information retrieval expert and, in February 2018, eleven academic databases and four trial registry databases were searched (Supporting Information Appendix A). To capture grey literature, hand searches were conducted of key organisation websites. These included the Impact Evaluation Repository of the International Initiative for Impact Evaluation (3ie), the Asian Development Bank, African Development Bank, Inter‐American Development Bank, Department for International Development, IMPROVE International, IRC (WASH),^10^ Oxfam, UNICEF, US Agency for International Development, WaterAid, and the World Bank. Finally, the bibliographies of all included systematic reviews were checked to identify additional primary studies and systematic reviews. Reference lists of books, reports and evaluations were searched to identify additional WASH impact studies, particularly earlier ones that may not be captured in electronic searches (Briscoe et al., 1986; Cairncross et al., 1980; Esteves Mills & Cumming, 2016; Feachem et al., 1978; Khan et al., 1986; Saunders & Warford, 1976; White & Gunnarson, 2008; White et al., 1972; WHO, 1983). Finally, forward citation tracing searches were done in May 2020 for impact evaluations and systematic reviews that were identified as ongoing in 2018 and had since been completed.

### 4.2 Screening and study selection

EPPI‐reviewer 4 software was used to manage the screening process (Thomas et al., 2010). Once duplicates had been removed, there were 13,458 records for screening at title and abstract stage. To reduce resource requirements needed to screen this many studies at the title and abstract stage, machine learning was employed.

The process of conducting systematic searches is becoming more and more demanding as more evidence is produced and more databases that require searching become available (Waddington et al., 2018). Hence, much of the time spent in conducting a systematic review or evidence map is absorbed by the process of searching and screening the available literature, often using word‐recognition processes, with little time left for evaluating and synthesising the evidence. A large amount of researcher effort can be spared if we are willing to accept: (a) that studies can be classified by a relevance score produced by a machine algorithm; and (b) a reasonable margin of error in screening, which is likely to result in excluding some relevant studies.^11^

**Figure 6:**
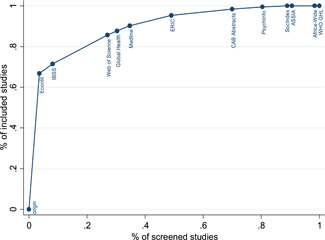
Sensitivity and precision in systematic searches. *Source*: Masset (2020)

Figure 6 is an illustration of the potential for improvement. It shows the percentage of studies (vertical axis) as a function of the percentage of screened studies in each search database (horizontal axis) included in a recent review. The search databases were ordered in the figure according to the precision (percentage of included studies over percentage of studies identified). The searches in the review were designed to be sensitive, meaning that they aimed to identify as many relevant studies as possible. The figure suggests that 20% of the searches delivered 80% of the studies included. It also suggests that, had the authors been willing to undertake searches with greater precision, omitting 20% of the evidence, they could have conducted the search in a fifth of the time.

The problem with this example is that researchers do not know how many studies will be included and excluded from each database before conducting the search. The figure was calculated after the review was completed. However, methods are available to estimate the total population of studies. For example, two early reviews of the effect of household water treatment on diarrhoea were incomplete: Fewtrell and Colford (2004) contained 13 studies, Gundry et al. (2004) contained 12, but only five studies were common to both reviews. By considering the two studies as a “mark‐release‐recapture” experiment (Krebs, 2014), this suggested a universe of 28 studies (95% confidence interval [CI] = 18, 88) which could be detected using an improved search strategy. A subsequent review conducted shortly after found 32 household water treatment studies (Clasen et al., 2007b).

**Figure 7:**
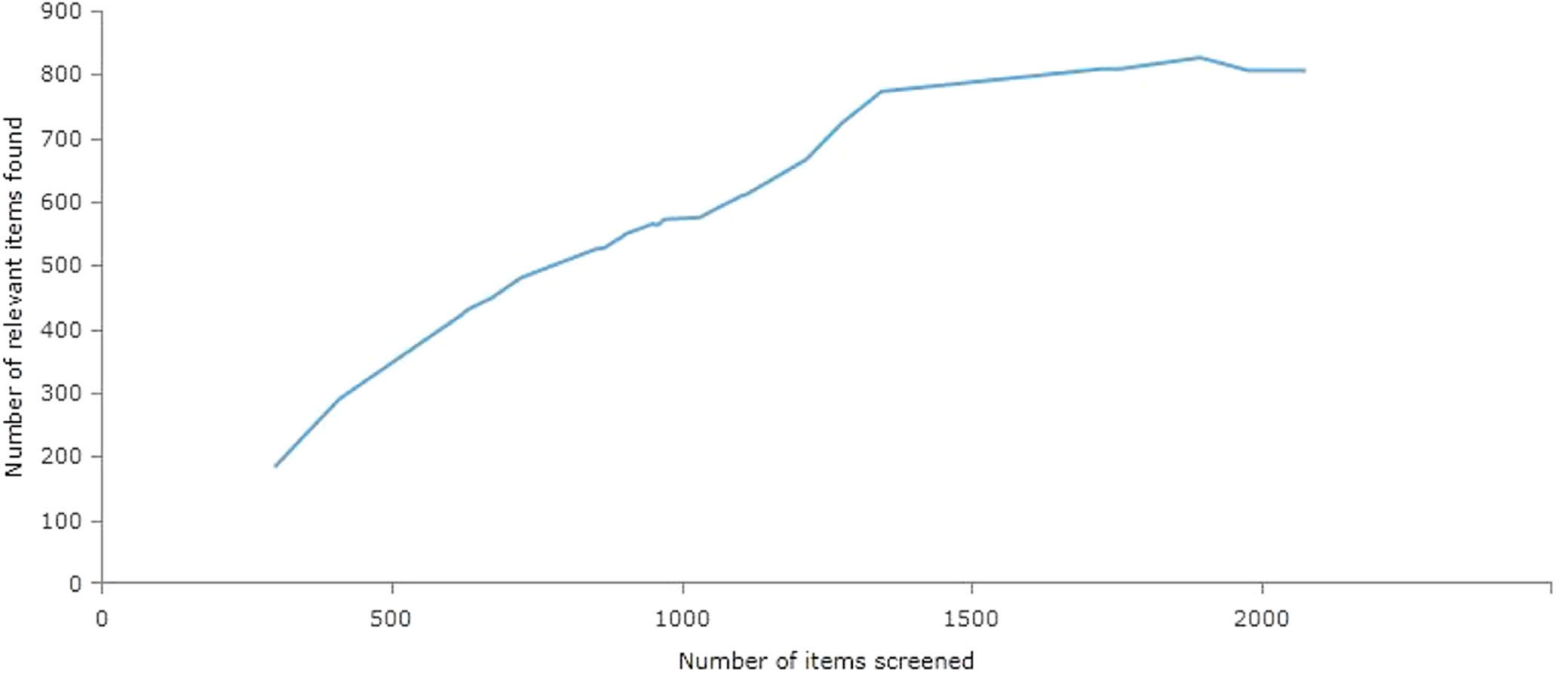
Application of machine learning in WASH evidence searches. The negative gradient in the curve at the 1900 studies screened point was due to the decision taken to deviate from protocol by excluding non‐WASH cointervention studies. *Source*: EPPI‐reviewer 4 (Thomas et al., 2010)

The machine learning software, which is integrated into EPPI‐Reviewer, functions by identifying key words, through text mining, in included and excluded records and then ranking studies from most to least likely to be included. This can be updated at regular intervals to reflect more recent inclusion decisions. Other studies looking at the effectiveness of this software found that it can often save up‐to 70% of the work‐load with a loss of only 5% of the includable studies (O'Mara‐Eves et al., 2015). In the first stage, a sample of 300 studies was used to train the algorithim before it began running. After removing duplicates, two authors screened the records at the title and abstract stage until they did not find a single includable study for 100 consecutive records in the list ranked by relevance (Figure 7). A random sample of 100 of the remaining studies was then screened to increase confidence that no studies had been missed. Ultimately 1798 records were manually screened, which was an estimated workload saving of almost 90%. Two authors then screened the remaining papers at full text to determine inclusion in the map.

There are of course many reasons why systematic reviews on water, sanitation and/or hygiene might include different studies, or not be undertaken based on independent searches. Most obviously, included interventions or primary outcomes may differ. For example, many reviews have been restricted to health impacts like diarrhoea (e.g., Clasen et al., 2015; Waddington et al., 2009; Wolf et al., 2018), while a few others focus primarily on behavioural outcomes (e.g., De Buck et al., 2017; Garn et al., 2017). Or study design inclusion criteria may differ, with some restricting inclusion to studies evaluating a particular intervention (e.g., Clasen et al., 2015; Wolf et al., 2018) and others including exposures as well (e.g., Curtis & Cairncross, 2003; Heijnen et al., 2014; Waddington et al., 2009). In addition, there is a growing tradition of updating systematic reviews for new studies, so searches are not independent. Most recently, the systematic review of WASH and diarrhoeal morbidity by Wolf et al. (2018) updated searches and analysis done by Wolf et al. (2014), which itself was designed based on comprehensive reviews on the same topic by Waddington et al. (2009) and Cairncross et al. (2010). Waddington et al. (2009) was in turn an explicit update of Fewtrell and Colford (2004), which itself updated Esrey et al., (1985, 1991). Cairncross et al. (2010) originated from Curtis and Cairncross (2003) and Clasen et al. (2006).

Two recent reviews that did systematically search for the same intervention and outcomes—evaluations of the effect of sanitation promotion interventions on behaviour change—are De Buck et al. (2017) and Garn et al. (2017). As far as it is possible to tell, these reviews were done independently, as neither cites the other.^12^ Thirty‐seven sanitation promotion studies were contained in the two reviews, of which only nine were common to both. De Buck et al. (2017) included 18 studies, while Garn et al. (2017) included 28. Part of the reason for the difference is that Garn et al. (2017) were more inclusive on design, including, in addition to contemporaneously controlled evaluations, reflexive controls (pre‐ and posttest only). Based on these findings, we estimated there would be 55 studies in total (95% CI = 39, 101). Once again, this estimate is remarkably accurate: the searches undertaken for the evidence map found 53 studies of sanitation behaviour change.^13^

#### 4.2.1 Excluded studies

At the title and abstract stage, most studies were excluded because they were clearly not related to WASH, they used reflexive control (uncontrolled before‐after study) design without collecting data on time‐savings, or they were an efficacy or laboratory‐based study. One ITS study of hygiene promotion in Burkina Faso with three preintervention measurement rounds but only one post intervention round measuring of children and mother's latrine and hygiene behaviour was therefore excluded (Curtis et al., 2001). Nearly half the papers were excluded based on study design at full text stage; this was often because they were studies of WASH exposures not intervention mechanisms. While exposure studies are useful in establishing the relationship between WASH infrastructure and practices and health outcomes (e.g., Geruso & Spears, 2018; Jalan & Ravallion, 2003), they do not provide evidence of the intervention mechanisms used to improve WASH access and use. The second biggest reason for exclusion was on intervention, where the WASH component could not be isolated as it was provided with other non‐WASH cointerventions. For example, the Sanitation, Hygiene, Infant Nutrition Efficacy (SHINE) trial (Humphrey et al., 2019) combined WASH with nutrition. The WASH Benefits trials (Luby et al., 2018; Null et al., 2018) also intervened in nutrition and WASH, but had trial arms with only WASH interventions, which were eligible for inclusion in the map. Beyond combined WASH and nutrition interventions, the other common combination was WASH with deworming medication, a famous example being Miguel and Kremer (2004) which incorporated a hygiene intervention. Most of the studies excluded for outcome measured only knowledge or attitudes. To avoid duplication, 44 papers were excluded because they were a different version of a study already included. Finally, 41 studies were excluded because they could not be located (e.g., the search had found only a conference abstract with insufficient detail to code the study in full) or accessed in full text. A complete list of the studies excluded at full text is included in the references section.

### 4.3 Data collection and analysis

A standardised data extraction form was used to collect descriptive data from all the included impact evaluations. This included bibliographic details, intervention mechanisms and WASH technologies, outcomes, study design, geographic information and populations targeted. Summary data were collected from each included study on the six areas that Blum and Feachem (1983) had identified as being suboptimal in impact evaluations of WASH and diarrhoea: use of a control group, adjustment for confounding, definition of the outcome, length of recall, analysis of use and (individual and cluster) sample size. In addition, information on basic reporting information was collected from prospective studies (presentation of participant flow diagrams or the data from which to reconstruct them), and whether basic ethical requirements were met through institutional review board (IRB) approval. Supporting Information Appendix B presents the data extraction form.

In addition to the above, all systematic reviews underwent a critical appraisal. Presented in Supporting Information Appendix C, this critical appraisal tool drew on the SUPPORT critical appraisal tool (Lewin et al., 2009) and and AMSTAR2 (Shea et al., 2016), which evaluate protocol, search, screening, analysis and reporting methods, to produce an overall rating of low, medium, or high confidence in the review findings. It also aimed to incorporate best practices in WASH programme evaluation by assessing use of an explicit theory of change, collection of outcomes along the results pathway, and systematic incorporation of qualitative evidence such as on programme implementation (Jimenez et al., 2018). The data extraction for each study was done by two authors. Extensive piloting was also conducted to ensure consistency and agreement in coding.

### 4.4 Analysis and presentation

The remainder of this report is structured as follows. The results sections present the results of the search and a summary of findings in the areas of impact evidence and systematic evidence, respectively, together with the major evidence gaps. The discussion section provides an analysis of the trends observed in the research literature and possible implications of this. Information on critical appraisal of systematic reviews is in Supporting Information Appendix D. The Supporting Information Data Appendices (provided as spreadsheets) present summary information on all included systematic reviews and impact evaluations.

#### 4.4.1 Visualisation and analysis

The online, and interactive, EGM provides a matrix of intervention mechanisms against outcomes as described in Section Objectives, 2. In brief, the matrix displays intervention mechanisms (e.g., direct hardware provision) against outcomes along the causal pathway (from behaviour change to socioeconomic impacts). There are then several filters to further breakdown the evidence. The most important of these is the WASH technology filter (e.g., latrines for household use), but other filters include region, country, study design (RCT, nonrandomised study, or natural experiment), and population (e.g., people living with HIV or disability).

This report presents the major trends in the interventions that were researched, outcomes reported, participants being targeted, and the findings from the systematic reviews. It was decided to use 2008 as a cut‐off to present the overview of research in the WASH field, demarcating “first period” and “second period” rigorous evaluation studies. The International Year of Sanitation in 2008 brought attention to the importance of sanitation technologies to the policy and research communities, and catalysed funding for WASH evaluations from traditional and nontraditional organisations (e.g., Gates Foundation) in an area that was previously considered too costly for impact evaluations to be applied (Cairncross et al., 2014).

### 4.5 Study dependency

Where multiple papers existed on the same study (e.g., a working paper and a published version), the most recent open access version was included in the evidence map. If the versions reported on different outcomes, an older version was included for the outcomes not covered in later versions.

### 4.6 Deviations from protocol

No deviations from protocol were made in study inclusion and data collection. However, changes to intervention mechanism groupings were made, to enable interventions to be classified by demand and supply. In addition, critical appraisal of study designs was added for impact evaluations drawing on categories originally proposed by Blum and Feachem (1983). The critical appraisal of systematic reviews used a more comprehensive critical appraisal tool than originally envisaged (Jimenez et al., 2018).

## 5 RESULTS

### 5.1 Description of studies

#### 5.1.1 Search results

The number of rigorous impact evaluations and systematic reviews of water, sanitation and hygiene interventions has grown exponentially over both the last decade and even in the years since the initial WASH EGM was produced (Waddington et al., 2014). In this section, we use the International Year of Sanitation, 2008, as the cut‐off marking a “behavioural revolution” in impact studies and systematic reviews in the WASH sector. At the time the searches were completed, there were 337 completed and 46 on‐going impact evaluations using quantitative counterfactual methods in LMICs, nearly three‐quarters of which had been conducted after the end of the International Year of Sanitation, from 2009 onwards. There were also 42 completed systematic reviews and three protocols, of which all but four had been published since 2008. Since the initial WASH evidence map was published, at least 250 additinoal impact evaluations have been found, and 20 systematic reviews have been completed. One RCT of hand sanitiser produced by Proctor and Gamble, which had been identified in a previous systematic review by the author, remained unpublished (Odio et al., 2004). Sufficient information was available in that study's abstract for inclusion in the map. In total, therefore, there are at least 359 completed and 22 on‐going impact evaluations of WASH interventions in LMICs, nearly three‐quarters of which have been completed since 2008. There are also at least 43 systematic reviews and 2 protocols, of which all but four were completed after 2008.

Searches were also done in 2020 to locate impact evaluations and systematic reviews that had been found in trial and protocol registries in 2018, which had since been completed. These searches found 20 impact evaluations had been published by May 2020 (Acey & Norman, 2017; Armand et al., 2020; Augsburg & Oteiza, 2014; Batmunkh et al., 2019; Chauhan et al. (for Curtis et al., n.d.); Cocciolo et al., 2017; Viswanathan et al., 2020; Delea et al., 2020 (for Freeman et al., 2017); Trent et al., 2018; Dreibelbis et al., 2019; Dupas et al., 2016; Friedrich et al., 2018; Gray, 2019 (for Stewart, 2013); McGuinness et al., 2017; Nagel et al., 2016; Oviedo & Rounseville, 2017; Peletz et al., 2019; Rabbani, 2015; Reese et al., 2017; Vijayaraghavan & Kilroy, 2017), and one systematic review (Majorin et al., 2019).^14^

**Figure 8:**
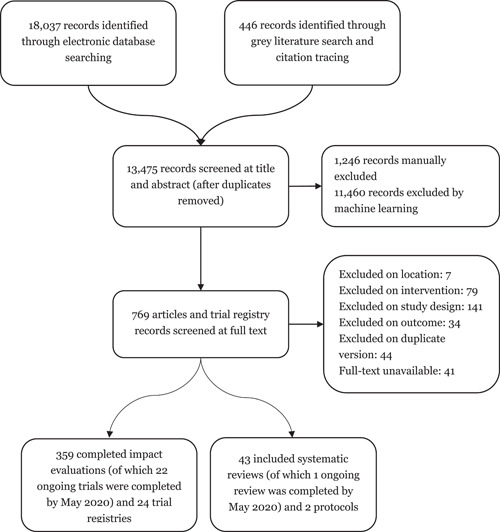
PRISMA study search flow diagram

Figure 8 presents the preferred reporting items for systematic reviews and meta‐analyses (PRISMA) study search flow diagram. A complete characterisation of each included study, including its intervention mechanism, technology, region, country, target population, and study design or quality, is provided in the supplementary online material.

### 5.2 Description of impact evaluations

This section presents summary information about included studies. Some of the earliest WASH trials were led by LMIC researchers, such as Khan's (1982) factorial study of handwashing and water treatment and storage in Bangladesh, the cross‐over trial of household water treatment by Kirchhoff et al. (1985) in Brazil, as well as RCTs of handwashing in Myanmar (Han & Hlaing, 1989) and a multiarm trial of filtration and hand‐washing in Guatemala (Universidad Rafael Landivar, 1995). The rest of the section discusses the evidence by intervention, outcome, participants and study design.

#### 5.2.1 Interventions

**Figure 9:**
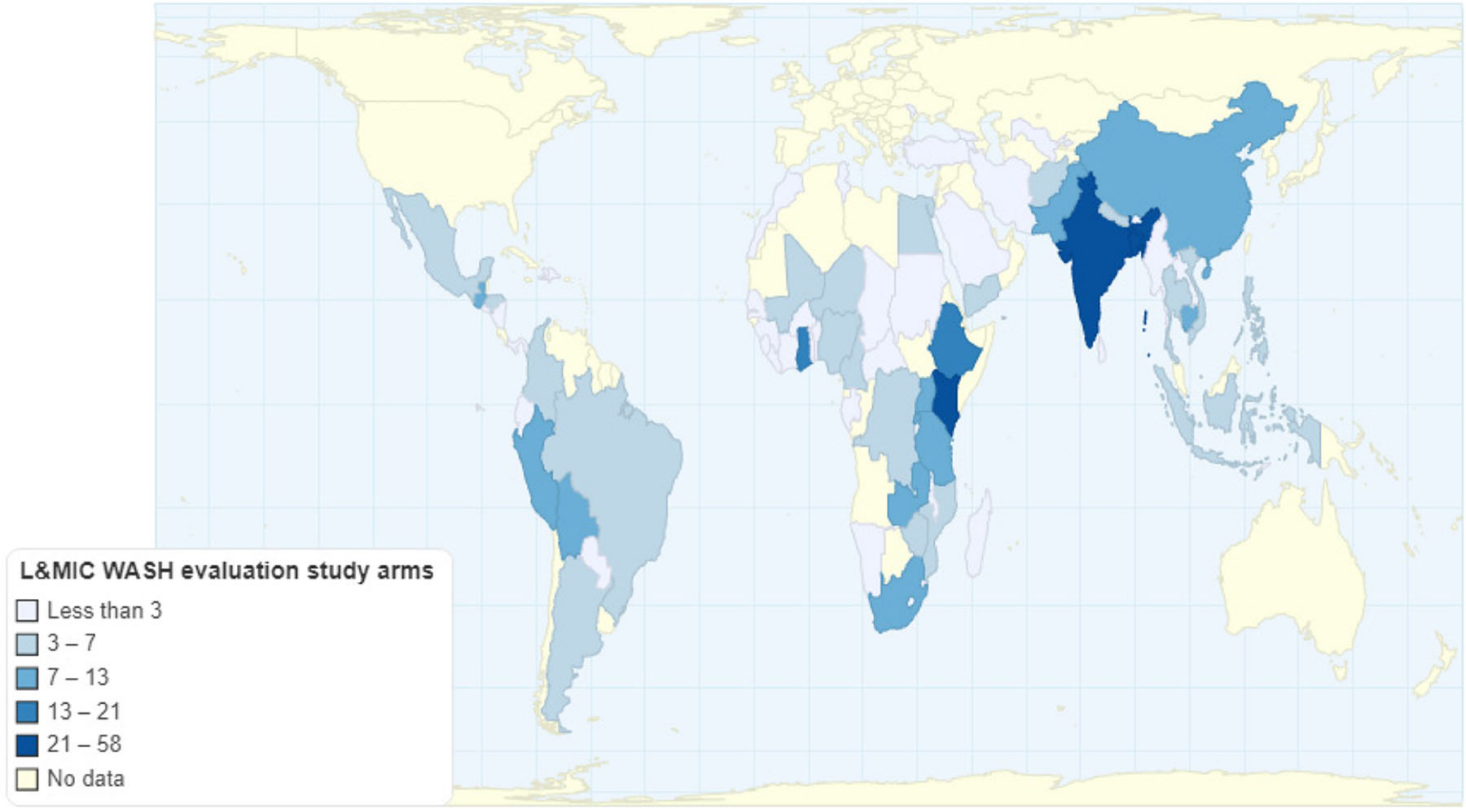
Map of WASH impact evaluation interventions in LMICs. *Source*: created using http://chartsbin.com/

**Figure 10:**
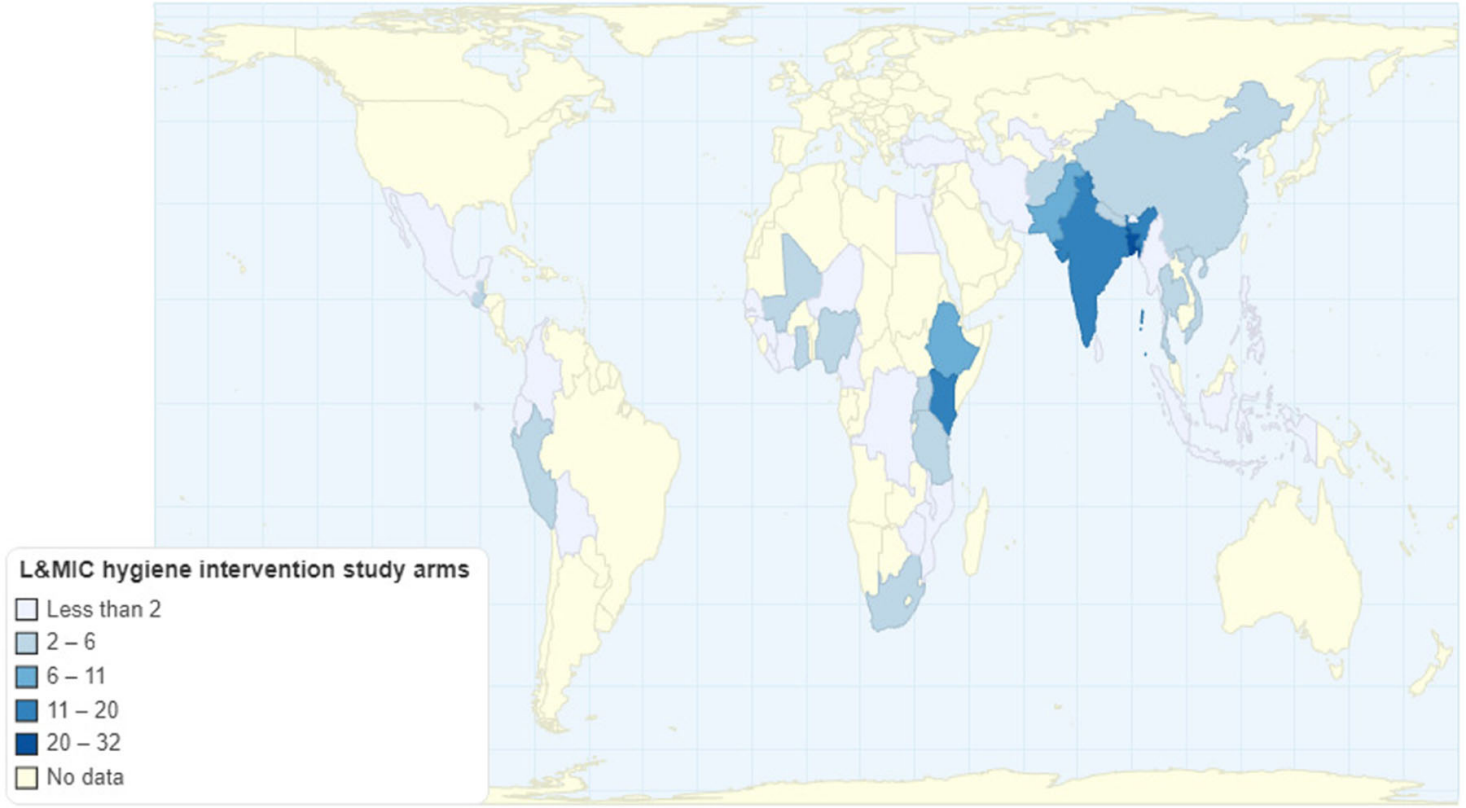
Map of hygiene impact evaluation interventions in LMICs. *Source*: created using http://chartsbin.com/. LMIC, low‐ and middle‐income country

Impact evaluations of WASH interventions have been conducted in 83 LMICs (Figure 9). There is a high concentration of studies in Bangladesh, Kenya and India, each having over 50 WASH intervention studies. In addition, Bolivia, Cambodia, Ethiopia, Ghana, Pakistan, Rwanda and Uganda each have 10 or more. Figure 10 also shows the global distribution of impact evaluations with hygiene studies (either solely or combined with other WASH interventions). The most hygiene studies have been done in Bangladesh, with 32 studies, while Ethiopia, India and Kenya all have 10 or more.

**Figure 11:**
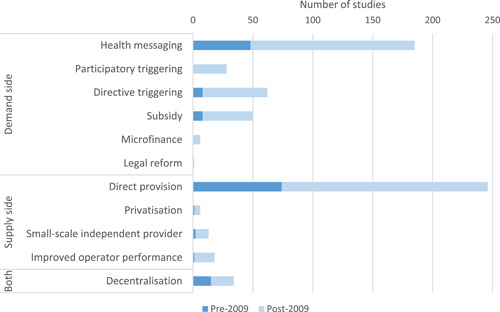
WASH intervention mechanisms by publication date

Over the past 15 years the focus of impact evaluation research has shifted from analysis of direct WASH technology provision by external authorities (e.g., governments and NGOs) to promotional interventions. There has been a particular increase in studies of BCC using psychosocial “triggering”, particularly of latrines and hand‐hygiene. In sanitation, this is most commonly CLTS. CLTS aims to ensure open defecation free (ODF) environments and increase the use of latrines by leveraging social cohesion to make collective behavioural changes, but can also include information campaigns focused on disgust or being a good parent, as well as incentives for community leadership to achieve ODF (Spears, 2013). Hygiene promotion includes approaches like “super‐Amma” (super‐Mum), which used disgust and pride to incentivise improved hand‐washing practices (Biran et al., 2014). Having said this, the traditional approaches of direct hardware provision (e.g., dispensing water filters) or health education (i.e., providing information about the consequences for health of not washing one's hands) remain common intervention mechanisms, even among new studies (Figure 11).^15^

**Figure 12:**
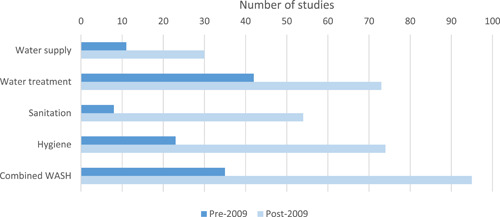
WASH technologies by publication date

Up until the end of the International Year of Sanitation, 2008, interventions to provide clean drinking water and hand hygiene had been the priority for intervention research. Almost 50% of studies had looked at how to improve water supply and quality. Household water treatment is still the most studied technology (around 30%). However, more studies (e.g., Brown et al., 2012; Klasen et al., 2012) including two randomised encouragement trials (Ben Yishay et al., 2017; Devoto et al., 2010), have broadened the evidence base on health impacts of water supply provision. The number studies of sanitation technology has also increased from 8 to 62, and there has been a similar increase in the numbers of studies examining hygiene (from 23 to 97). Figure 12 shows the change in the studies examining each category before and after 2008.

**Figure 13:**
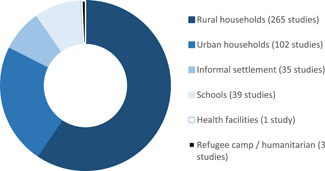
WASH technologies by place of use

The place of use further distinguishes technologies that are physically tied to a geographic location (Figure 13). Traditionally hardware has either been provided directly to a household or for communal use by a village, informal urban settlement (slum), or neighbourhood. There has, however, been increasing interest in the effect of providing WASH facilities at schools over the last 20 years. SDG target 4.A highlights the need for adequate WASH in schools. Correspondingly, most impact evaluations provide hardware directly to households. However, there are 39 studies on the communal provision of water supply (e.g., shared boreholes) and water quality technologies. Furthermore, although there were only two studies specifically examining WASH infrastructure in schools before 2008, there are now 39. The majority of these are combined water, sanitation, and hygiene technologies that act as a comprehensive overhaul for the school. Only one controlled impact evaluation was found of provision of handwashing supplies in healthcare facilities, and none were found in healthcare facilities for any other kind of WASH technology (Figure 15). It is worth noting the growing interest in WASH in healthcare facilities (WHO/UNICEF, 2019b) and the World Health Assembly, WASH in healthcare facilities is likely to be very high in people's priorities.

#### 5.2.2 Outcomes

The community of research producing WASH health impact evaluation studies is highly active. The total population included in WASH impact evaluations in LMICs included in this map is at least 5 million participants. Diarrhoeal disease, particularly carer‐reported diarrhoeal morbidity among children, remains the standard health impact measure used, and is by far the most commonly reported outcome (Figure 15). A systematic review from 2009 estimated 71 completed studies of WASH projects had been conducted measuring diarrhoeal morbidity (Waddington et al., 2009). The most recent systematic review of WASH and diarrhoea morbidity (Wolf et al., 2018) included 135 studies, and the evidence map presented here includes 186 studies measuring diarrhoea morbidity, 119 of which were in studies published after 2008. This map also indicates more than a million people have taken part in trials measuring, or had data collected on, diarrhoea morbidity.

Recognising the importance of WASH for controlling ARIs, the coverage of studies examining impacts on ARIs of hygiene promotion has also increased, with 35 studies measuring ARIs including 31 in studies published post‐2008, including large‐scale studies in in Vietnam (Chase & Do, 2017, 2012), Colombia (Correa et al., 2012), Bangladesh (Huda et al., 2012), Guatemala (Arnold et al., 2009) and Egypt (Talaat et al., 2011). Studies of the transmission of causative agents in unhygienic environments in LMICs have also measured the presence of respiratory infections like coronaviruses in stool samples (e.g., Esrey et al., 1987). However, given the importance of ARIs in the global burden of disease, due to pneumonia and influenza—as well as currently enhanced importance during the COVID‐19 pandemic—the total of number of participants in WASH studies of ARIs, at only only 125,000 in LMICs, remains very limited (Howard et al., 2020).

**Figure 14:**
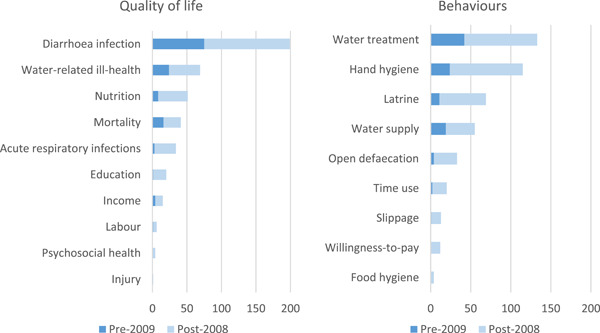
Number of impact evaluations by outcomes

In line with the other changes, there has been a shift in the commonly reported outcomes, including an increase in studies reporting behavioural outcomes since the early 2000s (Figure 14). This is an important shift as the principal argument used by proponents of alternative delivery mechanisms is that they are more effective at changing these behaviours and therefore improving lives (e.g., Kar & Chambers, 2008).

However, interventions fostering marginal improvements in personal WASH behaviour may not cause sufficient changes at community level to improve quality of life outcomes like child nutrition or diarrhoea mortality (Geruso & Spears, 2018). Hence, some behavioural studies also attempt to measure these outcomes too, since changing individual behaviours might not be enough to affect these “endpoint” outcomes. In other cases, repeated exposure to enteric infection may cause chronic malabsorption and food wastage (enteropathy), which one included study attempted to measure using a reference population of healthy individuals (Shiffman et al., 1978).

Although there has been an increase in studies in schools, it is still relatively few studies compared to other settings, given the importance of schools for transmission of infection in the public domain. Only three studies measured WASH in refugee camps or humanitarian emergency. Two of the studies were of water treatment technologies in refugee camps in Africa (Doocy & Burnham, 2006; Roberts et al., 2001). It is likely that water supplies are insufficient in refugee camp settings for studies of hygiene and insufficient space for latrines to be provided in sufficient density to impact on health. Very few studies have been done measuring time use and labour market outcomes, willingness‐to‐pay based on real purchase decisions, or studies measuring psychosocial health or injury (e.g., relating to attack or pedestrian road traffic accidents). Additionally, and critically, evidence of longer‐term behaviours, including slippage back to bad practices, is very limited.

The most commonly reported behaviours are handwashing, water treatment and handling, and latrine use. Many of the studies reporting hygiene behaviour include measures of personal food hygiene; nearly 50 studies specifically collected data on handwashing before food preparation, five reported on the microbial contamination of food or eating utensils, and 17 reported on other food hygiene outcomes, such as whether food was stored properly and dishes washed appropriately. It is important that hygiene studies examine food hygiene outcomes, given the importance of food in faecal‐oral disease transmission (Wagner & Lanoix, 1957). Studies collecting water supply behaviour outcomes included 40 interventions to reduce faecal contamination and six of chemical contamination due to arsenic in Bangladesh.

No studies estimating impacts on macro‐level outcomes such as private sector investment, employment generation or environmental impacts such as dumping or river water quality were found.^16^ The rest of this section discusses three important sector outcomes in detail: mortality, socioeconomic outcomes and sustainability and spillover effects.

##### Mortality

There is great policy interest in impacts of WASH on child mortality, which is weighted heavily in disability‐adjusted life year calculations (Cairncross & Valdmanis, 2006). Twenty seven evaluations have examined impacts of water provision and sanitation on child survival in LMICs. These include a number of Latin American studies conducted in Argentina (Galiani et al., 2005), Bolivia (Newman et al., 2002), Brazil (Rasella, 2013), Colombia (Granados & Sanchez, 2014), Ecuador (Galdo & Briceño, 2005), Honduras (Instituto‐Apoyo, 2000), Mexico (Venkataramani & Bhalotra, 2013) and Paraguay (World Bank, 1998b). A few studies have also been done in South Asia—Afghanistan (Meddings et al., 2004), Bangladesh (Luby et al., 2018), India (Clasen et al., 2014a,b; Spears, 2013), Nepal (Rhee et al., 2008), Pakistan (Bowen et al., 2012)—and others in Africa—Cote d'Ivoire (Messou et al., 1997a), Egypt (Abou‐Ali et al., 2009), Ethiopia (Gebre et al., 2011), Kenya (Crump et al., 2005; Null et al., 2018) and Mali (Pickering et al., 2015). Prospective studies examining child mortality are limited for ethical reasons required to measure death accurately, such as the need to withhold curative treatment such as oral rehydration or clinical treatment. However, some prospective studies reported diarrhoea mortality (Bowen et al., 2012; Luby et al., 2004; Messou et al., 1997a; Pickering et al., 2015) and a number of cluster‐RCTs reported all‐cause mortality in participant flow diagrams (e.g., Bowen et al., 2012; Clasen et al., 2014a; Luby et al., 2018; Null et al., 2018).

##### Education and economic impacts

The opportunity costs of children's time spent collecting water, or illness in childhood due to inadequate access to WASH, include education and economic impacts.

An increasing number of studies are reporting education outcomes, usually attendance and absenteeism, although enrolment has also been measured (e.g., UNICEF, 2011). Coverage includes Argentina (Ao, 2016), Bangladesh (Malek et al., 2016), Benin (Ruben & Kirk, 2011), China (Ban Ha et al., 2015), Egypt (Talaat et al., 2011), Ghana (Montgomery et al., 2012), India (Boisson et al., 2013; Khush et al., 2009; Nicholson et al., 2014; Orgill, 2017; Spears, 2013), Kenya (Caruso et al., 2014; Jack et al., 2015; Phillips‐Howard et al., 2016; Pickering et al., 2013), Mali (Alzua et al., 2015; Garn et al., 2017), Mexico (Venkataramani et al., 2015), Morocco (Devoto et al., 2012), Mozambique (UNICEF, 2011), Nepal (Oster & Thornton, 2011), Pakistan (Bowen et al., 2012; Rauniyar et al., 2009), Uganda (Montgomery et al., 2016), Yemen (Klasen et al., 2011, 2012), Zambia (Peletz et al., 2012). A number of these outcomes were collected in studies of WASH technologies for use in school, including Bhutan (UNICEF, 2014), Cambodia (Hunter et al., 2014), China (Bowen et al., 2007), Colombia (Overgaard et al., 2016), Kenya (Emory University, 2009; Freeman et al., 2012), Mali (Trinies et al., 2015), Niger (Boubacar Maïnassara & Tohon, 2014), Palestine (UNICEF, 2014) and Thailand (Pandejpong et al., 2012).

Only six studies measured labour market outcomes, in Georgia (Lokshin & Yemtsov, 2003), India (Khush et al., 2009), Mali (Alzua et al., 2015), Morocco (Devoto et al., 2012), Pakistan (Rauniyar et al., 2009), St Lucia (David et al., 2004) and Yemen (Klasen et al., 2012). Fourteen studies reported measures relating to income in Argentina (Galiani et al., 2009), Bangladesh (Hasan & Gerber, 2016; Khan, 1982), Burkina Faso (Briand & Lare‐Dondarini, 2017), Egypt (Abou‐Ali et al., 2009), Georgia (Lokshin & Yemtsov, 2003), India (Jeuland et al., 2015; Khush et al., 2009; Pattanayak et al., 2010), Moldova (Donnelly‐Hall & Ltd, 2004), Morocco (Devoto et al., 2012), Pakistan (Rauniyar et al., 2009), the Philippines (Aiga & Umenai, 2002), St Lucia (David et al., 2004) and Yemen (Dahl‐Østergaard et al., 2010). Chase (2002) estimated lost work time due to illness from an impact evaluation of the Armenian Social Fund's water supply improvements.

##### Sustainability, scalability, and spillovers

The importance of sustaining improved practices like use of hardware, defined here as being measured 12 or more months after implementation, and preventing slippage back to open defecation, and other bad practices, is well recognised. However, only 19 studies have measured sustainability of behaviours in Bangladesh, Bolivia, the Dominican Republic, Ethiopia, Ghana, Honduras, India, Mali, Pakistan, including six evaluations alone by Shordt and Cairncross (2004) in Bolivia, Ghana, India, Kenya, Nepal, Sri Lanka and Uganda. Even this is most commonly related to sustained handwashing practices rather than, for example, sustainability of latrine use or community ODF status, which is only measured in four completed latrine studies in Ethiopia and Ghana (Crocker et al., 2017), India (Duflo et al., 2015; Orgill, 2017) and Mali (Alzua et al., 2015), and in one study of community‐led total sanitation and hygiene (CLTSH) in Ethiopia (Delea et al., 2020).

Global reviews have suggested hygiene interventions produced the biggest and most reliable reductions in diarrhoeal illness, while economic appraisals have suggested hygiene is the most cost‐effective way of improving child health (Cairncross & Valdmanis, 2006). These estimates are mainly based on small‐scale projects. Impact evaluations of scaled‐up hygiene interventions have been conducted, for example in Bangladesh (Huda et al., 2012), Peru (Galiani et al., 2012) and Vietnam (Chase & Do, 2012). Hygiene information, education and BCC is a component of most, if not all, programmes aiming to scale‐up WASH services, but the extent to which hygiene components are adhered to in these programmes remains unclear (Jimenez et al., 2014). Hence, better understanding of the mechanisms through which these approaches work is needed.

Some studies measured water‐related health spillover effects from sanitation provision. Moraes et al. (2003) estimated effects on intestinal nematode infections at community and individual levels in a cross‐sectional evaluation of sewer connection in urban Brazil. Barreto et al. (2007, 2010) also examined impacts on diarrhoea and worm infection in the same city using a theory‐based cohort design. Finally, Duflo et al. (2015) measured spillover effects from decentralised community water supply and sanitation on diarrhoea and malaria prevalence.

#### 5.2.3 Gender and vulnerable populations

Existing impact evaluations cover rural, urban, and slum populations with the vast majority either targeting or including rural communities (295 studies), which are usually the most disadvantaged areas in terms of WASH service provision.

Research in WASH has long paid attention to vulnerable groups, such as poor women and girls, and their specific needs (Cairncross & Cliff, 1987; White et al., 1972). However, there is a long way to go before impact evaluation research and systematic reviews of these studies achieve the full integration of gender, and other equity concerns and become a consistently transformative sector (Interagency Gender Working Group [IGWG], 2018).

As discussed in the introduction, globally women and girls carry most of the burden of water collection, including time, calories spent, musculoskeletal injuries, having to use unsafe places to defecate, where water and sanitation services are not accessible, risking assault by people or attack by wild animals—so‐called “pests and perverts” (Campbell et al., 2015). Impacts on time use were not frequently studied in the context of intervention research in WASH. For example, a previous review of diarrhoea morbidity cited three studies which examined time savings from improved water supplies in India (Pattanayak et al., 2007), Nigeria (Blum et al., 1990) and China (Wang et al., 1989).^17^ Before undertaking searches for this map, only two nonintervention studies were known to have collected data on women's personal safety associated with sanitation improvements in Bhopal, India (Gosling et al., 2011) and Kampala, Uganda (Massey & SHARE, 2011).

In the last 10 years, new studies have been conducted that evaluated interventions and outcomes that disproportionately affect women and girls. This includes the 23 studies measuring various aspects of time savings and alternative uses of time due to water supply improvements, including in Argentina (Galiani et al., 2009), Bangladesh (Hasan & Gerber, 2016; Madajewicz et al., 2007), Benin (Ruben & Kirk, 2011), Burkina Faso (Briand & Lare‐Dondarini, 2017), Ghana (Arku, 2010; Okyere et al., 2017), Guinea (Ziegelhoefer, 2012), Honduras (Instituto Apoyo, 2000), India (Jeuland et al., 2015; Pattanayak et al., 2010; World Bank, 1998a), Kenya (Jack et al., 2015), Lesotho (Feachem et al., 1978), Morocco (Devoto et al., 2012), Mozambique (Cairncross & Cliff, 1987; UNICEF, 2011), Nigeria (Toonen et al., 2014), Pakistan (Rauniyar et al., 2009), Philippines (Aiga, 2002), St. Lucia (David et al., 2004), Yemen (Dahl‐Østergaard et al., 2010; Klasen et al., 2011). In addition, one study measured time use due to improved latrine coverage (Dickinson et al., 2015).

There are four completed impact evaluations measuring psychosocial health outcomes in India (Khush et al., 2009), Morocco (Devoto et al., 2012), Uganda (Montgomery et al., 2016), and safety and vulnerability in Brazil (Bobonis et al., 2017). Five more were identified as ongoing in 2018, two of which are now completed (Dreibelbis et al., 2019; Delea et al., 2020).

Women and adolescent girls also experience hardships where inadequate services constrain menstrual hygiene management. There are now also five studies of menstrual hygiene management in Ghana (Montgomery et al., 2012), Iran (Fakhri et al., 2012), Kenya (Phillips‐Howard et al., 2016), Nepal (Oster & Thornton, 2011) and Uganda (Montgomery et al., 2016) and two systematic reviews (Hennegan & Montgomery, 2016; Sumpter & Torondel, 2013). However, only a single study in Pakistan (Rauniyar et al., 2009) reported impacts from improved water provision on self‐reported drudgery, measured as incidence of muscle strain, blisters or backache. No study has measured musculoskeletal disorders arising from long‐term water carrying.

Gender analysis is rarely used as part of the framework for understanding programme effects in impact evaluations and systematic reviews. In fact, only a minority of studies included in the map (19% of impact evaluations and 20% of systematic reviews) reported any sex disaggregated outcomes. In impact evaluations, psychosocial health (43%), education and cognitive development (40%), open defecation (33%), and time use (26%) were some of the most commonly sex disaggregated outcomes. It is also notable that the map was not able to identify any controlled impact evaluations reporting sanitation use by nonbinary or transgender individuals.

There were no controlled studies either examining WASH interventions and technologies that targeted people living with a disability or, most strikingly, reporting on the success of standard WASH approaches in improving outcomes for these disadvantaged groups. This may be in part due to large sample size requirements necessary to measure outcomes among these populations, although there are presumably places where studies could be done relatively easily (e.g., special schools). For instance, an uncontrolled cohort study was conducted of hygiene in child day care facilities in Brazil (Barros et al., 1999), and intervention studies have been conducted of day care in high income countries (e.g., Black et al., 1981; see also Wolf et al., 2018).

The findings are also sparse when looking at other vulnerable populations. There is one impact evaluation (Abebe et al., 2014) and two systematic reviews (Peletz et al., 2013; Yates et al., 2015) that examined people living with HIV, who often have different social constraints and medical needs. There are two impact evaluations (Doocy & Burnham, 2006; Roberts et al., 2001) that specifically look at the needs of those living in refugee camps, and one impact evaluation (Chase et al., 2002) and two systematic reviews (Brown et al., 2012; Ramesh et al., 2015) looking at those living through, or in the aftermath of, a humanitarian crises.

#### 5.2.4 Study design

Well over half of impact evaluations (comprising over 250 trial arms) used randomised assignment (RCTs) (Table 7), indicating the extent of support in academic and research funding communities for this study method. Some RCTs have taken full advantage of the power of the methodology by conducting comparative designs with prospective randomised assignment to alternate intervention mechanisms. Guiteras, Levinsohn, et al. (2015) provided an example in Bangladesh comparing different groups that were provided community sanitation promotion (CLTS) or latrine subsidies, on rates of open defaecation. Other recent examples of comparative designs include Luby et al. (2018) in Bangladesh and Null et al. (2018) in Kenya which compared WASH interventions individually and in combination.

**Table 7:** Ethical review in WASH impact evaluations (percent)

	Total	Environmental health	Social science
Passed any IRB	43	55	22
o/w passed IRB in country of data collection	37	47	16
No IRB was consulted	6	5	11
Unclear/not stated	49	39	67

*Note*: may not sum to 100% due to rounding errors.

Typical nonrandomised study designs include cross‐section studies with statistical matching (e.g., Abou‐Ali et al., 2009), group level panel data studies analysed at aggregated administrative levels (e.g., Galiani et al., 2005), individual‐level panel data studies (e.g., Galiani et al., 2009), pseudo‐panels with repeated cross‐section from the same clusters (Galdo & Briceno, 2005), case‐control studies (e.g., Meddings et al., 2004), and nonrandomised pipeline studies (e.g., Cairncross & Cliff, 1987). In NRS using matching, the matching was usually done using statistical methods, although a few used “naïve” matching (e.g., World Bank, 1998).

**Figure 15:**
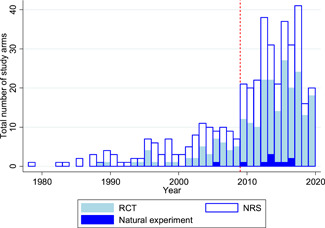
WASH impact evaluations by study design. Dotted line shows the end of the International Year of Sanitation (2008). The apparent decline in production of studies post‐2018 reflects the limited searches done in this map after 2018. NRS, nonrandomised studies; RCT, randomised controlled trial

A few NRS (11) have taken advantage of existing data to conduct rigorous, and potentially highly cost‐effective, evaluations with selection on unobservables, here called natural experiments (Ao, 2016; Calzada & Iranzo, 2013; Galiani et al., 2005; Galiani et al., 2009; Granados & Sanchez, 2014; Kosec, 2013; Spears, 2013; Tiwari et al., 2017; Ziegelhoefer, 2012). Methods used to analyse data in natural experimental frameworks include RDDs (Ziegelhoefer, 2012), ITS (e.g., Duflo et al., 2015) and panel data regression (e.g., Galiani et al., 2005). Figure 15 presents the evolution of study designs over time, indicating the marked increase in studies produced after the end of the International Year of Sanitation, from 2009 onwards, of completed and registered studies.^18^ It is striking, given the large numbers of data sets in existence, both from field trials, administrative sources and household surveys, how few studies have taken advantage of natural experiments to conduct rigorous evaluations of WASH interventions with selection on unobservables.

Natural experiments that applied statistical methods of correction for unobservable confounding to existing surveys, therefore, remain an under‐utilised methodological approach in the WASH sector, given the large numbers of existing household survey datasets available that contain questions on access to WASH services which, for example, could be analysed as pseudo‐panels using double‐difference methods. It is also important to note that there continues to be a great number of uncontrolled studies that simply measure outcomes before and after the intervention. Most of these studies were excluded from the map as they are not usually able to attribute changes to the intervention, the exception being for the immediate outcomes of time savings due to provision of a new water supply or sanitation source (e.g., Cairncross & Cliff, 1987).

### 5.3 Description of systematic reviews

**Figure 16:**
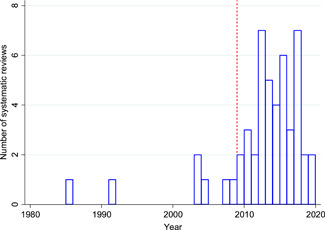
Production of WASH systematic reviews of evidence in LMICs. Dotted line shows the end of the International Year of Sanitation (2008). The apparent decline in production of studies post‐2018 reflects the limited searches done in this map after 2018

The classic systematic review, produced when systematic reviews had not yet been properly defined, was on the control of diarrhoeal disease in young children commissioned by the WHO Diarrhoeal Diseases Control Programme (Esrey et al., 1985). Esrey et al. (1991) produced a second review that focused on effects of WASH on water‐related disease including diarrhoea, helminth infection and schistosomiasis, and trachoma. Since then, an estimated 43 completed systematic reviews have synthesised the findings of WASH interventions and exposures (Figure 16), and two are ongoing (Piper et al., 2017; Waddington & Cairncross, 2021).^19^

Systematic reviews of WASH studies include evidence from all global regions and cover a breadth of WASH technologies, outcomes and, increasingly, intervention mechanisms. As impact evaluations make up the underlying body of research, systematic reviews also predominantly focus on health outcomes, particularly diarrhoea and enteric infections. The earliest reviews of enteric infections associated with water and sanitation provision including diarrhoea (Esrey et al., 1985) and water‐related infections (Esrey et al., 1991), were explicitly restricted to published literature. Even so, Esrey et al. (1991) found large numbers of eligible studies (144 studies), partly due to comprehensive inclusion of outcome categories (diarrhoea, ascariasis, Guinea worm infection, hookworm infection, schistosomiasis and trachoma), and partly due to inclusivity by study design. A large subsequent systematic review literature examined the effects of WASH technologies on diarrhoeal health outcomes (Clasen et al., 2015; Curtis & Cairncross, 2003; Fewtrell & Colford, 2004; Gundry et al., 2004; Ejemot et al., 2007; Cairncross et al., 2010; Clasen et al., 2010; Ejemot‐Nwadiaro et al., 2015; Freeman et al., 2014; Norman et al., 2010; Waddington et al., 2009; Wolf et al., 2018). An increasing number of reviews measured other commonly evaluated outcomes, including “neglected tropical diseases” such as helminth infections (Esrey et al., 1991; Freeman et al., 2017; Strunz et al., 2014; Ziegelbauer et al., 2012), and trachoma (Ejere et al., 2015; Esrey et al., 1991; Freeman et al., 2017; Rabiu et al., 2012; Stocks et al., 2014). Other reviews focussed on nutritional outcomes (Dangour et al., 2013) or incorporated them (Freeman et al., 2017). Reviews increasingly focus on behavioural and socioeconomic outcomes. For example, Annamalai et al. (2016) reported time savings and Null et al. (2012) focussed on willingness‐to‐pay based on real purchase decisions.

Most of the systematic reviews draw clear boundaries for eligible technologies and outcomes and then include, and mix together, the different mechanisms by which they are provided. However, this is changing with the focus of recent systematic reviews by Annamalai et al. (2016) on top‐down versus bottom‐up approaches in water and sanitation, and De Buck et al. (2017) and Venkataramanan et al. (2018) on sanitation and hygiene BCC and CLTS respectively. All of these reviews synthesised impact evaluations and qualitative studies in mixed methods reviews, arguably providing more useful information for policy and programmes.

Updates of reviews have also become common as the evidence base expands. Systematic review updates have been done for the Cochrane reviews of household water treatment (Clasen et al., 2015) and hand hygiene (Ejemot‐Nwadiaro et al., 2015). The review on WASH and diarrhoea (Esrey et al., 1985) has now been updated at least five times (Cairncross et al., 2010; Esrey et al., 1991; Fewtrell et al., 2005; Waddington et al., 2009; Wolf et al., 2014, 2018). The minimum criteria for updating a review is to update the searches for studies published more recently. However, updates can usefully incorporate updates in other areas, such as the review's scope (e.g., additional outcomes or subgroups), engagement (e.g., more comprehensive stakeholder consultation) and quality (e.g., methodological improvements, such as more comprehensive risk of bias assessment) (Waddington et al., 2018). For example, reviews of health impacts are incorporating analysis of participant adherence (Clasen et al., 2015).

### 5.4 Critical appraisal

#### 5.4.1 Impact evaluations

Blum and Feachem (1983) conducted a review of six areas where WASH impact evaluation designs were suboptimal: use of a control group, adjustment for confounding, definition of the outcome, length of recall, analysis of use, and group sample size, which they referred to as “one‐to‐one” comparison.^20^ Data were collected on these areas for impact evaluations contained in the evidence map. The review suggests these points have been incorporated into common practice by WASH researchers. Thus, all studies used control or comparison groups who received no or a different intervention, with the exceptions of Duflo et al. (2015) who used interrupted time series (ITS) to measure infectious diseases following household water connections, and Arku (2010) who measured time use by participant recall before and after installation of improved community water supply. As noted above, before‐after design is the preferred approach to measuring immediate outcomes where there is no risk of confounding (Victora et al., 2004).

Studies addressed confounding either through random assignment, group or individual level matching on observables before analysis, or directly in adjusted analysis. For example, nearly all RCTs used centrally administered randomisation, although there was the occasional exception where a study used quasi‐randomisation through alternation (Montgomery et al., 2016). Some studies used randomisation over very small samples, such as Stone and Ndagijimana (2018) who randomised across two districts in Rwanda.

Outcomes were nearly always clearly defined for diarrhoea (95% of cases) usually being the WHO definition of “three or more loose stools in a 24 h period”, and where diarrhoea incidence was recorded “three intervening diarrhoea‐free days” were required to define a new episode (Bacqui et al., 1991). Outcomes data collection, whether on behaviour or quality of life, remains heavily reliant on self‐reporting, which is thought to be highly susceptible to biases, including social desirability, also called “courtesy bias”. A different form of courtesy bias may also manifest in reported data due to participant fatigue when repeated follow‐up data are collected, also called the “Bugger‐Off Effect” (Clasen, 2013; see also Schmidt & Cairncross, 2009). “Survey effects”, or measurement as treatment, have also been empirically observed in repeated follow up studies (Schmidt et al., 2011; Zwane et al., 2011). Indeed, following the publication of papers on bias arising due to repeated measurement (Schmidt et al., 2011), the mean number of survey rounds for health impact studies measuring self‐reported diarrhoea fell from 23 to 7, and the median fell from 12 to 3.

Other factors that may cause bias in reported data have also improved. For example, for self‐reported diarrhoeal disease, only a minority of studies used recall periods longer than two weeks (Elbers et al., 2012; Galiani et al., 2009; Iijima et al., 2001; Pradhan & Rawlings, 2002; Walker, 1999). Studies measuring respiratory infection by self‐report used recall periods of at most seven days. Some studies used observation, hospital records, and medical samples to improve the objectivity of the measures (e.g., Khan, 1987), although this remains rare.

Study sample sizes have also increased alongside greater availability of research resources. The median number of clusters is 21 (and the mean 79), whether cluster is defined as community, village, informal settlement, neighbourhood, municipality, school or health care facility. For example, until 2008 the median number of clusters was only 10 (the mean was 49), whereas post‐2008 it was 31 (mean of 92); less than a quarter of studies published from 2009 onwards have cluster sample sizes of less than ten.

**Figure 17:**
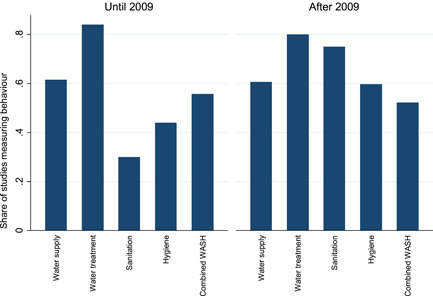
Measurement of behaviour change

It is necessary to go beyond “bare bones” in impact evaluations (Mark & Lenz‐Watson, 2011) by collecting data to answer relevant questions about implementation and causal mechanisms, not just on effects. As noted above, the collection of data on access to and use of facilities (behaviour change) is well‐established (WHO, 1983). Analysis of this causal pathway was done from the earliest WASH health impact evaluations, including trials of hygiene (e.g., Torun, 1983) and water treatment technology (e.g., Kirchhoff et al., 1985). Over half of studies collected data on behavioural outcomes, a share that increased over time particularly for evaluations of sanitation and hygiene technologies (Figure 17).

Follow‐up length varied by outcome. Studies of direct provision and health messaging, those measuring diarrhoea and ARIs or water treatment and hygiene behaviours, were conducted over relatively shorter periods, with a median number of 12 months each. In contrast, studies of intervention mechanisms such as decentralisation (e.g., CDD, median 24 months) or those measuring socioeconomic outcomes, which may take longer to materialise as they are further down the causal pathway than behaviours and health, tended to be conducted of longer follow‐ups (median of 19 months for education outcomes, 30 months for income, and 48 months for labour market outcomes).

Researchers and funders appear to have been sensitive to calls for greater examination of sustainability of interventions and outcomes (e.g., Waddington et al., 2009). For example, evaluations of CLTS, all of which were published since 2012, include studies measuring open defaecation several years after implementation—4 years in the case of Adank et al., 2016, and 10 years for Orgill (2017), which also measured education outcomes. The increased value in longer follow‐up periods is well‐recognised as a necessary check on slippage (Adank et al., 2016).

**Figure 18:**
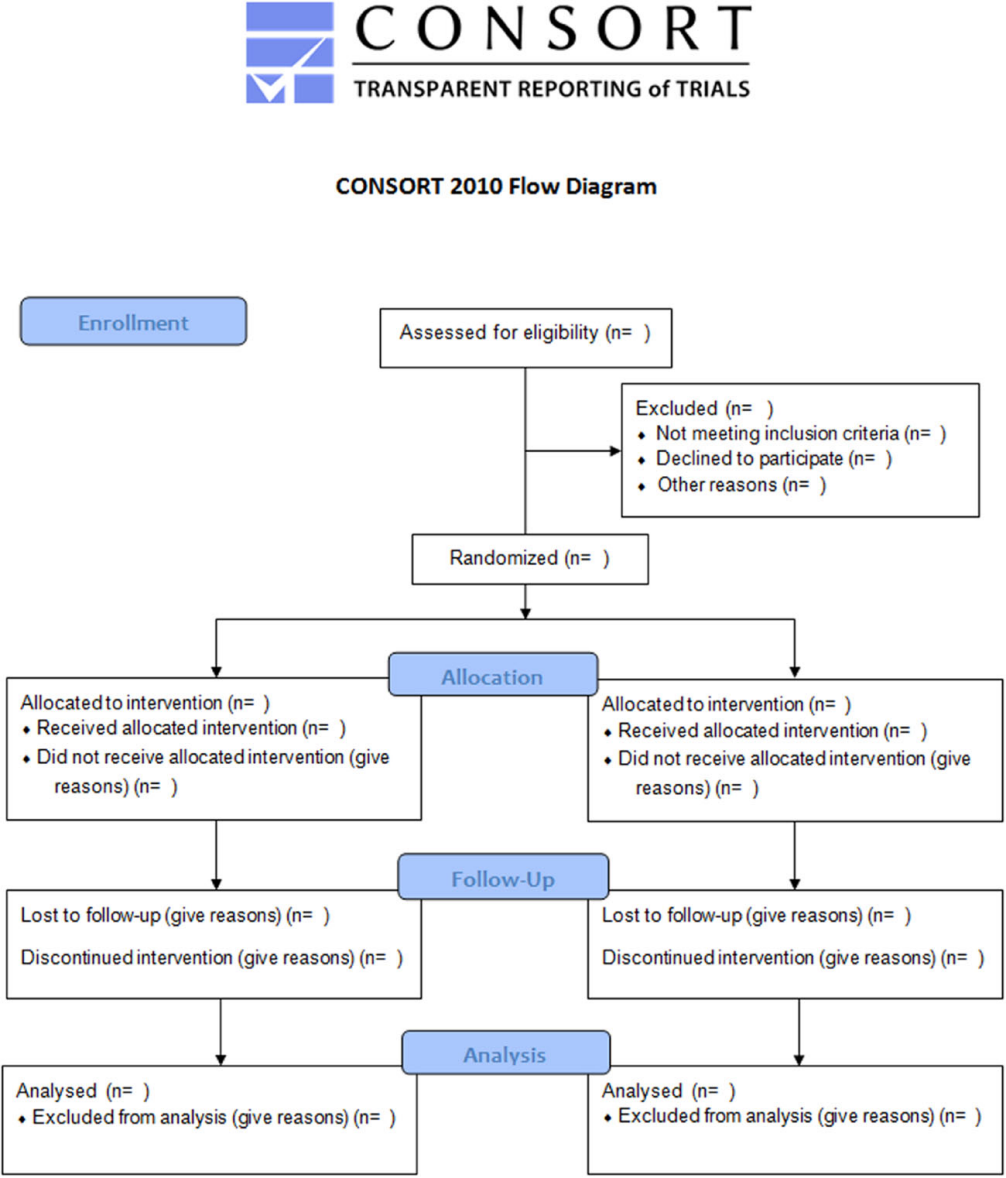
The CONSORT flow diagram. *Source*: http://www.consort-statement.org/consort-statement/flow-diagram

However, current standards for reporting of prospective studies leave much room for improvement. The Consolidated Standards of Reporting Trials (CONSORT) standards (Moher et al., 1998) provides basic standards for reporting participant flow in trials, from recruitment to allocation and follow up (Figure 18). Information should be provided for each study group at each stage in the trial, in order for analysis of bias, particularly risk of selection bias.

**Figure 19:**
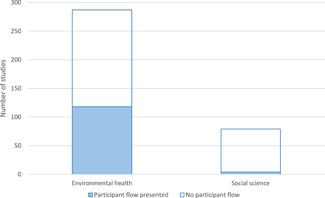
Participant flow diagrams by academic discipline

However, the basic requirements of reporting participant flow adherence in field trials according to CONSORT standards are frequently unmet. If the reporting in environmental health is substandard, with less than 50% of studies presenting participant flows, the reporting in social sciences may go as far as being deliberately misleading (Figure 19). Only two out of 54 prospective studies in social science presented a participant flow diagram or the data from which it could be fully reconstructed (Beath & Enikolopov, 2013; Guiteras, Jannat, et al., 2015). Some newer studies by social scientists are starting to exhibit flow diagrams for the full trial period, at the cluster level (e.g., Armand et al., 2020).

Data were also collected on ethical review reported in WASH impact evaluations (Table 7). Again, while standards in environmental health, where over half of studies that would need ethical review, could be improved, the standards in social science leave much to be desired. Only 22% transparently indicated an IRB had approved the evaluation, and even fewer (16%) had done IRB in‐country; nothing was indicated about ethical review in 67% of cases. In over 10%, no ethical review procedures were reported. Thus, no study published by a United Nations (UN) body, whether the World Bank, a regional development bank or other UN organisation indicated that an institutional review process was undertaken before study implementation.

It is possible that programme evaluations, which are the studies conducted by UN organisations, are thought not to require ethical approval, as they are being rolled out anyway. For example, Semenza et al. (1998) indicated that “IRB review was not required because the study did not fall under the human subjects regulations” (p. 941) as it was a programme evaluation. That evaluation included a component where participants were randomised to receive chlorine and a safe storage device. In the case of prospective evaluations done by UN bodies including the development banks, there may be ethical issues relating to withholding treatment from control communities, or the ethical standards around, for example, compensating participants for their time (White, 2013), and possibly by offering health treatment to the severely ill, such as oral rehydration salts and health check‐ups for severe diarrhoea.

#### 5.4.2 Systematic reviews

The included systematic reviews were critically appraised using the tool developed by Jimenez et al. (2018). Systematic reviews were critically appraised according to the methods of search, analysis, and synthesis, as well as the types of evaluation questions answered and the incorporation of mixed‐methods evidence. Table 8 presents a summary of the quality assessments for each included review.

**Table 8:** Summary of appraisals of WASH systematic reviews, grouped by review topic

Topic	Review	Search date	Confidence in systematic review findings about effects	Type of review
Water, sanitation, and hygiene technology provision	Esrey et al. (1991)	1989	As the first comprehensive literature review on WASH and enteric infection, the review was not conducted to present‐day standards, and has *major limitations*. It excluded unpublished findings, inadequately reported searches and inclusion decisions, inadequately reported critical appraisal methods, and used inappropriate methods to pool findings, reporting median effects (rather than statistical meta‐analysis)	Traditional systematic review drawing solely on quantitative evidence of effects on health outcomes without explicit model of behaviour
	Fewtrell and Colford (2004); Fewtrell, and Kaufmann, Kay, et al. (2005)	June 2003	The review of diarrhoeal morbidity was well designed, importantly taking into account baseline WASH conditions, but has *major limitations* which affect the confidence in the findings. Independent screening and data extraction were not conducted by at least two reviewers. The study designs eligible for inclusion were broad, leading to inclusion of studies with high risk of bias in attributing outcomes to the intervention. Although meta‐analysis was carried out with and without poor‐quality studies, studies with high risk of bias still remained in both analyses. Finally, unit of analysis errors were not into account in calculation of standard errors	Systematic review and meta‐analysis using explicit logic model and drawing exclusively on quantitative evidence for endpoint health outcomes
	Waddington et al. (2009)*	February 2009	The review, incorporating diarrhoea and secondary time‐use outcomes, was based on a comprehensive search with clear inclusion criteria and use of mixed methods to analyse findings. However, the review has *limitations*. Two reviewers critically appraised included studies but did not independently calculate effect sizes. Given the range of quasi‐experimental studies included, the methods used to assess risk of bias were not sufficiently comprehensive. In addition, unit of analysis errors were referred to but not addressed	Theory‐based systematic review and meta‐analysis using explicit model of behaviour change and drawing systematically on quantitative evidence and on qualitative evidence contained in quantitative studies along causal pathway
	Cairncross et al. (2010)	2005 and 2008	The review was based on relatively comprehensive searches for literature and appropriate methods to reduce risk of bias in study selection and analysis. However, the review has *limitations*. The search for studies on hand washing was limited to English, risking language bias, and unit of analysis issues for clustered randomised trials were not addressed. It is not clear whether dependencies at study or review level were addressed in analysis. Limitations of the review and the underlying evidence base were acknowledged, and conclusions carefully presented	Traditional systematic review and meta‐analysis drawing solely on quantitative evidence of effects without explicit model of behaviour
	Dangour et al. (2013)	June 2012	The systematic review of WASH and nutrition outcomes was conducted to include English and Chinese language studies, and the results were presented using statistical meta‐analysis to synthesise effects on endpoint outcomes. The *minor limitations* of the review are that it does not report impacts on behavioural outcomes.	Systematic review and meta‐analysis using explicit model of behaviour change and drawing exclusively on quantitative evidence for endpoint outcomes
	Strunz et al. (2014)	October 2013	The systematic review of WASH and soil transmitted helminth infections has *major limitations*. The review did not undertake a sufficiently comprehensive by including reference harvesting (checking the reference lists of included studies for eligible reports), or contact authors or experts to identify missing papers. The review does not address unit of analysis errors, as clustered trials are included but clustering is not taken into account in the analysis.	Systematic review and meta‐analysis drawing solely on quantitative evidence of effects on health outcomes without explicit model of behaviour, although authors differentiate access and use in analysing effects
	Darvesh et al. (2017)	September 2016	The systematic review has *major limitations*. A reasonably comprehensive search for evidence was not conducted, as searches and inclusion were limited to published literature and English language articles, so the study was liable for publication and language bias. No table or summary of the risk of bias assessment for each included study was given, for each criterion, although overall ratings of weight of evidence using GRADE were provided. There was no table or summary of the characteristics of the participants, interventions, and outcomes of the included studies, and heterogeneity in findings was explored at the intervention level only. Unit of analysis errors were not addressed, although there were several cluster randomised trials included	Traditional systematic review and meta‐analysis drawing solely on quantitative evidence of effects without explicit model of behaviour
	Wolf et al. (2014, 2018)	February 2016	The systematic review of diarrhoea morbidity used network meta‐analysis to address confounding by baseline WASH and analysed subgroups accordingly. The study also used a Monte Carlo approach to adjust for bias due to the lack of blinding in water treatment and hygiene studies. However, it has *major limitations*. Searches for grey literature were not undertaken, or independent double screening or data extraction. The potential for unit of analysis errors was not fully addressed. While the review used appropriate critical appraisal questions, a numerical scoring system to rate bias was used, which is recognised to confound different sources of bias (Juni et al., 2001). The limitations of the review are acknowledged, but there is not a sufficient justification for not conducting a grey literature search	Systematic review and meta‐analysis using explicit logic model and drawing exclusively on quantitative evidence for endpoint outcomes
Hygiene provision and promotion	Curtis and Cairncross (2003)	2002	The systematic review of diarrhoea outcomes used a reasonably comprehensive search for literature, assessed the risk of bias appropriately, and analysed studies on the basis of quality and study design, attenuating biases in included studies. However, the systematic review has *limitations*. The search included studies published in English, and did not sufficiently comprehensively search for grey literature. The authors reported little information on the process of screening and data extraction, including whether two authors independently screened studies and extracted data	Traditional systematic review and meta‐analysis drawing solely on quantitative evidence of effects on health outcomes without explicit model of behaviour
	Aiello et al. (2008)	May 2007	The systematic review, examining effectiveness of hygiene on diarrhoea and ARIs, included appropriate evidence and was reported transparently, including relevant subgroup analyses. However, the review has *major limitations*. It was restricted to findings from the published literature, and so is liable to publication bias, and did not assess the risk of bias of included studies systematically. The authors carried out sensitivity analysis to assess the effect of including nonrandomised and nonblinded studies and cluster‐assigned studies with a unit of analysis error, therefore partially accounting for potential biases in attributing outcomes to the intervention	Traditional systematic review and meta‐analysis drawing solely on quantitative evidence of effects on health outcomes without explicit model of behaviour
	Freeman et al. (2014)	August 2013	The systematic review has *major limitations*. The authors did not search grey literature comprehensively, provide a table or summary of the assessment of each included study. The meta‐analysis was not reported transparently, and no exploration or analysis done of heterogeneity. The approach taken to adjust studies for nonblinding was also not transparently reported	Traditional systematic review drawing solely on quantitative evidence of effects on health outcomes without explicit model of behaviour
	Ejemot‐Nwadiaro et al. (2015)*	May 2015	The systematic review used robust and transparent methods to identify and include studies of diarrhoea impacts. It used appropriate methods for assessing quality of included studies and for undertaking synthesis of findings using meta‐analysis. It has *minor limitations*	Theory‐based systematic review and meta‐analysis incorporating behaviour change and drawing exclusively on quantitative evidence along causal pathway (intermediate and endpoint outcomes)
	Watson et al. (2017)*	July 2016	The systematic review provided a useful discussion of potential factors affecting health and behaviour change outcomes. However, the review has *major limitations*. A reasonably comprehensive search for evidence was not conducted, and only included published, peer‐reviewed studies in English, were included, so the review is liable to publication bias and language bias. Independent data extraction by at least two reviewers was not specified, and findings were analysed using vote counting rather than narrative synthesis of effect sizes	Systematic review drawing exclusively on quantitative evidence along causal pathway for intermediate and endpoint outcomes
	Rabiu et al. (2012)*	September 2011	The systematic review, assessing hygiene and trachoma, used a reasonably comprehensive search strategy, clear inclusion criteria and clear reporting of screening and data extraction procedures, as well as appropriate methods of risk of bias assessment and data analysis. The review has *minor limitations*. The grey literature search may not be comprehensive, and unit of analysis error was not addressed	Theory‐based systematic review drawing exclusively on quantitative evidence along causal pathway including behaviour change and endpoint outcome
	Ejere et al. (2015)	January 2015	The systematic review, assessing the effect of hygiene promotion on trachoma. It used appropriate methods of search, critical appraisal and reporting, and refrained from overstating conclusions. It has minor limitations	Traditional systematic review drawing solely on quantitative evidence of effects on health outcomes without explicit model of behaviour
Water provision	Gundry et al. (2004)*	December 2000	The systematic review of diarrhoea and water treatment behaviour was based on a relatively comprehensive search for published literature, reporting criteria for study inclusion and information about the included studies, and reporting effects using meta‐analysis. However, the review has *limitations*. The search for unpublished literature was not exhaustive, and the review did not include information on the screening or data extraction process, so it is unclear whether risks were reduced from independent screening and data extraction by more than one reviewer	Systematic review and meta‐analysis drawing exclusively on quantitative evidence along causal pathway for intermediate outcomes and health impacts
	Arnold and Colford (2007)*	March 2006	The systematic review of diarrhoea and water treatment behaviour used appropriate methods to reduce biases in terms of clear inclusion criteria, reasonably comprehensive search and analysis of data incorporated. However, the review has *limitations*. It was limited to studies published in peer reviewed journals, so the review is liable to publication bias. There were no restrictions on study design other than that studies include comparison groups, and the type of evidence used is in principle appropriate for answering the review question. However, the assessments of bias in the included studies were limited.	Systematic review and meta‐analysis drawing exclusively on quantitative evidence along causal pathway. measuring intermediate outcomes and health impact
	Hunter (2009)	February 2009	The meta‐analysis analysed the reasons behind differences in effectiveness, using meta‐regression analysis. Since it was based on the findings of other systematic reviews and meta‐analyses, it did not report information on searches and characteristics of the included studies, and was carried out by one researcher. The *major limitations* of the study were that it did not assess the risk of bias in included studies, apart from whether they used appropriate methods of blinding, it was not based on a study protocol, so it is not clear whether results‐based choices were made in analysis. Correlated study arms were included in the same analyses, which may falsely inflate statistical precision. The study used a Monte Carlo approach to adjust for bias due to the lack of blinding in the majority of studies	Meta‐regression analysis drawing exclusively on quantitative evidence along causal pathway (behaviour change and health outcomes)
	Clasen et al. (2006, 2007b, 2015)*	November 2014	The review used transparent and robust methods to search for and include studies and appropriate methods for assessing the risk of bias of included studies and synthesis. Extensive subgroup and sensitivity analyses were done to explore potential reasons for differences in findings in studies. The *limitations* were inclusion of correlated study arms in the same meta‐analyses and lack of reference to unit of analysis issues at the participant level (although the review did address unit of analysis at the study level)	Systematic review drawing solely on quantitative evidence of effects on health outcomes without explicit model of behaviour, correlating effectiveness with behaviour data collected in the included studies
Sanitation provision	Clasen et al. (2010)	Unclear	This systematic review used clear and transparent methods of search, inclusion and methods of analysis of studies. Important innovations of the review were the search in Chinese language databases and the assessment of unit of analysis errors contained in the included studies. The review uses transparent criteria to assess the quality of included studies and assesses (although does not correct) unit of analysis errors. It has a *limitation* due to the exclusion of at least one rigorous quasi‐experiment	Traditional systematic review and meta‐analysis drawing solely on quantitative evidence of effects on health outcomes without explicit model of behaviour
	Norman et al. (2010)	February 2010	The systematic review used clear inclusion criteria for studies of diarrhoea and enteric infection (including helminths), a targeted search strategy, and standardised methods for extracting and synthesising data from the included studies, including an explicit exploration of observed heterogeneity. The authors attempted to explain the heterogeneity by subgroup analysis. However, the review has *major limitations*. The risk of bias approach was insufficiently transparent, and unit of analysis errors in the reporting were not addressed	Traditional systematic review and meta‐analysis drawing solely on quantitative evidence of effects on health outcomes without explicit model of behaviour
	Ziegelbauer et al. (2012)	December 2010	The systematic review of sanitation and helminth infections has *major limitations*. The search for grey literature was not done, nor were relevant subject experts contacted to identify unpublished manuscripts. The risk of bias approach conflates using a scoring system which is not sufficiently transparent. Further, the authors do not present or analyse studies by risk of bias rating. Finally, the meta‐analysis does not take effect size dependency into account. Some limitations are acknowledged in the review	Systematic review and meta‐analysis drawing solely on quantitative evidence of effects on health outcomes without explicit model of behaviour, although authors do differentiate access and use in analysing associations with health outcomes
	Heijnen et al. (2014)	September 2013	The systematic review of diarrhoea and faeces‐related infection was done using a thorough search for studies and presented both quantitative synthesis and narrative synthesis when appropriate. The review has *limitations*. No sensitivity analyses for meaningful sources of bias in the included studies were done, and heterogeneity observed was not accounted for. Unit of analysis errors were possible in included studies but not corrected. The authors offered a thorough listing of limitations in the primary studies, but could have been clearer about the limitations of the meta‐analysis itself	Traditional systematic review and meta‐analysis drawing solely on quantitative associational evidence on health outcomes without explicit model of behaviour
	Stocks et al. (2014)	October 2013	The systematic review assessed sanitation and trachoma. However, the systematic review has *major limitations*. It is not clear whether grey literature was eligible or reasonably comprehensive searches done for unpublished work. Although the quality of individual studies was evaluated using an approach based on GRADE, appropriate criteria were not used to assess included studies for risk of bias, or to assess the weight of evidence overall. The review did not provide a table or summary of the results of all included studies, only those included in meta‐analyses. The review did not address unit of analysis errors, as intra‐cluster correlation coefficients were not taken into account. Finally, the review reported high rates of heterogeneity in several meta‐analyses but did not explore heterogeneity or likely explanatory factors	Traditional systematic review and meta‐analysis drawing solely on quantitative evidence of effects on health outcomes without explicit model of behaviour
	Freeman et al. (2017)	December 2015	The systematic review comprehensively assessed outcomes arising from sanitation provision, including diarrhoea, helminths and trachoma. However, there are *major limitations*. There was no search for grey literature databases. Critical appraisal was done of experimental studies only, rather than all the included studies, and there was no table or summary of the assessment of each included study for each criterion for quality and risk of bias. Unit of analysis errors were not mentioned or taken into account in the analysis, at participant level (cluster‐RCTs) or study level (multiple study arms). GRADE assessments were provided for each outcome, but there was no evaluation presented of all included studies for risk of bias, and therefore it was not possible to report evidence appropriately	Systematic review and meta‐analysis drawing solely on quantitative evidence of effects without explicit model of behaviour, but collecting health outcomes data systematically along an implicit causal pathway (diarrhoea, helminth infection and nutrition)
WASH promotion/behaviour change outcomes	Lucas et al. (2011)	2010	The systematic review offered measured, reasonable conclusions based on the available evidence, but has *limitations*. Some aspects of the review were less clear, such as contacting expects in the search, or presenting findings using forest plots	Systematic review drawing on quantitative evidence presenting information on knowledge and behaviour change and discussing use of theory in the design of included intervention studies
	Null et al. (2012)	Unclear	The systematic review used clear inclusion criteria, and studies were analysed appropriately by randomised and nonrandomised designs. However, the review has *major limitations*. Methods to ensure internal study quality (searching and coding by independent team members) were not reported, and there was no critical appraisal of included studies	Traditional systematic review drawing solely on quantitative evidence of willingness‐to‐pay without explicit model of behaviour
	Fiebelkorn et al. (2012)*	July 2010	The systematic review of water treatment behaviour change covered a wide range of quantitative studies. However, the review has *major limitations*. The search was limited to studies published in peer‐reviewed English journals only, so the review was liable to publication and language biases. The review included behavioural outcomes but was restricted to studies reporting health outcomes, so was not comprehensive. Moreover, the review did not adopt a sufficiently comprehensive and transparent critical appraisal tool suitable to assess study bias. This was of concern because a very broad range of different study designs were included, but risk of bias was not considered when presenting findings. The review summarised use and follow‐up times across a broad range of studies, with no discussion of size and precision of effects, nor attempts to standardise outcome measures	Systematic review drawing on quantitative and qualitative evidence presenting information on behaviour change outcomes and discussing use of theory in the design of included intervention studies
	Evans et al. (2014)*	June 2013	The systematic review examined social marketing of water and sanitation products and used clear inclusion criteria. However, there are *major limitations*. There were restrictions on inclusion based on publication status, meaning that the review is liable to publication bias. It is unclear whether language bias was avoided. There was no risk of bias assessment for included studies, and the narrative synthesis used various methods of presentation ranging from effect sizes to vote counts. The review split the reporting of the study characteristics and results into numerous tables, but it is difficult to track which results corresponded to which study	Systematic review drawing exclusively on quantitative evidence along causal pathway (behaviour change and health outcomes)
	Morita et al. (2016)*	March 2016	The systematic review tackled an important topic for which there is limited evidence, but has *major limitations*. The search was not sufficiently comprehensive, excluding grey literature databases and contacting experts. The review was restricted to publications in English. The review is therefore at risk of publication and language bias. Additionally, the authors did not specify independent screening or data extraction by at least two reviewers. Risk of bias was assessed although not using a comprehensive tool, and risk of bias findings were not reported lines or make it clear which evidence was at a lower risk. Synthesis did not use meta‐analysis and forest plots, despite this appearing possible for several included outcomes (child faeces disposal and diarrhoea)	Systematic review and drawing exclusively on quantitative evidence along causal pathway (behavioural and health outcomes)
	Annamalai et al. (2016)*	December 2013	Inclusion criteria, screening and the synthesis processes for the quantitative and qualitative evidence included were clear. However, the systematic review has *limitations*. The authors utilised a vote‐counting approach for some quantitative data, and it is not clear that dependency in findings was adequately addressed	Theory‐based systematic review using explicit model and drawing systematically on quantitative and qualitative evidence along causal pathway
	De Buck et al. (2017)*	March 2016	The systematic review of sanitation and hygiene promotional interventions surveyed available quantitative and qualitative evidence and offered detailed analysis concerning handwashing and sanitation behaviour change. However, there are *limitations*. The search did not include checking of the reference lists of included studies to identify papers. The search was otherwise comprehensive, including contacting a larger number of stakeholders. Pooling of effect sizes was done using meta‐analysis, although opportunities were not taken to pool findings across outcomes, or to integrate findings from quantitative and qualitative synthesis components	Theory‐based systematic review and meta‐analysis using explicit model and drawing systematically on quantitative and qualitative evidence along causal pathway
	Garn et al. (2017)	December 2015	The systematic review of effectiveness of sanitation promotion on access and use of latrines has *limitations*. There was no table or summary of the assessment of bias assessment of each included study for each criterion, nor were findings analysed separately by risk of bias. The review did not provide sufficient detail concerning the participants in included studies. Finally, the review did not address unit of analysis errors, as it did not take clustering into account in the analysis (although clustered trials were included)	Systematic review and meta‐analysis drawing exclusively on quantitative evidence for behavioural outcomes
	Venkataramanan et al. (2018)*	March 2017	The systematic review of community‐led total sanitation used systematic methods to locate published and grey literature, summarising the findings on effectiveness separately from the findings on context and implementation. However, the review has *limitations*. The critical appraisal used a scoring scheme that is insufficiently transparent, and insufficient information was given in the synthesis, for example on confidence intervals. The authors noted some limitations of the review and did not draw strong conclusions for policy makers	Theory‐based systematic review using implicit behavioural model and drawing systematically on quantitative and qualitative evidence along causal pathway
	Marjorin et al. (2019)*	September 2018	The systematic review of promotional approaches comprehensively searched for literature in multiple languages, assessed risk of bias in included studies, and analysed studies by behavioural outcomes, diarrhoea, helminth infection and anthropometry). However, the systematic review has one *limitation*. Results are pooled separately by study design, but moderator analysis by risk of bias is not reported	Theory‐based systematic review using logic model and drawing systematically on quantitative evidence along causal pathway

* Review incorporates evidence on endpoint outcomes and measures of behaviour change.

The results showed that only five reviews were assessed as having “minor limitations”, thus meeting the criteria of “high confidence in the review findings” (Dangour et al., 2013; Ejemot‐Nwadiaro et al., 2015; Ejere et al., 2012; Rabiu et al., 2012). These reviews were restricted to RCTs and typically included few studies, hence were unable to support strong policy implications. Several reviews of sanitation omit a quasi‐experiment of sewage connections in Salvador da Bahia, Brazil (Barreto et al., 2007).^21^ Only two studies systematically incorporated quantitative and qualitative evidence using a mixed‐methods theory‐based approach (Annamalai et al., 2016; De Buck et al., 2017).

Overall, 13 reviews were assessed as being of “medium confidence”—but having limitations—and the remaining 22 reviews were of “low confidence”—that is, having major limitations. Reviews attaining “medium confidence” generally conducted systematic searches of published and unpublished literature but either used critical appraisal methods which were insufficiently transparent or did not account for some aspects of study design such as unit of analysis errors in cluster designs or lack of adjustment for study dependency (i.e., where evidence is double‐counted inappropriately). Studies appraised as being of “low confidence” were largely restricted to published literature, so they are liable to publication bias, inappropriate critical appraisal methods, risking confounding bias, and inappropriate methods of synthesis based on “vote‐counting” direction of effects (Hedges & Olkin, 1980).

Unfortunately, it is still fairly common for WASH systematic reviews to be restricted to studies published in peer review journals. These reviews therefore have inbuilt publication bias, defined as the greater likelihood for studies demonstrating positive and/or statistically significant findings to appear in peer review journals. A comprehensive search should cover both published and unpublished papers, and so avoid publication bias by which null, and possibly negative, findings are less likely to be published (e.g., Waddington et al., 2012).

It is also possible to test for, and correct, publication bias statistically, using clever methods that are based on hypothetical relationships between study size and probability of publication, called “small‐study effects” (Egger, 1997; Peters et al., 2008). Some meta‐analyses have done this (e.g., Clasen et al., 2007b, 2015; Curtis & Cairncross, 2003; Fewtrell & Colford, 2004; Waddington et al., 2009), although most reviews do not. Most recently, Wolf et al. (2018) stated “[t]here was no evidence of funnel‐plot asymmetry and small study effects in any of the WaSH meta‐analyses” included in that review (p. 519). This is a surprising finding, as publication bias is usually found in all disciplines (e.g., Rothstein et al., 2005). However, examination of their funnel graphs indicates they did not use methods of small‐study analysis which take into account other sources of funnel graph asymmetry, such as risk of bias in effect estimation (Peters et al., 2008).

As noted above, the quality of self‐reported diarrhoea morbidity has been questioned, particularly when based on repeated measurement (Peterson Zwane et al., 2011; Schmidt & Cairncross, 2009). It appears to be increasingly common for systematic reviews to adjust for lack of blinding using Bayesian methods. Hunter (2009) was the first to propose a bias correction procedure to water treatment studies using bias coefficients from meta‐epidemiology findings, presented in Wood et al. (2008). In the updated Cochrane water treatment review by Clasen et al. (2015), similar bias correction factors were also applied, although the authors note that “we urge caution in relying on these adjusted estimates since the basis for the adjustment is from clinical (mainly drug) studies that may not be transferable to field studies of environmental interventions” (p. 9). Freeman et al. (2014) and Wolf et al. (2018) also adjusted for bias due to lack of blinding, including hand hygiene interventions, but not water supply and sanitation, arguing that water supply and sanitation have recognised benefits over and above health impacts, whereas water treatment and hygiene “usually aim exclusively to improve health which is apparent to the recipient” (p. 512). However, the hygiene adjustments lead to the estimated ineffectiveness of hygiene in combating diarrhoea illness, which is not supported by high confidence systematic evidence (Ejemot‐Nwadiaro et al., 2015).

There were no overall trends in confidence in the findings over time. As would be expected of standards setting agencies, reviews conducted under the auspices of systematic review standards‐setting organisations such as Cochrane and the Campbell Collaboration were nearly always done to higher standards of confidence. These include, for example, avoiding language bias by searching for and incorporating studies in languages other than English, of which relevant studies of WASH interventions are known to exist in Chinese, French, Portuguese and Spanish. However, although stating they are open to incorporating studies in other languages, most systematic reviews, including this evidence map, do not actually search non‐English language databases such as Latin American and Caribbean Health Sciences (LILACS) or the China National Knowledge Infrastructure (Fung, 2008; cited in Clasen et al., 2010), or search general resources using non‐English language keywords. Clasen et al. (2010) is therefore particularly noteworthy as they searched these databases, identifying seven additional studies not included in any other systematic reviews of sanitation, more than doubling the number included in the review to 12 studies.

Finally, a large proportion of reviews have been conducted without protocols, risking publication bias in the findings of the review or analyses conducted, although there is a trend recently for some new reviews to indicate “protocols are available on request”. Protocols are important, particularly where they undergo peer review, to provide the scope of research, including to avoid duplication of effort, and prevent results‐based decision making in analysis. Deviations from protocol are possible, and should be indicated clearly the published review, as required of all studies for Cochrane and the Campbell Collaboration.

On quantitative evidence appraisal and synthesis, reviews tended to score highest on effect size calculation and reporting of heterogeneity—that is, they calculated effect sizes using comparable metrics and statistically analysed between‐study differences. Reviews did worse on critical appraisal (using appropriate risk of bias assessment), synthesis methods (including reporting findings by bias categories), and worst on reporting characteristics of included studies (usually due to lack of independent coding by two reviewers).

On the use of mixed methods approaches, including formative evidence from qualitative studies, there seems to have been an evolution in approaches in WASH sector systematic reviewing. Some authors incorporated qualitative evidence contained in the studies eligible for the quantitative review of effects (e.g., Waddington et al., 2009). This approach could be a model for evidence synthesis if the impact evaluations on which reviews of effects are based typically use theory‐based approaches, where they report comprehensive information on the intervention (what was provided, to whom, by whom, at what time), and also present outcomes along the causal pathway, including intermediate and endpoint outcomes. However, as this is often not the case (e.g., White, 2009) mixed‐methods systematic reviews need to be increasingly inclusive, usually by undertaking additional searches for qualitative studies linked to the included quantitative studies or by conducting full searches for qualitative studies to answer specific review questions, as done by De Buck et al. (2017) and Venkataramanan et al. (2018).

It is at the initial stages of the review process that formal guidance is most lacking on effective mixed methods approaches, especially convening the study team and constructing the conceptual framework to support the integration of qualitative and quantitative evidence. Establishing teams with appropriate qualitative and quantitative skills, preferably drawing on broad academic disciplines, is usually needed for high quality mixed methods reviews to be done efficiently (White, 2018).

### 5.5 Areas of evidence from systematic reviews

This section summarises policy‐relevant findings from systematic reviews with high or medium confidence (limitations or minor limitations, respectively).

#### 5.5.1 Water improvements

The first systematic review of water improvements concluded that “safe excreta disposal and proper use of water for personal and domestic hygiene appear to be more important than drinking water quality in achieving broad health impacts” (Esrey et al., 1991, p. 31). A subsequent meta‐analysis by Clasen et al. (2007b, updated in 2015) found water treatment at point‐of‐use (POU), particularly filtration, was more effective in reducing diarrhoea risk than other types of water improvements, on average by about 40% in LMICs. The findings suggested that other methods of household water disinfection (e.g., chlorination) only reduced diarrhoea infections by one‐fifth, if at all, due to biases. Water treatment was found to be more effective when a safe water storage container was also provided (Clasen et al., 2015), as they are for water filters from which drinking water is accessed from a tap. However, reviews have also raised concerns about bias in self‐reporting diarrhoea outcomes (Clasen et al., 2015; Waddington et al., 2009), adherence to water treatment technology (Arnold & Colford, 2007; Clasen et al., 2015; Waddington et al., 2009), and consequently lack of sustained health impacts (Waddington et al., 2009). Higher quality studies also found piped water to households significantly reduced diarrhoea (Waddington et al., 2009).

#### 5.5.2 Sanitation improvements

Until the last decade there were few impact evaluations of improving access to sanitation facilities, such as a latrine or sewer connenction, covering a small number of clusters. Replacing on‐site sanitation with water‐based sewerage was estimated to reduce the incidence of diarrhoea by 30% (Waddington et al., 2009), though it may not always be a suitable solution given the maintenance costs. However, early reviews had not taken clustering into account. The Cochrane review by Clasen et al. (2010) did not conduct meta‐analysis because none of the studies at that point had taken clustering of observations into account in calculating standard errors.

The evidence suggests that community‐based approaches to participatory psychosocial triggering, and social marketing are more effective than other approaches in promoting sanitation behaviour change (De Buck et al., 2017). CLTS may be effective at reducing open defecation in trials, at least in the short term, but this evidence does not corroborate the wide‐spread claims of ending open defecation found in village case studies using qualitative evaluation designs without control groups (Venkataramanan et al., 2018). De Buck et al. (2017) also synthesised evidence from qualitative studies on the implementation of hygiene and sanitation promotion. They concluded that factors such as community involvement in design and implementation, as well as interpersonal communication in communication strategies, were particularly effective when incorporated into programmes.

#### 5.5.3 Hygiene improvements

A review of the effects of handwashing on respiratory illness did not find any studies in LMICs (Rabie & Curtis, 2006). Meta‐analyses suggested hand‐hygiene interventions reduce reported gastrointestinal illness by between 30% and 50% (Cairncross et al., 2010; Curtis & Cairncross, 2003; Ejemot‐Nwadiaro et al., 2015; Waddington et al., 2009). Hygiene education with soap provision appears to be the most effective technology, as soap is more effective in the mechanical removal of pathogens than water alone. Issues affecting the quality of self‐reported diarrhoea morbidity also affect hygiene interventions (Ejemot‐Nwadiaro et al., 2015). Provision of soap and other water quality interventions may also lead to a small increase in height for children under the age of five (Dangour et al., 2013). There is also evidence that handwashing interventions are protective against diarrhoea for people living with HIV. However, there are no studies of the effects of water supply or sanitation interventions for immunocompromised populations (Peletz et al., 2013).

The evidence suggests that community‐based approaches to participatory psychosocial triggering, where a two‐way dialogue is established, are particularly effective in promoting handwashing (De Buck et al., 2017).

### 5.6 Major gaps in the evidence

Given the large body of available evidence, researchers and funders need to consider carefully where there is a need for new primary evidence, whether from trials or natural experiments exploiting existing data sets, and new evidence syntheses, such as systematic reviews. The EGM suggests these priority areas for future primary research:

- Impact evaluations of the effectiveness of interventions delivered, and for use, in health facilities in LMICs.
- Impact evaluations collecting data on understudied quality of life outcomes, notably ARIs, time use, musculoskeletal disorders, psychosocial health, empowerment, safety, household income, and long‐term wage earnings resulting from WASH improvements experienced during childhood. Measures such as changes in time use (productive, reproductive, leisure and sleep) can be collected through household surveys or observation, and are sufficiently important for measuring the totality of effects of WASH interventions that they should be included in trials using standard measurement protocols.
- Longer‐term measurement of adherence (sustained behaviour change) and quality of life improvements, at least one year after implementation and preferably at longer follow‐up periods. Most large‐scale trials already tend to be funded by multiple donors, and there may be greater opportunities than presently taken for funders to dovetail on one‐another's efforts by providing resources for additional follow‐ups.
- In addition, more comparative studies of “add‐ons” to standard interventions or alternative delivery mechanisms to promote WASH technologies in the same contexts are needed. These include, for example, approaches to combat problems of adherence, in particular reducing slippage back to open defecation in CLTS, or the extent to systems‐based approaches (e.g., subsidies or microfinance to consumers or latrine producers) need to be provided alongside CLTS in certain contexts. Rigorous impact studies testing different methods of implementation in particular countries could also help funders of global WASH programmes implement more effective programmes. These include impact evaluations with multiple trial arms (factorial design) and active controls, measuring successes in achieving and sustaining outcomes in particular contexts.
- More evaluations collecting data on objective measures of gendered behaviour change, health and socioeconomic outcomes whenever possible, or using methods to reduce known biases in reporting by study participants and researchers. For example, there are greater opportunities than presently taken to (at least partially) blind participants, observers and data analysts to intervention. These include evaluations that employ gender analysis to better understand not only differential outcomes but different norms and barriers that need to be addressed during intervention design.
- Impact evaluations of interventions targeting, or presenting disaggregated data for, vulnerable populations, particularly over the life‐course and for people living with a disability. Evaluations are urgently needed of interventions targeting vulnerable populations, particularly nonbinary or transgender individuals and people living with disabilities, or at least measuring outcomes among these groups. If the sample sizes typical of impact evaluations are not sufficient to detect changes among these groups with sufficient statistical power, estimates can be pooled using meta‐analysis of multiple studies collecting consistent outcomes data among these groups.
- Evaluations using natural experimental approaches, which, like RCTs, are able to control for unobservable confounding, but, unlike RCTs, do not distort the natural process of intervention roll‐out or use outcome data collection methods that participants may link to intervention receipt or otherwise distort due to Hawthorne effects or reporting biases. Rigorous study designs that are underexplored in WASH sector impact evaluation include regression discontinuity and ITS.
- Finally, there are important gaps in study reporting and design. Prospective impact evaluations need to comply with basic reporting standards according to CONSORT, in particular the reporting of participant flow from identification, to allocation, intervention roll‐out and follow‐up surveys (together with reasons for losses to follow‐up by intervention group). Retrospective impact evaluations should publish preanalysis plans or study protocols, where deviations from protocol can be simply reported transparently (as they are done as standard in systematic reviews). All prospective studies should undergo ethical review, including in the country where the data are being collected.

The map also suggests the following priority areas for evidence synthesis:

- Inadequate WASH is thought to kill 1.6 million people per year globally, half of which are due to diarrhoea (Prüss‐Ustün et al., 2019). A major omission from the current systematic review evidence base is the lack of a review focusing on the impacts of WASH interventions on mortality, whether all‐cause or due to specific factors like diarrhoea and ARIs. A review is currently underway, drawing on the evidence collected in this map (Waddington & Cairncross, 2021).
- A systematic review update is urgently needed of the effects of water supply and hygiene on respiratory infections. The most useful systematic reviews collect and synthesise evidence on outcomes along the causal pathway including immediate (service access), intermediate (service use) and endpoint (health, social and economic) outcomes, as well as cost‐effectiveness or cost‐benefit evidence. Policy relevant evidence synthesis may also need, at the very least, to engage with available programme documents to articulate correctly intervention design and implementation for promotional approaches, and contacts with implementing bodies are very likely to be required for accurate estimation of costs. Engagement with programme documentation and qualitative literature may also help in integrating gender analysis into the framework of the reviews.
- High quality synthesis of studies from existing impact evaluations, such as community‐driven approaches, microfinance and WASH in schools, as well as time savings associated with water and sanitation improvements and water use and water behaviours and use associated with water supply and water quality improvements.
- Syntheses looking at the differential impacts of using different mechanisms for providing a WASH technology, provided alone or in combination, including syntheses of complex interventions including non‐WASH sector cointerventions (e.g., deworming or nutrition supplements).
- Syntheses of evaluations of alternative intervention mechanisms, using network meta‐analysis, to achieve and sustain outcomes in particular contexts, such as CLTS versus subsidies.
- Incorporation of WASH cointerventions into existing WASH sector evidence mapping efforts, and undertaking evidence mapping of WASH interventions in places of work, commerce, recreation, streets, fields and transit hubs.

## 6 DISCUSSION

This section contextualises the results by examining the patterns and observed changes in the literature base, as well as taking a holistic look at the evidence base overall.

### 6.1 Summary of main results

#### 6.1.1 Characteristics and trends of the evidence base from impact evaluations

WASH impact evaluations have been conducted in 83 LMICs. The distribution of studies is uneven with high concentrations of studies in Bangladesh, India, and Kenya, which have 50 study arms each.

Over the last decade the focus of practitioners, policymakers, and researchers alike has shifted from “what” to provide to “how” to do so, and “where”. This change is reflected in the research covering a broader set of intervention mechanisms. In particular, there has been an increased focus on BCC that uses psychosocial “triggering” and a shift away from direct provision, particularly of latrines. In sanitation, this is most commonly CLTS, which aims to reduce open defecation and increase the use of latrines by leveraging social cohesion to make collective behavioural changes, but can also include information campaigns focused on disgust or being a good parent. Having said this, the traditional approaches of directly providing hardware (e.g., handing out water filters) or health messages (factual information about the consequences for health of not washing ones hands) remain the most common interventions mechanisms even among new studies.

The International Year of Sanitation, in 2008, brought attention to the importance of sanitation technologies, and major donors like Gates Foundation brought resources for doing studies, especially RCTs, in LMICs. Before this, interventions to provide clean drinking water and hand hygiene had been the priority for intervention research and many studies had looked at how to improve water quality. Water treatment is still the most studied WASH technology (over one‐quarter of the total), but the percentage of studies looking at sanitation has nearly doubled and there has also been an increase in studies examining hygiene promotion. Up until 2008, only six studies had been conducted on promoting, or providing, latrines; there are now over 50.

“Where”—the place of use dimension—further distinguishes technologies that are physically tied to a geographic location. Traditionally hardware was either provided directly to a household or for communal use by a village, informal community, or neighbourhood. There has, however, been increasing interest in the effect of providing WASH facilities at schools over the last 20 years and the SDGs have highlighted the need to incorporate both schools and health facilities. The vast majority of impact evaluations provide hardware directly to households. Research has also moved with shifting interests and while there were only two rigorous studies specifically examining WASH infrastructure in schools before 2008, there are now 39. The majority of these combine water, sanitation, and hygiene technologies as a comprehensive overhaul for the school. Only one controlled study was found on handwashing supplies in healthcare facilities, and none were found in healthcare facilities for any other kind of technology.

In line with the other changes, there has been a shift in the commonly reported outcomes. While diarrhoeal disease, particularly carer‐reported diarrhoeal morbidity among children, remains by far the most commonly reported outcome, there has been a big increase in the number of studies reporting on behavioural outcomes (see Figure 9). This is an important change as the principal argument used by proponents of alternative delivery mechanisms is that they are more effective at changing these behaviours and therefore improving lives. However, interventions which foster marginal improvements in personal WASH behaviour may not cause sufficient changes at community levels to improve quality of life outcomes like child nutrition or diarrhoea mortality.

The most commonly reported on behaviours are handwashing, water treatment and handling, and latrine use. Many of the studies reporting on hygiene behaviour, include measures of personal food hygiene; nearly 50 studies specifically look at handwashing before food preparation, five report on the microbial contamination of food or eating utensils, and 17 report on other food hygiene outcomes, such as whether food is stored properly and dishes washed appropriately. It is important that hygiene studies examine food hygiene outcomes, given the importance of food in faecal‐oral disease transmission (Wagner & Lanoix, 1957). There has also been an increase in the reporting of social and economic impacts. This is principally driven by a large increase in the number of studies reporting measures of education and cognitive development, and mainly reflects the increase of studies being conducted in schools.

However, outcomes data collection, whether on behaviour or quality of life, remains heavily reliant on self‐reporting, which is highly susceptible to biases, including social desirability (courtesy) bias.^22^ Some studies used observation, hospital records, and medical samples to improve the objectivity of the measures (e.g., Moraes et al., 2003) but this remains rare. Research in the sector may also underutilise recent advances in measurement, such as list experiments (Karlan & Zinman, 2012) which aim to elicit revealed preferences from survey participants about undesirable behaviours, and anchoring vignettes, which can efficiently generate comparable measures of outcomes like empowerment (King et al., 2004).

Despite the importance of sustaining the use of hardware and preventing slippage back to open defecation, and other poor practices, only 18 studies measure sustainability of behaviours, defined here as being measured 12 or more months after implementation. Even this is most commonly related to sustained handwashing practices rather than, for example, sustainability of latrine use or community ODF status, which is only measured in three completed latrines studies (Alzua et al., 2015; Crocker et al., 2017 in Ethiopia and Ghana; Orgill, 2017 in India) and in one study of CLTSH in Ethiopia (Delea et al., 2020).

Existing impact evaluations cover rural, urban, and slum populations with the vast majority either targeting or including rural communities (295 studies), which are usually the most disadvantaged areas in terms of WASH service provision. As noted below, however, more studies are needed to address the “for whom” technology aspects among vulnerable groups.

Finally, over half of controlled study designs now use randomised assignment, indicating the extent of support in academic and research funding communities for the approach. Some RCTs are taking full advantage of the power of the methodology by conducting comparative designs with prospective randomised assignment at baseline to alternate intervention delivery mechanisms. Guiteras, Levinsohn, et al. (2015) provide an example in Bangladesh comparing community promotion (CLTS) with subsidies. Other recent examples of comparative designs include Luby et al. (2018) in Bangladesh and Null et al. (2018) in Kenya which compare water, sanitation, hygiene and nutrition support interventions individually and in combination.

A small number of studies are taking advantage of natural experiments using existing data to conduct rigorous, and potentially highly cost‐effective, evaluations, such as RDDs which estimate the effects of interventions which are assigned to individuals or communities based on a threshold on a continuous variable, for example a poverty index or an administrative boundary (Spears, 2013; Ziegelhofer, 2012). Methods used to analyse data in natural experimental frameworks include IV estimation (Ziegelhofer, 2012) and difference in differences (Galiani et al., 2005). However, natural experiments applying rigorous methods to existing surveys remain an under‐utilised methodological approach in the WASH sector, given the large numbers of existing household survey datasets available that contain questions on access to WASH services which, for example, could be analysed as pseudo‐panels using double‐difference methods. It is also important to note that there continues to be a great number of uncontrolled studies that simply measure outcomes before and after the intervention. Most of these studies have been excluded from the map as they are not usually able to attribute changes to the intervention. The exception is for some immediate outcomes such as time spent collecting water immediately before and after a new water supply is provided, as noted above.

#### 6.1.2 Characteristics and trends of the evidence base from systematic reviews

A systematic review will be most relevant when the methodology is applied to a clearly defined research question, and preferably where eligible evidence is known about a priori. A common approach used in WASH systematic review and meta‐analysis is to ask a question answerable using health impact evaluations; for example, “interventions to improve water quality for preventing diarrhoea” (Clasen et al., 2015). In recent years, there has also been a movement towards reviews covering multiple research questions answerable using different types of evidence, such as “effectiveness and factors influencing implementation of handwashing and sanitation promotion” (De Buck et al., 2017). Broader reviews enable greater statistical precision and systematic analysis of bias, as noted by Gøtzsche (2000): “[a] broad meta‐analysis increases power, reduces the risk of erroneous conclusions, and facilitates exploratory analyses which can generate hypotheses for future research” (p. 586).

A related issue is whether to set the question around an outcome—for example, “water, sanitation and hygiene to tackle childhood diarrhoea morbidity in low‐ and middle‐income countries” (Cairncross et al., 2010; Fewtrell & Colford, 2004; Waddington et al., 2009; Wolf et al., 2014, 2018)—or an intervention—“effect of hand‐washing on infectious diseases” (Aiello et al., 2008). Some would further delimit by combining the two; for example, “effect of hand‐washing on diarrhoea” (Ejemot‐Nwadiaro et al., 2015), or perhaps “the effect of improved water supply on women's time use” (a review which remains to be undertaken). But others might argue that hygiene can have a broader range of benefits in fighting respiratory infections (Rabie & Curtis, 2006), and so should not be assessed on its impact on diarrhoea alone.

Another example is the effectiveness of WASH improvements on diarrhoea, which are expected to be bigger when access to water and sanitation facilities in the comparison condition is worse (Fewtrell & Colford, 2004). One area where there does appear agreement is on the splitting of evidence collected under endemic versus epidemic conditions, since the effects of WASH in outbreaks are known to be much larger (e.g., Curtis & Cairncross, 2003; Gundry et al., 2004). This also includes emergency situations, where separate reviews of WASH have been completed (Brown et al., 2012; Yates et al., 2017).^23^

Early reviews of diarrhoea did examine behaviour change—household water treatment in the case of Gundry et al. (2004) and Arnold and Colford (2007), and water treatment, hand hygiene and sanitation use in the case of Waddington et al. (2009). But it is also becoming more common for reviews to focus solely on behaviour change (e.g., Garn et al., 2017; Lucas et al., 2011). What is innovative is, firstly, that reviews are collecting and meta‐analysing effect sizes relating to behavioural outcomes along the causal pathway (e.g., Ejemot‐Nwadiaro et al., 2015). It seems to be increasingly recognised that causal pathway analysis is highly policy relevant as it enables an understanding of heterogeneneity and therefore the circumstances in which review findings are applicable (e.g., Waddington et al., 2012; White, 2018). Secondly, some reviews are starting to change the focus from WASH technologies to specific intervention mechanisms like social marketing (Evans et al., 2014), sanitation and hygiene behaviour change promotion (De Buck et al., 2017), and CLTS (Venkataramanan et al., 2018). These two latter reviews also used mixed methods to synthesise impact evaluations and qualitative studies.

It is debatable whether analysis of behaviour requires incorporation of qualitative evidence systematically. Impact evaluations and systematic reviews drawing solely on quantitative evidence from impact evaluations are commonly thought to be unable to answer questions about why interventions are successful or not. However, theory‐based systematic reviews, which draw on an explicit theory of change (or logic model) and collect evidence on outcomes along the causal pathway, can explain heterogeneity in findings. For example, studies that have explained variation in quality of life outcomes have done so with reference to correlations (or lack therefor) with programme adherence (Waddington et al., 2009; Welch et al., 2017). However, analysis of lower reaches of the causal chain, in particular the “intervention black box” usually requires programme literature and qualitative evidence (White, 2018). Mixed‐methods reviews can answer the most pressing questions for policy and practice—design, implementation, participation, targeting, adverse or unintended effects, cost‐effectiveness (Waddington et al., 2018). For example, the systematic review by De Buck et al. (2017) covered the full range of relevant behavioural and health outcomes associated with hygiene and sanitation provision, incorporating systematic qualitative evidence on “barriers and enablers” of implementation and adherence.^24^

#### 6.1.3 Equity and vulnerable populations

Impact evaluation research in WASH does pay attention to the specific needs of vulnerable groups, but there is still some way to go before most studies achieve the full integration of gender and other equity concerns, to become a consistently transformative sector (IGWG, 2018).

Globally women and girls carry most of the burden of water collection (including time, calories spent, musculoskeletal injuries, and risk of assault by people or attack by wild animals) and having to use unsafe places to defecate, where water and sanitation services are not accessible. They also experience particular hardships where inadequate services constrain menstrual hygiene management. In the last ten years, new studies have been conducted that evaluate interventions and outcomes that disproportionately affect women and girls; this includes measuring time use (22 studies), psychosocial health outcomes (7 studies), and safety and vulnerability (4 studies), as well as MHM. However, gender analysis is rarely used as part of the framework for understanding programme effects in impact evaluations and systematic reviews. In fact, only a minority of studies included in the map (19% of impact evaluations and 20% of systematic reviews) reported sex disaggregated outcomes. In impact evaluations, psychosocial health (43%), education and cognitive development (40%), open defecation (33%), and time use (26%) were some of the most commonly sex disaggregated outcomes. It is also worth noting that there were no rigorous impact evaluations of sanitation for nonbinary or transgender individuals.

The findings are even more sparse when looking at other categories of vulnerability. One impact evaluation (Abebe et al., 2014) and two systematic reviews (Peletz et al., 2013; Yates et al., 2015) looked at people living with HIV, who often have different social constraints and medical needs. Two impact evaluations (Doocy & Burnham, 2006; Roberts et al., 2001) specifically looked at the needs of those in refugee camps. One impact evaluation (Chase et al., 2002) and two systematic reviews (Brown et al., 2012; Ramesh et al., 2015) examined those living through, or in the aftermath of, a humanitarian crisis. Finally, and most strikingly, no controlled evaluations examined WASH interventions that either targeted people living with a disability or the success of standard WASH interventions in improving outcomes for them.

### 6.2 Overall completeness and applicability of evidence

The map provides a comprehensive look at WASH interventions for promoting the uptake of safe water, sanitation, and hygiene in LMICs. It includes interventions to improve WASH in the private and public domains, including households, communities, schools, and health facilities. It excludes WASH interventions in the places of commerce, recreation, streets, fields, transit hubs and the workplace.

The overall objective was to map the existing, and ongoing, published and unpublished research in the WASH sector. With over 350 completed impact evaluations, and over 40 systematic reviews, this is a large and growing body of evidence. However, there are concentrations of research on diarrhoeal illness, water treatment technologies, and direct WASH provision, hence other mechanisms, technologies, and outcomes remain understudied. Research has also concentrated in Bangladesh, India, and Kenya, with around a third of studies being conducted in these three countries alone.

### 6.3 Quality of the evidence

The review of quality of impact evaluations suggested methods for causal identification have generally improved, with the availability of more rigorous methods and, importantly, research resources from large organisations including new philanthropic bodies. However, there are important areas for improvement around reporting of studies, in particular standards for participant flow through prospective trials of WASH interventions remain very low. More retrospective studies could also use pre‐analysis plans, which are now commonly done in trial research.

The review of systematic reviews suggests methods of synthesis remain strong, but some recent reviews are limited to published studies only. This is known to cause inbuilt publication bias, undermining the credibility of systematic evidence and possibly also policy uptake.

### 6.4 Potential biases in the mapping process

The map was constructed using rigorous search, screening, and data extraction processes based on best practices for a systematic review. More than 20 sources, including databases of both published and grey literature, were searched and each study was screened independently by at least two‐authors at both the title and abstract and full‐text stages. The references of included systematic reviews were also searched for eligible studies not captured in other searches. The data extraction conducted in duplicate, and significant training was given to coders to check for any discrepancies in the coding process.

The main area of limitation in the review is that most of the searches were completed in 2018. It seems unlikely that many prospective impact evaluations or systematic reviews have been omitted, because the searches were done for completed and ongoing studies, and citation tracing was done for ongoing studies in 2020 to capture any studies completed thereafter. It is very likely, however, that retrospectively designed impact evaluations and systematic reviews conducted without protocols published since 2018 have been omitted. Although there were studies in French, Spanish, Portuguese and Chinese included in the map, the searches were conducted in English so publications in other languages may have been missed.

### 6.5 Limitations of the evidence map

The first important limitation to understand is that this map is not a systematic review. The findings of the impact evaluations are therefore not synthesised. The objective of this map is to assist in in policy recommendations, and guide what future primary research and systematic reviews are conducted to maximise efficiency. While some attempts to assess the robustness in design, conduct and reporting of included impact evaluations was done, full critical appraisal of that evidence—done to standards in high quality systematic reviews—was not undertaken.

Additionally, limits were placed on scope as the WASH sector is very broad and overlaps with many other disciplines. For example, interventions in food hygiene in the workplace (e.g., a market), vector control, agriculture, and environmental protection were excluded. Finally, as a deviation from protocol, non‐WASH cointerventions were excluded. The most common cointerventions were deworming programmes and nutritional education or supplements.

The final limitation is that the map does not include any qualitative evidence that could help in understanding the contextual and implementation factors that may affect the success of the projects. Importantly, some of the systematic reviews do conduct this type of analysis, although not all were rigorous and relevant reviews were eligible for inclusion in the map, for example a review on incorporating the life‐cycle approach into WASH policies and programmes (Annamalai et al., 2016).

## 7 AUTHORS' CONCLUSIONS

Ensuring that everyone has access to appropriate water, sanitation, and hygiene facilities is recognised as one of the most fundamental challenges in international development. Rigorous evidence about what approaches work in improving equitable access to water and sanitation is important as governments and NGOs around the world work towards the SDGs. This EGM was produced to help decision makers access systematic evidence about different options for promoting access to WASH in households, communities, schools and health facilities.

Rigorous evaluations of the effects of WASH sector interventions have been conducted in LMICs since the 1970s. “First generation” WASH sector impact evaluations focused on the effects of WASH services provision, often under ideal conditions, on diarrhoeal disease. Since the International Year of Sanitation, 2008, and the influx of resources for WASH research from major funders like Gates Foundation, there has been a behavioural revolution corresponding to the emergence of a “second generation” of WASH impact evaluation research. Similarly, systematic reviews of WASH provision have been produced since the 1980s before “systematic review” became a common term. There is a similar shift towards reviews examining behavioural interventions and outcomes.

While access to clean water is still an important issue, there has been increasing research on the impact of sanitation and hygiene promotion since 2008. Of particular importance is the increasingly deep body of evidence on approaches that use psychological and social pressures, as well as those that elicit behavioural change through market‐oriented approaches.

Overall, the map suggests the community of WASH impact research is a leading sector in the production of rigorous evaluations on an increasingly broad set of intervention mechanisms, WASH technologies, outcomes, and places of use.

### 7.1 Implications for practice and policy

Existing systematic reviews with medium or high confidence in methods and reporting can support policy and programme decision‐making around the benefits of WASH for reducing enteric infections like diarrhoeal disease and helminth infections. There is evidence that integrating BCC reliant on psychosocial “triggering” can improve the effective uptake of sanitation and hygiene technologies. However, the evidence base probably remains too small to draw conclusions about particular promotional strategies (e.g., CLTS), technologies (e.g., MHM), and, especially, outcomes like psycho‐social heath and musculoskeletal disorders.

### 7.2 Implications for future research

There is a need for more rigorous primary research on:

- impact evaluations of WASH in health facilities, particularly those in, and used by, residents of low‐income communities;
- understudied outcomes, such as respiratory infections, time use and psychosocial health;
- intervention mechanisms, such as “add‐ons” to CLTS and social marketing to promote sustainability and reduce slippage rates;
- vulnerable populations, particularly people living with a disability.

The reliability, and applicability, of impact evaluations could be improved by using more objective measures of outcomes and integrating gender analysis more often into programme frameworks. There may also be more opportunities than are currently being taken to conduct rigorous, cost‐effective, evaluations using existing data and natural experiments.

As a minimum it should be standard practice for authors of prospective studies to report—and funders and journals to require—participant flow diagrams. This lack of transparency makes it difficult to appraise validity in prospective studies. It is clear that these failures stifle scientific progress, and WASH triallists should accept as good practice standards adopted in clinical epidemiology more than two decades ago (Moher et al., 1998).

More systematic reviews are needed where there are sufficient existing or planned impact evaluations, such as for diarrhoea mortality, for which one review is ongoing (Sharma Waddington & Cairncross, 2020), WASH in schools, and community‐based approaches. The usefulness of reviews can be improved across the board by drawing clearer distinctions between different mechanisms for providing technologies and synthesising the evidence, where available, on outcomes along the causal chain including immediate outcomes (service access), intermediate outcomes (WASH behaviours) and health and socioeconomic impacts. Adopting mixed methods approaches may also increase the usefulness of reviews of behavioural approaches since they can provide information on barriers and enablers affecting implementation. More systematic incorporation of cost‐effectiveness analysis into reviews would be an advantage, probably requiring partnerships with implementing bodies to obtain data.

## CONTRIBUTIONS OF AUTHORS

The report was written by Hugh Sharma Waddington and Hannah Chirgwin. Hugh Sharma Waddington, Hannah Chirgwin, and Sandy Cairncross designed the protocol, including conceptual framework, study inclusion and search strategy, which was also developed with the help of John Eyers, information retrieval expert with Campbell International Development Coordinating Group. Searches were undertaken by Hannah Chirgwin, Hugh Sharma Waddington, and Dua Zehra. Data were collected from studies by Hannah Chirgwin, Hugh Sharma Waddington and Dua Zehra, supported by Miriam Berretta, Hastings Chipungu, Raj Popat, Yashaswini PrasannaKumar and Abubeker Tadesse. Hugh Sharma Waddington will be responsible for updating this EGM.

## DECLARATIONS OF INTEREST

The authors have no vested interest in the outcomes of this evidence map, nor any incentive to represent findings in a biased manner. Sandy Cairncross has been involved in the development of sanitation and hygiene interventions. Sandy Cairncross and Hugh Sharma Waddington have led systematic reviews included in the map, and Sandy Cairncross has also led and contributed to included impact evaluations. Inclusion decisions and critical appraisal of work by these two authors were done by other members of the team. Hannah Chirgwin now works for the FCDO, including on WASH programming, but was not affiliated with any of the included projects at time of conducting this study. We are not aware of any other conflicts that might affect decisions taken in the review and results presented.

## PLANS FOR UPDATING THE EVIDENCE MAP

The authors plan to update the map (or support others in doing so) when sufficient further studies and resources become available.

## DIFFERENCES BETWEEN PROTOCOL AND MAP

Studies with a non‐WASH cointervention were excluded if the effect of the WASH intervention could not be isolated. The intervention categories were reorganised into supply, demand and mixed approaches, based on the helpful comment from a peer reviewer.

## SOURCES OF SUPPORT

We thank WSSCC, now the Sanitation and Hygiene Fund, for funding this update and JICA for funding the initial WASH evidence and gap map in 2014 (Waddington, PrasannaKumar, & Caincross, 2014), with technical support by the World Bank Independent Evaluation Group.

## ROLES AND RESPONSIBILITIES

The report was written by Hannah Chirgwin and Hugh Sharma Waddington. Hannah Chirgwin, Hugh Sharma Waddington and Sandy Cairncross designed the protocol, including study inclusion and search strategy, which was developed with the help of John Eyers, information retrieval expert with Campbell International Development Coordinating Group. Searches and data collection were done by Hannah Chirgwin, Hugh Sharma Waddington and Dua Zehra. Hugh Sharma Waddington will be responsible for updating this evidence and gap map.

## Supporting information

Supporting information.

Supporting information.

Supporting information.

## Footnotes

1 See: https://www.who.int/publications/i/item/WHO-2019-nCoV-IPC-WASH-2020.4, accessed November 30, 2020.

2 UN Water also produces an annual synthesis report on progress against SDG6 targets (UN Water, 2018).

3 Other public places are not included in this map, such as workplaces, markets and transport hubs. Due to the global pandemic, the policy conversation has broadened to include these high transit areas. These would be suitable topics for future WASH evidence maps and updates.

4 The caveat is that the standards of searching undertaken in evidence mapping are usually not as exhaustive as those for systematic reviews. For example, sources may be limited to English language or by date; reference snowballing (citation tracing and bibliographic back‐referencing) may not be undertaken. However, to produce this WASH evidence map, searches were done to the standards that would be taken in a “high confidence” systematic review (Lewin et al., 2009), including searches for ongoing studies.

5 The initial WASH evidence map (Waddington et al., 2014) included behaviours as a secondary outcome only—that is, it coded information about behaviour from studies that were eligible for inclusion on primary outcomes (ill‐health, nutrition, mortality and socioeconomic impacts).

6 We are grateful to the peer reviewer who suggested differentiating supply‐ and demand‐side interventions.

7 Regulations targeting providers are included in supply‐side interventions under “improving operator performance”.

8 This criterion is the same as that used by Clasen et al. (2007b, 2010, 2015) and Wolf et al. (2014, 2018).

9 This designation also applies to RCTs with noncompliance that are analysed using ATET.

10 IRC was originally known as the International Reference Centre for Community Water Supply until the mid‐1980s when it changed its name to IRC International Water and Sanitation Centre, but as of 2014 simply goes by IRC. We use IRC (WASH) to distinguish it from the International Rescue Committee.

11 Reference snowballing, as used in the searches for this evidence map (see below), may enable the studies missed by electronic searching to be identified.

12 Neither final report nor protocol (if available) was cited by either study team. A systematic review of child faeces disposal interventions, covering some of the same included studies as de Buck et al. (2017), was completed recently (Majorin et al., 2019). These reviews also appear to have been done independently, as neither study cites the other.

13 Due to restrictions on study design, only 34 studies were eventually included. Sixteen studies featured in neither de Buck et al. (2017) nor Garn et al. (2017), although five of these were published in 2017, presumably after the searches in those reviews had been completed.

14 Study reports were assessed as to whether they included all outcomes stated in trial records. For example, respiratory and helminth infection data indicated in the Colford (2015) trial record had not yet been published by May 2020, as far as we are aware, and as stated in the first publication of that study (Luby et al., 2018). Hence, Colford (2015) remains included in the map as an ongoing study, rather than as a completed study.

15 We refer to studies for simplicity, although all of the frequencies reported in this section refer to study arms.

16 It should be noted that searches were done by intervention and outcome categories, and it is therefore possible that such studies were missed.

17 The studies were identified in Waddington et al. (2009).

18 The figure suggests an apparent decline in production post‐2018, which is a function of the limited searches done since March 2018 (the estimated production in 2018–2020 therefore being likely underestimated).

19 The figure suggests an apparent decline in production post‐2018, which is a function of the limited searches done since March 2018 (the estimated production in 2018–2020 therefore being likely underestimated).

20 Blum and Feachem's (1983) review was done for diarrhoea morbidity research, but the areas are also relevant for other outcomes.

21 Barreto et al. (2007) was presented as a before‐after prospective cohort but due to the time‐series data collected, may be better defined as ITS, a credible quasi‐experimental design which should be included in high quality systematic reviews of effects (Bärnighausen et al., 2017; Reeves et al., 2017; Waddington et al., 2017).

22 There may also be instances of “discourtesy” bias arising in the collection of self‐reported repeated observation data (see Schmidt & Cairncross, 2009).

23 A separate Cochrane group, Evidence Aid, exists to coordinate humanitarian evidence.

24 For example, an Indian NGO, *Banka BioLoo* which provides “sustainable sanitation across India” had written to the Campbell Collaboration in appreciation of the De Buck et al. (2017) mixed‐methods review.

## ABBREVIATIONS

3ie: International Initiative for Impact Evaluation
BCC: behaviour change communication
BMGF: Bill & Melinda Gates Foundation
BMI: body mass index
CDD: community‐driven development
CLTS: community‐led total sanitation
CLTSH: community‐led total sanitation and hygiene
COVID‐19: coronavirus disease 2019
DAC: Development Assistance Committee
DALY: disability‐adjusted life year
DCD: Department of Disease Control
EAP: East Asia and Pacific
EGM: evidence and gap map
FCDO: Foreign, Commonwealth and Development Office
HAZ: height‐for‐age z‐score
HIV: human immunodeficiency virus
IEC: information and education communication
IGWG: Interagency Gender Working Group
JICA: Japan International Cooperation Agency
JMP: Joint Monitoring Programme
LAC: Latin America and the Caribbean
LMICs: low‐ and middle‐income countries
LSHTM: London School of Hygiene and Tropical Medicine
MDG: Millennium Development Goal
MENA: Middle East and North Africa
MHM: menstrual hygiene management
NGO: nongovernmental organisation
NRS: nonrandomised study
NTD: neglected tropical disease
ODF: open defecation free
PEM: protein energy management
PICOS: populations, interventions, comparators, outcomes, and study designs
POU: point‐of‐use
PRISMA: preferred reporting items for systematic reviews and meta‐analyses
PSM: propensity score matching
RCT: randomised controlled trial
RDD: regression discontinuity design
SDG: Sustainable Development Goal
SHARE: Sanitation and Hygiene Applied Research for Equity
UN: United Nations
UNICEF: United Nations Children's Fund
WASH: water, sanitation, and hygiene
WAZ: weight‐for‐age z‐score
WHO: World Health Organization
WHZ: weight‐for‐height z‐score
WSSCC: Water Supply and Sanitation Collaborative Council
WUA: water user association
