# Supplementary material for: Interventions promoting uptake of water, sanitation and hygiene (WASH) technologies in low‐ and middle‐income countries: An evidence and gap map of effectiveness studies
Source: Campbell Syst Rev. 2021 Oct 8;17(4):e1194. doi: 10.1002/cl2.1194 (PMC8988822; doi:10.1002/cl2.1194)
Supplement: Supplementary file 1 — Supporting information. [file CL2-17-e1194-s003.docx]

# Online supplements

*List of online supplements*

1. Data appendix: summary information on systematic reviews
2. Data appendix: summary information on impact evaluations

# Appendix A: Search details

**List of academic and trials registries searched**

| Database | Last date the database was searched |
| --- | --- |
| CAB Abstracts (Ovid) | 25 February 2018 |
| CAB Global Health (Ovid) | 23 February 2018 |
| Cochrane Library | 26 February 2018 |
| Econlit (Ovid) | 26 February 2018 |
| Embase (Ovid) | 23 February 2018 |
| ERIC (Ovid) | 27 February 2018 |
| Medline (Ovid) | 22 February 2018 |
| Popline | 28 February 2018 |
| Proquest Social Sciences Collection | 28 February 2018 |
| Social Science Citation Index (Web of Science) | 27 February 2018 |
| WHO Global Health Library | 01 March 2018 |
| American Economic Association RCT Registry | 07 March 2018 |
| Eldis Trials Registry | 06 February 2018 |
| OpenTrials | 06 February 2018 |
| WHO International Clinical Trials Registry Platform | 07 March 2018 |

**Example search string**

Below are example search strings formatted for Ovid MEDLINE(R). The first is for the update to the 2014 WASH evidence map searches, the second for the update to the De Buck et al. (2017) systematic review searches, and the third for the expanded scope to water behaviours and health facilities.

1 Developing Countries.sh,kf. (79987)

2 Africa/ or Asia/ or Caribbean/ or West Indies/ or South America/ or Latin America/ or Central America/ (69677)

3 (Africa or Asia or Caribbean or West Indies or South America or Latin America or Central America).tw. (151966)

4 (Afghanistan or Albania or Algeria or Angola or Argentina or Armenia or Armenian or Azerbaijan or Bangladesh or Benin or Byelarus or Byelorussian or Belarus or Belorussian or Belorussia or Belize or Bhutan or Bolivia or Bosnia or Herzegovina or Hercegovina or Botswana or Brazil or Bulgaria or Burkina Faso or Burkina Fasso or Upper Volta or Burundi or Urundi or Cambodia or Khmer Republic or Kampuchea or Cameroon or Cameroons or Cameron or Camerons or Cape Verde or Central African Republic or Chad or China or Colombia or Comoros or Comoro Islands or Comores or Mayotte or Congo or Zaire or Costa Rica or Cote d'Ivoire or Ivory Coast or Cuba or Djibouti or French Somaliland or Dominica or Dominican Republic or East Timor or East Timur or Timor Leste or Ecuador or Egypt or United Arab Republic or El Salvador or Eritrea or Ethiopia or Fiji or Gabon or Gabonese Republic or Gambia or Gaza or Georgia Republic or Georgian Republic or Ghana or Grenada or Guatemala or Guinea or Guiana or Guyana or Haiti or Honduras or India or Maldives or Indonesia or Iran or Iraq or Jamaica or Jordan or Kazakhstan or Kazakh or Kenya or Kiribati or Korea or Kosovo or Kyrgyzstan or Kirghizia or Kyrgyz Republic or Kirghiz or Kirgizstan or Lao PDR or Laos or Lebanon or Lesotho or Basutoland or Liberia or Libya or Macedonia or Madagascar or Malagasy Republic or Malaysia or Malaya or Malay or Sabah or Sarawak or Malawi or Mali or Marshall Islands or Mauritania or Mauritius or Agalega Islands or Mexico or Micronesia or Middle East or Moldova or Moldovia or Moldovian or Mongolia or Montenegro or Morocco or Ifni or Mozambique or Myanmar or Myanma or Burma or Namibia or Nepal or Netherlands Antilles or Nicaragua or Niger or Nigeria or Muscat or Pakistan or Palau or Palestine or Panama or Paraguay or Peru or Philippines or Philipines or Phillipines or Phillippines or Papua New Guinea or Romania or Rumania or Roumania or Rwanda or Ruanda or Saint Lucia or St Lucia or Saint Vincent or St Vincent or Grenadines or Samoa or Samoan Islands or Navigator Island or Navigator Islands or Sao Tome or Senegal or Serbia or Montenegro or Seychelles or Sierra Leone or Sri Lanka or Solomon Islands or Somalia or Sudan or Suriname or Surinam or Swaziland or South Africa or Syria or Tajikistan or Tadzhikistan or Tadjikistan or Tadzhik or Tanzania or Thailand or Togo or Togolese Republic or Tonga or Tunisia or Turkey or Turkmenistan or Turkmen or Uganda or Ukraine or Uzbekistan or Uzbek or Vanuatu or New Hebrides or Venezuela or Vietnam or Viet Nam or West Bank or Yemen or Zambia or Zimbabwe).tw,sh. (1179705)

5 ((developing or less* developed or under developed or underdeveloped or middle income or low* income or underserved or under served or deprived or poor*) adj (countr* or nation? or population? or world or state*)).ti,ab. (78902)

6 ((developing or less* developed or under developed or underdeveloped or middle income or low* income) adj (economy or economies)).ti,ab. (400)

7 (low* adj (gdp or gnp or gross domestic or gross national)).tw. (205)

8 (low adj3 middle adj3 countr*).tw. (8913)

9 (lmic or lmics or third world or lami countr*).tw. (5007)

10 transitional countr*.tw. (139)

11 or/1-10 (1323187)

12 (sanitation or sewage or sewerage or wastewater or domestic water or (water adj2 (access* or suppl* or quality or quantit* or standard* or drinking)) or hygiene).ti,ab. (161588)

13 Sanitation/ (6507)

14 water/ or drinking water/ or water supply/ or water quality/ or fresh water/ (198561)

15 waste water/ or sewage/ (35209)

16 or/12-15 (343517)

17 hand hygiene/ or hand disinfection/ (5814)

18 (handwash* or soap* or (hand* adj3 (wash* or hygiene or clean*))).ti,ab. (12639)

19 or/17-18 (14788)

20 respiratory tract infections/ or whooping cough/ (43029)

21 Sinusitis/ (15930)

22 Common Cold/ (4075)

23 Otitis Media/ (16557)

24 Pharyngitis/ (7508)

25 Influenza, Human/ (43747)

26 laryngitis/ or croup/ (3881)

27 Epiglottitis/ (947)

28 pneumonia/ or bronchopneumonia/ or pleuropneumonia/ or pneumonia, bacterial/ or pneumonia, pneumococcal/ or pneumonia, staphylococcal/ or pneumonia, viral/ (66073)

29 bronchitis/ or bronchiolitis/ (22955)

30 (ARIs or (respiratory adj (disease* or infection* or illness*)) or sinusitis or common cold* or otitis media or pharyngitis or influenza or flu or coryza or laryngitis or epiglottitis or croup or pneumonia or bronchitis or bronchiolitis or pertussis or whooping cough).ti,ab. (304365)

31 or/20-30 (385850)

32 11 and 19 and 31 (205)

33 (diarrh* or dysenter* or gastroenteritis or cholera* or "waterborne infection*" or enterotoxi* or enteric or enteritis or "escherichia coli*" or "e coli" or rotavirus* or mortality or death*).ti,ab. (1570626)

34 diarrhoea/ or dysentery/ or enteritis/ or gastroenteritis/ or cholera/ or vibrio cholerae/ or waterborne diseases/ or enterotoxins/ or exp escherichia coli/ or escherichia coli infections/ or rotavirus/ or exp mortality/ or "causes of death"/ or death/ or fatal infections/ (671938)

35 or/33-34 (1845583)

36 11 and 16 and 35 (6244)

37 ((time adj3 (saving* or allocat* or consum* or fetch* or travel*)) or (collect* adj3 (time* or behavio* or chore* or errand* or drudgery or burden* or inconvenien*))).ti,ab. (65859)

38 Time Factors/ (1109957)

39 37 or 38 (1169839)

40 11 and 16 and 39 (1713)

41 joint diseases/ or arthritis/ or exp musculoskeletal pain/ or back pain/ or low back pain/ or neck pain/ (97943)

42 ((back adj3 (pain* or injur*)) or backpain or ((neck or spine or spinal) adj3 (pain or injur*)) or neckpain or musculoskeletal or (joint adj3 (pain or disease* or injur*)) or arthriti* or osteoarthriti*).ti,ab. (330866)

43 Amenorrhea/ or Menstruation/ or Menstruation Disturbances/ or Menstrual Cycle/ or Oligomenorrhea/ or Dysmenorrhea/ (43803)

44 (menstruat* or menses or menstrual or amenorrh* or dysmenorrh* or oligomenorrh*).ti,ab. (58762)

45 41 or 42 or 43 or 44 (453245)

46 11 and 16 and 45 (255)

47 ("quasi experiment*" or quasi-experiment* or "random* control* trial*" or "random* trial*" or RCT or (random* adj3 allocat*) or matching or "propensity score" or PSM or "regression discontinuity" or "discontinuous design" or RDD or "difference in difference*" or difference-in-difference* or "diff in diff" or DID or "case control" or cohort or "propensity weighted" or propensity-weighted or "interrupted time series" or (before adj5 after) or (pre adj5 post) or ((pretest or pre test) and (posttest or post test)) or "research synthesis" or "scoping review" or "rapid evidence assessment" or "systematic literature review" or "Systematic review" or "Meta-analy*" or Metaanaly* or "meta analy*" or "Control* evaluation" or "Control treatment" or "instrumental variable*" or heckman or IV or ((quantitative or "comparison group*" or counterfactual or "counter factual" or counter-factual or experiment*) adj3 (design or study or analysis)) or QED).ti,ab,kw. (3095201)

48 clinical trial/ or clinical trial, phase i/ or clinical trial, phase ii/ or clinical trial, phase iii/ or clinical trial, phase iv/ or controlled clinical trial/ or randomized controlled trial/ or pragmatic clinical trial/ (784504)

49 controlled clinical trials as topic/ or non-randomized controlled trials as topic/ or randomized controlled trials as topic/ or pragmatic clinical trials as topic/ or case-control studies/ or retrospective studies/ or controlled before-after studies/ or interrupted time series analysis/ or random allocation/ or cohort studies/ or follow-up studies/ or longitudinal studies/ or prospective studies/ or retrospective studies/ or propensity score/ (2075906)

50 47 or 48 or 49 (4824377)

51 32 or 36 or 40 or 46 (8083)

52 50 and 51 (1912)

1. limit 52 to yr="2014 -Current" (503)

**Hygiene and sanitation updated search**

1 (Hand* and (clean* or disinfect* or sterili* or soap or treat* or sanitiz*)).ti,ab. (117045)

2 Sanitation/ (6507)

3 ((Faeces or feces or fecal or faecal or defecat* or excrement* or "human waste" or "night soil" or excreta) and (Dispos* or Manag*)).ti,ab. (8304)

4 (latrine* or toilet* or sanitation or lavator* or "water closet*").ti,ab. (14098)

5 Hand Hygiene/ or *Hygiene/ (9211)

6 ("Hand washing" or handwashing or hand-washing or "hand hygiene" or ((hand or hands) and wash*)).ti,ab. (9217)

7 Education/ or Health Knowledge, Attitudes, Practice/ or health promotion/ or life style/ or consumer participation/ or social marketing/ or Health behavior/ or Motivation/ or Decision making/ or Hygiene/education or Information Dissemination/ (391955)

8 ("change agent*" or "transformation agent*" or "hygiene promotor*" or "community leader*" or song* or "radio spot" or "radio program*" or megaphone or "focus group*" or cinema* or theatr* or television or TV or play* or "hygiene day*" or sticker* or poster* or billboard* or painting* or "home visit*" or "mass media" or disgust).ti,ab. (1327990)

9 (market* or market-based or "product design" or "supply side improvements" or incentiv* or subsidy or subsidies or voucher* or "cash transfer*" or microcredit or micro-credit* or loan* or financ* or advocacy or advocat*).ti,ab. (255239)

10 (community-based or participation or participatory or "Community Led Total Sanitation" or CLTS or "Participatory Rural Appraisal" or "Participatory Hygiene and Sanitation Transformation" or SARAR or "community reunion*" or "hygiene club*" or "mother club*" or "mothers club*" or "health club*" or "child-to-child" or "Urban Led Total Sanitation" or "community approach*" or "Community Action Planning" or "model home").ti,ab. (170478)

11 (Educat* or train* or lectur* or workshop* or game* or demonstrat* or quiz* or IBM-WASH or RANAS).ti,ab. (913920)

12 (Promot* or facilitat* or motivat* or encourag* or advoca* or persua* or sustain* or behaviour* or behavior* or habit* or custom* or tendency or packag* or program* or campaign*).ti,ab. (3350518)

13 1 or 2 or 3 or 4 or 5 or 6 (154433)

14 7 or 8 or 9 or 10 or 11 or 12 (5236519)

15 (afghanistan or albania or algeria or angola or antigua or barbuda or argentina or armenia or aruba or azerbaijan or bahrain or bangladesh or barbados or benin or byelarus or byelorussian or belarus or belorussian or belorussia or belize or bhutan or bolivia or bosnia or herzegovina or hercegovina or botswana or brazil or bulgaria or "Burkina Faso" or "Burkina Fasso" or "Upper Volta" or burundi or urundi or cambodia or "Khmer Republic" or kampuchea or cameroon or cameroons or cameron or cameron or "Cape Verde" or "Central African Republic" or chad or chile or china or colombia or comoros or "Comoro Islands" or comores or mayotte or congo or zaire or "Costa Rica" or "Cote d'Ivoire" or "Ivory Coast" or croatia or cuba or cyprus).ti,ab. (288175)

16 Asia, Central/ or Asia, Western/ or Asia, Southeastern/ (9399)

17 africa/ (27361)

18 South America/ (8986)

19 Caribbean Region/ (3813)

20 (Romania or Rumania or Roumania or Rwanda or Ruanda or "Saint Kitts" or "St Kitts" or Nevis or "Saint Lucia" or "St Lucia" or "Saint Vincent" or "St Vincent" or Grenadines or Samoa or "Samoan Islands" or "Navigator Island" or "Navigator Islands" or "Sao Tome" or "Saudi Arabia" or Senegal or Serbia or Seychelles or "Sierra Leone" or Slovenia or "Sri Lanka" or Ceylon or "Solomon Islands" or Somalia or Sudan or Suriname or Surinam or Swaziland or Syria or Tajikistan or Tadzhikistan or Tadjikistan or Tadzhik or Tanzania or Thailand or Togo or "Togolese Republic" or Tonga or Trinidad or Tobago or Tunisia or Turkey or Turkmenistan or Turkmen or Uganda or Ukraine or Uzbekistan or Uzbek or Vanuatu or "New Hebrides" or Venezuela or Vietnam or "Viet Nam" or "West Bank" or Yemen or Zambia or Zimbabwe).ti,ab. (155879)

21 (Macedonia or Madagascar or "Malagasy Republic" or Malaysia or Malaya or Malay or Sabah or Sarawak or Malawi or Nyasaland or Mali or Malta or "Marshall Islands" or Mauritania or Mauritius or "Agalega Islands" or Mexico or Micronesia or "Middle East" or Moldova or Moldovia or Moldovian or Mongolia or Montenegro or Morocco or Ifni or Mozambique or Myanmar or Myanma or Burma or Namibia or Nepal or "Netherlands Antilles" or "New Caledonia" or Nicaragua or Niger or Nigeria or "Northern Mariana Islands" or Oman or Muscat or Pakistan or Palau or Palestine or Panama or Paraguay or Peru or Philippines or Philipines or Phillipines or Phillippines or "Puerto Rico").ti,ab. (164748)

22 (asia or africa or "south america" or oceania or "latin america").ti,ab. (140257)

23 (Djibouti or "French Somaliland" or Dominica or "Dominican Republic" or "East Timor" or "East Timur" or "Timor Leste" or Ecuador or Egypt or "United Arab Republic" or "El Salvador" or Eritrea or Ethiopia or Fiji or Gabon or "Gabonese Republic" or Gambia or Gaza or Georgia or Georgian or Ghana or "Gold Coast" or Greece or Grenada or Guatemala or Guinea or Guam or Guiana or Guyana or Haiti or Honduras or Hungary or India or Maldives or Indonesia or Iran or Iraq or "Isle of Man" or Jamaica or Jordan or Kazakhstan or Kazakh or Kenya or Kiribati or Korea or Kosovo or Kyrgyzstan or Kirghizia or "Kyrgyz Republic" or Kirghiz or Kirgizstan or "Lao PDR" or Laos or Lebanon or Lesotho or Basutoland or Liberia or Libya).ti,ab. (357812)

24 developing countries/ (69189)

25 ((developing or "less* developed" or "under developed" or underdeveloped or "middle income" or "low* income" or underserved or deprived or poor*) and (countr* or nation* or population*)).ti,ab. (248392)

26 ("low and middle income countries" or LMIC*).ti,ab. (7809)

27 15 or 16 or 17 or 18 or 19 or 20 or 21 or 22 or 23 or 24 or 25 or 26 (1205301)

28 ("quasi experiment*" or quasi-experiment* or "random* control* trial*" or "random* trial*" or RCT or (random* adj3 allocat*) or matching or "propensity score" or PSM or "regression discontinuity" or "discontinuous design" or RDD or "difference in difference*" or difference-in-difference* or "diff in diff" or DID or "case control" or cohort or "propensity weighted" or propensity-weighted or "interrupted time series" or (before adj5 after) or (pre adj5 post) or ((pretest or pre test) and (posttest or post test)) or "research synthesis" or "scoping review" or "rapid evidence assessment" or "systematic literature review" or "Systematic review" or "Meta-analy*" or Metaanaly* or "meta analy*" or "Control* evaluation" or "Control treatment" or "instrumental variable*" or heckman or IV or ((quantitative or "comparison group*" or counterfactual or "counter factual" or counter-factual or experiment*) adj3 (design or study or analysis)) or QED).ti,ab,kw. (3098351)

29 clinical trial/ or clinical trial, phase i/ or clinical trial, phase ii/ or clinical trial, phase iii/ or clinical trial, phase iv/ or controlled clinical trial/ or randomized controlled trial/ or pragmatic clinical trial/ (784839)

30 controlled clinical trials as topic/ or non-randomized controlled trials as topic/ or randomized controlled trials as topic/ or pragmatic clinical trials as topic/ or case-control studies/ or retrospective studies/ or controlled before-after studies/ or interrupted time series analysis/ or random allocation/ or cohort studies/ or follow-up studies/ or longitudinal studies/ or prospective studies/ or retrospective studies/ or propensity score/ (2077087)

31 28 or 29 or 30 (4828295)

32 13 and 14 and 27 and 31 (2324)

1. limit 32 to ed=20160325-20180124 (388)

**Water behaviour change and health facility search**

1 water/ or drinking water/ or water supply/ or water wells/ or water resources/ or water purification/ or fresh water/ (217069)

2 water pollution/ or water quality/ or water microbiology/ or disinfection/ (55764)

3 (water adj5 (purif* or treat* or improv* or clean or decontaminat* or filt* or consum* or suppl* or drink* or potable or quantity or distribut* or stor* or volum*)).ti,ab,kw. (114515)

4 (water adj3 (hygien* or pollut* or contaminat* or foul or untreated)).ti,ab,kw. (18051)

5 (water adj2 (access* or availab*)).ti,ab,kw. (5991)

6 (water adj5 (disinfect* or connect* or quality or handpump* or standpipe* or pip*)).ti,ab,kw. (24533)

7 or/1-6 (344598)

8 (promot* or facilitat* or motivat* or encourag* or advoca* or persua* or sustain* or behaviour* or behavior* or habit* or custom* or adher* or attitud* or tendency or packag* or program* or campaign*).ti,ab,kw. (3580823)

9 (educat* or train* or lectur* or workshop* or game* or demonstrat* or quiz* or IBM-WASH or "Integrated Behavioural Model" or "integrated behavioral model" or RANAS).ti,ab,kw. (3177696)

10 (community-based or participation or participatory or "Community Led Total Sanitation" or CLTS or "Participatory Rural Appraisal" or "Participatory Hygiene and Sanitation Transformation" or SARAR or "community reunion*" or "hygiene club*" or "mother* club*" or "health club*" or "child-to-child" or "Urban Led Total Sanitation" or "community approach*" or "Community Action Planning" or "model home").ti,ab,kw. (172999)

11 (market* or market-based or "product design" or ("supply side" adj3 improv*) or incentiv* or subsid* or voucher* or "cash transfer*" or microcredit or micro-credit* or loan* or financ* or advocacy or advocat*).ti,ab,kw. (277756)

12 (((change or transform*) adj3 agent*) or (hygiene adj3 promot*) or "community leader*" or song* or "radio spot*" or "radio program*" or megaphone or "focus group*" or cinema* or theatr* or theater* or drama or television or TV or play* or "hygiene day*" or sticker* or poster* or billboard* or painting* or "home visit*" or "mass media" or disgust).ti,ab,kw. (1351491)

13 health education/ or health knowledge, attitudes, practice/ or health promotion/ or life style/ or social marketing/ or health behavior/ or motivation/ or decision making/ or hygiene/ed or information dissemination/ or persuasive communication/ (407565)

14 attitude to health/ or habits/ or health behavior/ or risk reduction behavior/ (130559)

15 or/8-14 (7114963)

16 Developing Countries.sh,kf. (80252)

17 Africa/ or Asia/ or Caribbean/ or West Indies/ or South America/ or Latin America/ or Central America/ (69930)

18 (Africa or Asia or Caribbean or West Indies or South America or Latin America or Central America).tw. (154177)

19 (Afghanistan or Albania or Algeria or Angola or Argentina or Armenia or Armenian or Azerbaijan or Bangladesh or Benin or Byelarus or Byelorussian or Belarus or Belorussian or Belorussia or Belize or Bhutan or Bolivia or Bosnia or Herzegovina or Hercegovina or Botswana or Brazil or Bulgaria or Burkina Faso or Burkina Fasso or Upper Volta or Burundi or Urundi or Cambodia or Khmer Republic or Kampuchea or Cameroon or Cameroons or Cameron or Camerons or Cape Verde or Central African Republic or Chad or China or Colombia or Comoros or Comoro Islands or Comores or Mayotte or Congo or Zaire or Costa Rica or Cote d'Ivoire or Ivory Coast or Cuba or Djibouti or French Somaliland or Dominica or Dominican Republic or East Timor or East Timur or Timor Leste or Ecuador or Egypt or United Arab Republic or El Salvador or Eritrea or Ethiopia or Fiji or Gabon or Gabonese Republic or Gambia or Gaza or Georgia Republic or Georgian Republic or Ghana or Grenada or Guatemala or Guinea or Guiana or Guyana or Haiti or Honduras or India or Maldives or Indonesia or Iran or Iraq or Jamaica or Jordan or Kazakhstan or Kazakh or Kenya or Kiribati or Korea or Kosovo or Kyrgyzstan or Kirghizia or Kyrgyz Republic or Kirghiz or Kirgizstan or Lao PDR or Laos or Lebanon or Lesotho or Basutoland or Liberia or Libya or Macedonia or Madagascar or Malagasy Republic or Malaysia or Malaya or Malay or Sabah or Sarawak or Malawi or Mali or Marshall Islands or Mauritania or Mauritius or Agalega Islands or Mexico or Micronesia or Middle East or Moldova or Moldovia or Moldovian or Mongolia or Montenegro or Morocco or Ifni or Mozambique or Myanmar or Myanma or Burma or Namibia or Nepal or Netherlands Antilles or Nicaragua or Niger or Nigeria or Muscat or Pakistan or Palau or Palestine or Panama or Paraguay or Peru or Philippines or Philipines or Phillipines or Phillippines or Papua New Guinea or Romania or Rumania or Roumania or Rwanda or Ruanda or Saint Lucia or St Lucia or Saint Vincent or St Vincent or Grenadines or Samoa or Samoan Islands or Navigator Island or Navigator Islands or Sao Tome or Senegal or Serbia or Montenegro or Seychelles or Sierra Leone or Sri Lanka or Solomon Islands or Somalia or Sudan or Suriname or Surinam or Swaziland or South Africa or Syria or Tajikistan or Tadzhikistan or Tadjikistan or Tadzhik or Tanzania or Thailand or Togo or Togolese Republic or Tonga or Tunisia or Turkey or Turkmenistan or Turkmen or Uganda or Ukraine or Uzbekistan or Uzbek or Vanuatu or New Hebrides or Venezuela or Vietnam or Viet Nam or West Bank or Yemen or Zambia or Zimbabwe).tw,sh. (1192478)

20 ((developing or less* developed or under developed or underdeveloped or middle income or low* income or underserved or under served or deprived or poor*) adj (countr* or nation? or population? or world or state*)).ti,ab. (80086)

21 ((developing or less* developed or under developed or underdeveloped or middle income or low* income) adj (economy or economies)).ti,ab. (407)

22 (low* adj (gdp or gnp or gross domestic or gross national)).tw. (208)

23 (low adj3 middle adj3 countr*).tw. (9293)

24 (lmic or lmics or third world or lami countr*).tw. (5120)

25 transitional countr*.tw. (139)

26 or/16-25 (1337623)

27 ("quasi experiment*" or quasi-experiment* or "random* control* trial*" or "random* trial*" or RCT or (random* adj3 allocat*) or matching or "propensity score" or PSM or "regression discontinuity" or "discontinuous design" or RDD or "difference in difference*" or difference-in-difference* or "diff in diff" or DID or "case control" or cohort or "propensity weighted" or propensity-weighted or "interrupted time series" or (before adj5 after) or (pre adj5 post) or ((pretest or pre test) and (posttest or post test)) or "research synthesis" or "scoping review" or "rapid evidence assessment" or "systematic literature review" or "Systematic review" or "Meta-analy*" or Metaanaly* or "meta analy*" or "Control* evaluation" or "Control treatment" or "instrumental variable*" or heckman or IV or ((quantitative or "comparison group*" or counterfactual or "counter factual" or counter-factual or experiment*) adj3 (design or study or analysis)) or QED).ti,ab,kw. (3136366)

28 clinical trial/ or clinical trial, phase i/ or clinical trial, phase ii/ or clinical trial, phase iii/ or clinical trial, phase iv/ or controlled clinical trial/ or randomized controlled trial/ or pragmatic clinical trial/ (787933)

29 controlled clinical trials as topic/ or non-randomized controlled trials as topic/ or randomized controlled trials as topic/ or pragmatic clinical trials as topic/ or case-control studies/ or retrospective studies/ or controlled before-after studies/ or interrupted time series analysis/ or random allocation/ or cohort studies/ or follow-up studies/ or longitudinal studies/ or prospective studies/ or retrospective studies/ or propensity score/ (2088788)

30 or/27-29 (4873550)

31 7 and 15 and 26 and 30 (2449)

32 limit 31 to yr="2000 -Current" (**2019**)

33 water/ or drinking water/ or water supply/ or water wells/ or water resources/ or water purification/ or fresh water/ (217069)

34 sanitation/ (6534)

35 hygiene/ or hand hygiene/ or hand disinfection/ (20243)

36 waste water/ or sewage/ or waste management/ or waste disposal facilities/ (42024)

37 water pollution/ or water quality/ or water microbiology/ or disinfection/ (55764)

38 (water adj5 (purif* or treat* or improv* or clean or decontaminat* or filt* or consum* or suppl* or drink* or potable or quantity or distribut* or stor* or volum*)).ti,ab,kw. (114515)

39 (water adj3 (hygien* or pollut* or contaminat* or foul or untreated)).ti,ab,kw. (18051)

40 (water adj2 (access* or availab*)).ti,ab,kw. (5991)

41 (hand* and (clean* or disinfect* or sterili* or soap or treat* or saniti*)).ti,ab,kw. (118850)

42 ("hand washing" or handwashing or hand-washing or "hand hygiene" or ((hand or hands) adj3 wash*)).ti,ab,kw. (7516)

43 (latrine* or toilet* or sanitation or lavator* or "water closet*" or wc or privy or "pour flush").ti,ab,kw. (22015)

44 toilet facilities/ (1393)

45 ((faeces or feces or fecal or faecal or defecat* or excrement* or "human waste" or "night soil" or excreta) adj3 (dispos* or manag* or service*)).ti,ab,kw. (868)

46 (water adj5 (disinfect* or connect* or quality or handpump* or standpipe* or pip*)).ti,ab,kw. (24533)

47 or/33-46 (523851)

48 Maternal Mortality/ or Pregnancy Outcome/ (52893)

49 exp Pregnancy Complications/ep, mo, pc [Epidemiology, Mortality, Prevention & Control] (76375)

50 exp Female Urogenital Diseases/ep, mo, pc [Epidemiology, Mortality, Prevention & Control] (166468)

51 exp Infant, Newborn, Diseases/ep, mo, pc [Epidemiology, Mortality, Prevention & Control] (26103)

52 child mortality/ or fetal mortality/ or perinatal mortality/ or sudden infant death/ or infant death/ or infant mortality/ (36723)

53 ((pregnan* or obstetric* or labour or labor or maternal or mother* or child* or fetal or foetal or newborn* or neonat* or infant* or perinatal or antenatal) adj3 (mortality or death* or outcome* or morbidity)).ti,ab,kw. (156511)

54 nutrition disorders/ or child nutrition disorders/ or infant nutrition disorders/ or vitamin k deficiency bleeding/ or malnutrition/ or exp deficiency diseases/ or exp avitaminosis/ or exp protein deficiency/ or swayback/ or fetal nutrition disorders/ or refeeding syndrome/ or severe acute malnutrition/ or kwashiorkor/ or starvation/ (133576)

55 body mass index/ or skinfold thickness/ or anthropometry/ or nutrition assessment/ or nutritional status/ or fetal growth retardation/ (193296)

56 ((nutrition* or malnutrition or malnourish* or underweight or stunted or kwashiorkor or marasmus or vitamin* or avitaminosis or deficien* or starvation or body mass or skinfold or anthropometr* or height-for-age or HAZ or weight-for-age or WAZ) adj3 (infant* or child* or newborn* or neonat* or perinatal or postnatal or post natal or post-natal)).ti,ab,kw. (38240)

57 or/49-56 (712844)

58 exp Health Facilities/ (713149)

59 inpatients/ or outpatients/ (29204)

60 (hospital* or clinic or clinics or health center* or health centre* or medical centre* or medical center* or health facilit* or medical facilit* or birth* center* or birth* centre* or inpatient* or outpatient*).ti,ab,kw. (1441795)

61 or/58-60 (1844264)

62 26 and 30 and 47 and 57 and 61 (329)

63 limit 62 to yr="2000 -Current" (**235**)

64 32 or 63 (**2196**)

**Organization websites and repositories hand-searched**

| Database | Website | Last date the database was searched |
| --- | --- | --- |
| 3ie | [3ie water and sanitation sector](http://www.3ieimpact.org/en/evidence/impact-evaluations/?sector=Water+and+Sanitation&sector=Water+Supply+and+Sanitation+Reform&sector=Urban+Water+and+Sanitation&sector=Rural+Water+and+Sanitation&q=&all=on&sort_by=alphabet) | 15 May 2018 |
| Abdul Latif Jameel Poverty Action Lab (J-PAL) | [J-PAL evaluations](https://www.povertyactionlab.org/evaluations?f%5b0%5d=field_intervention_type:542) | 15 May 2018 |
| African Development Bank | [African Development Bank evaluation reports](https://www.afdb.org/en/documents/evaluation-reports/#c) | 15 May 2018 |
| Asian development Bank | [ADB Impact evaluation studies](https://www.adb.org/search?page=1&facet_query=ola_collection_name%3Aevaluation_document&facet_query=sm_field_series_names%3AImpact%20Evaluation%20Studies) | 15 May 2018 |
| CEGA (University of California Center for Effective Global Action) | [CEGA water and sanitation research projects](http://cega.berkeley.edu/evidence/?filter=&filter-region=&filter-topic=subtopic-30&filter-researcher=&filter-methodology=&filter-text=) | 15 May 2018 |
| Department for International Development Research for Development | [Research for Development outputs in water and sanitation](https://www.gov.uk/dfid-research-outputs?keywords=&dfid_theme%5B%5D=water_and_sanitation&first_published_at%5Bfrom%5D=2014&first_published_at%5Bto%5D=) | 14 May 2018 |
| IMPROVE International | [WASH organisations with independent evaluations](http://improveinternational.wordpress.com/handy-resources/independent-evaluations/) | 14 May 2018 |
| IRC (WASH) | [Water and Sanitation Impact Evaluations](http://www.washdoc.info/docsearch/results?lmt=20&txt=water+sanitation+impact+evaluation&combine=all&field=&language=&mediatype=&dateset=since&date=0) | 14 May 2018 |
| Innovations for Poverty Action (IPA) | [IPA WASH projects](https://www.poverty-action.org/search-studies?tid_1%5B%5D=2056&field_status_value=All&title=) | 08 May 2018 |
| Inter-American Development Bank | [Office of Evaluation and Oversight: project and impact evaluations in water & sanitation](https://www.iadb.org/en/evaluation) | 04 May 2018 |
| Oxfam | [Water sanitation and hygiene evaluation and research reports](https://policy-practice.oxfam.org.uk/publications/search?i=1;q=*;q1=publications;q2=Water+Sanitation+and+Hygiene;q3=Evaluation+report\|Journal+article\|Research+report;show_all=prof;sort=publication_date;x1=page_type;x2=subject_area;x3=publication_type) | 04 May 2018 |
| USAID | [USAID development experience clearinghouse](https://dec.usaid.gov/dec/search/SearchResults.aspx?q=ZG9jdW1lbnRzLndlYl9jb2xsZWN0aW9uOigiRk9SV0FSRCBldmFsdWF0aW9uIik=&ctID=ODVhZjk4NWQtM2YyMi00YjRmLTkxNjktZTcxMjM2NDBmY2Uy&svn=ODVhZjk4NWQtM2YyMi00YjRmLTkxNjktZTcxMjM2NDBmY2UyIXZpZXdJRF83MTk3Zjk4My04MmRlLT) | 04 May 2018 |
| UNICEF | [UNICEF evaluation database](https://www.unicef.org/evaldatabase/index_23816.html) | 09 May 2018 |
| World Bank Development Impact Evaluation (DIME) | [DIME](http://documents.worldbank.org/curated/en/docsearch/collection-title/Impact%2520Evaluation%2520series?colT=Impact%2520Evaluation%2520series) | 14 May 2018 |
| World Bank Independent Evaluation Group (IEG) | [IEG Systematic reviews and impact evaluations](https://ieg.worldbankgroup.org/ieg-search?field_report_type_tags_1=1962&search_api_fulltext=&field_topic=All&field_report_type_tags%5B%5D=931&type_1%5B%5D=evaluation&type_1%5B%5D=reports&content_type_1=evaluation-reports&field_sub_category=All&field_organization_tags=All&type_2_op=not&type_2%5B%5D=expert&sort_by=field_official_date&sort_order=DESC) | 04 May 2018 |

# Appendix B: Data extraction form

| **Number** | **ID** | **Description** | **Question** | **Coding** |
| --- | --- | --- | --- | --- |
| 1. ***Publication details*** | | | | |
| 1.01 | STUDY ID | Unique study identifier |  | WEGM### |
| 1.02 | AUTHOR | First author |  | Surname |
| 1.03 | DATE | Publication year |  | YYYY |
| 1.04 | COUNTRY | Country/ies of study |  | Open answer |
| 1.05 | TITLE | Full title of study |  | Open answer |
| 1.06 | SHORT | Short title of study |  | Country (author, year) |
| 1.07 | REGION | Global region of study |  | East Asia & Pacific (EAP) Latin America & Caribbean (LAC) Middle East & North Africa (MENA) South Asia (SA) Sub-Saharan Africa (SSA) |
| 1.08 | FIELD | Academic field | Indicate the academic field of the study publication or authors | Environmental health  Social science |
| 1. ***Intervention details*** | | | | |
| 2.01 | INTERVENTION | Intervention mechanism used to provide WASH technology | Indicate the mechanism by which the above technology was supplied to the individual/ household/ facility/ community | **Demand:**  Health education: mass media  Health education: household/community  Health education: training of trainers (incl. teachers, community leaders, medics)  Health education): combined approach  Psychosocial 'triggering': directive (incl. social marketing)  Psychosocial 'triggering: participative (incl. CLTS)  Subsidies and microloans for consumers  Legal measures  **Supply:**  Direct provision of technology  Improving operator performance (incl. institutional reform, capacity building, operator financing, regulation, and accountability)  Privatisation and nationalisation  Small-scale independent provider (SSIP) involvement (incl. contracting out, sanitation marketing)  **Demand and supply:**  Community based approaches (incl. community driven development)  Multiple mechanisms |
| 2.02 | TECHNOLOGY | WASH technology received by participants | Indicate type of WASH technology received | Water supply: community provision  Water supply: household provision  Water supply: at school  Water supply: at health facility  Water treatment: community provision  Water treatment: household provision  Water treatment: at school  Water treatment: at health facility  Sanitation: latrines for communal use  Sanitation: latrines for household use  Sanitation: latrines at school  Sanitation: latrines at health facility  Sanitation: safe waste disposal system (e.g., sewer connection/drainage system)  Hygiene: improved handwashing practices  Hygiene: handwashing supplies (e.g., soap or hand sanitiser) for household  Hygiene: handwashing supplies (e.g., soap or hand sanitiser) at school  Hygiene: handwashing supplies (e.g., soap or hand sanitiser) at health facility  Hygiene: menstrual care  Hygiene: other improved practices (incl. face washing)  Hygiene: combined technologies  Combined water supply and sanitation (WSS)  Combined hygiene with water and/or sanitation |
| 2.03 | RURAL | Rural setting | Is study conducted in a rural area? | Yes/No |
| 2.04 | URBAN | Urban setting | Is study conducted in an urban or peri-urban (end of urban) area? | Yes/No |
| 2.05 | INFORMAL | Informal (peri-urban) setting | Is study conducted in a slum (informal settlement)? | Yes/No |
| 2.06 | REFUGEE | Refugee camp setting | Is study conducted in a refugee camp? | Yes/No |
| 2.07 | TARGET_GROUP | Intervention targeting | Which population groups did the intervention target? | Open answer (e.g., women, children, elderly, disabled, people living with HIV/AIDS) |
| 1. ***Study design and outcomes data*** | | | | |
| 3.01 | DESIGN | Design type | Categorise the study design | Individually randomised controlled trial (RCT) Cluster-RCT  Non-randomised trial Before-after study Retrospective non-randomised study  Case-control study  Natural experiment |
| 3.02 | WELLBEING | Quality of life outcome | What outcomes are reported (code multiple outcomes in additional columns)? | Mortality  Ill-health: diarrhoea Ill-health: acute respiratory infection Ill-health: other water-related disease (e.g., helminth infection, trachoma, malaria, arsenicosis) Ill-health: drudgery, pain, musculoskeletal disorders  Psycho-social health: stress, safety and vulnerability Nutrition: HAZ, WAZ, WHZ, BMI, anaemia Education and cognitive development: absenteeism, test scores Labour market and employment Income/consumption/poverty including health care and aversion costs |
| 3.03 | BEHAVIOUR | Behavioural outcome | What outcomes are reported (code multiple outcomes in additional columns)? | Water supply behaviour: quantity consumed/used  Water supply behaviour: source quality Water treatment and storage practices Sanitation behaviour: construction, use, and maintenance of latrines Sanitation behaviour: open defecation practices Hygiene behaviour Time use Willingness to pay  Sustainability and slippage  Political engagement |
| 3.04 | SEX | Sex disaggregation of outcomes data | Were sex disaggregated results reported for the outcomes? | Yes/No |
| 1. ***Critical appraisal data collection*** | | | | |
| 4.01 | CONFOUNDING | Methods used to address confounding | Was confounding addressed through use of a control group and/or adjusted analysis? | Yes/No |
| 4.02 | OUTCOME DEFINITION | definition of the outcome | Was the outcome clearly defined? | Yes/No |
| 4.03 | RECALL | Length of recall | For reported outcomes, what was the recall period? | Number of days |
| 4.04 | TECHNOLOGY USE | Analysis of use | Were data collected on use of WASH technology? | Yes/No |
| 4.05 | NUM OBSERVATIONS | Individual sample size | Total number of participants (including control, if relevant) | Open answer |
| 4.06 | NUM GROUPS | Cluster sample size | Total number of clusters (including control, if relevant) | Open answer |
| 4.07 | PARTICIPANT FLOW | Participant flow diagram | For prospective studies, is a participant flow diagram reported (or sufficient information to reconstruct it)? | YES/NO |
| 4.08 | ETHICS | Institutional review board (IRB) | Indicate the names of any IRB | Open answer |

# Appendix C: Critical appraisal tool

| Systematic review identifying information (author, year): |
| --- |
| Provide a full reference for all articles and reports used to complete this tool (e.g., systematic review technical report, systematic review protocol, systematic review summary report, journal article): |
| Date of the last search for studies in review: |

**Part 1: Critical appraisal of systematic review conduct and reporting**

***Section A:* *Methods used to identify and include studies***

| A.1 Did the report contain an explicit statement about a study protocol and any deviations from it?  🞏 Do the authors refer to a published protocol?  🞏 Do the authors indicate that a protocol or guide was produced but this is not publicly available?   Do the authors indicate any deviations from protocol explicitly (even if just to say that ‘there were no deviations from protocol’) | 🞏 Yes  🞏 Partially  🞏 No  *Coding guide:*  *YES: The authors refer to a published protocol and report any deviations from protocol.*  *PARTIALLY: A protocol/plan is mentioned but not formally referenced or available, or deviations from protocol indicated.*  *NO: All other.* |
| --- | --- |
| *Comments (note important limitations or uncertainty):* | |
| A.2 Were the criteria for deciding which studies to include in the review reported?  Did the authors specify:  🞏 Participants/ setting(s)/ population(s)?  🞏 Intervention(s)?  🞏 Outcome(s)  🞏 Study types included?  🞏 Other (specify)? | 🞏 Yes  🞏 Partially  🞏 No  *Coding guide - check the answers above*  *YES: PIOS should all be mentioned*  *NO: Any of P, I and O are not mentioned.*  *PARTIALLY: S is not mentioned.* |
| *Comments (note important limitations or uncertainty)* | |
| A.3 Was the search for evidence reasonably comprehensive?  Were the following done:  🞏 Language bias avoided (no stated restriction of inclusion based on language e.g., only English language studies are included)?  🞏 No restriction of inclusion based on publication status?  🞏 Relevant databases searched (Minimum criteria: All reviews should search at least one source of grey literature such as Google; for health: Medline/ Pubmed + Cochrane Library; for social sciences IDEAS + at least one database of general social science literature and one subject specific database)?  🞏 Reference lists in included articles checked?  🞏 Authors/experts contacted? | 🞏 Yes  🞏 Partially  🞏 No  *Coding guide - check the answers above:*  *YES: All five should be yes*  *PARTIALLY: Relevant databases and reference lists are both reported*  *NO: Any other* |
| *Comments (note important limitations or uncertainty)* | |
| A.4 Does the review cover an appropriate time period?  Is the search period comprehensive enough that relevant literature is unlikely to be omitted?  *Note: there may be important reasons for adopting different dates for the search, e.g., depending on the intervention. If you think there are limitations with the timeframe adopted for the search which have not been noted and justified by the authors, you should code this item as NO and specify your reason for doing so in the comment box below. Older reviews should not be downgraded. Always report the time period for the search in the comment box, as well as the date of latest search in the summary box on the first page.* | 🞏 Yes  🞏 Can't tell (only use if no information about time period for search)  🞏 No  🞏 Unclear  *Coding guide:*  *YES: Generally this means searching the literature at least back to 1990, but some reviews are able to argue convincingly why searches are more restricted (e.g., updates, evidence from related reviews)*  *NO: The search does not go at least back to 1990 and the review does not argue reasonably why restrictions are made*  *UNCLEAR: No information about time period for search* |
| *Comments (note search period, any justification provided for the search period, or uncertainty)* | |
| A.5 Was bias in the selection of articles avoided?  Did the authors specify:  🞏 Independent screening of full text by at least two reviewers?  🞏 List of included quantitative studies provided?  🞏 List of included qualitative studies provided, or indication of studies from which qualitative evidence collected (if relevant)?  🞏 List of quantitative studies excluded at full text provided?  🞏 List of qualitative studies excluded at full text provided (only relevant if separate searches undertaken for qualitative evidence)? | 🞏 Yes  🞏 Partially  🞏 No  *Coding guide:*  *YES: All four should be yes for reviews incorporating quantitative and qualitative evidence systematically For reviews that draw on qualitative evidence non-systematically, only three should be yes. For reviews published in journals with wordcount restrictions, supplementary files should be available on the journal website and/or the missing information contained in a report available online.*  *PARTIALLY: Independent screening and list of included studies provided are both reported*  *NO: All other.* |
| *Comments (note important limitations or uncertainty):* | |
| A.6 Were methods used to code studies in order to avoid incorporating dependent findings into any single analysis?  Did the authors specify:  🞏 Methods to address dependency in findings between studies (e.g., multiple publications of the same report, or on the same dataset)?  🞏 Methods to address dependency in findings within studies (e.g., multiple outcomes reported, or different specifications reported in analysis, or common control groups in multi-arm trials, or multiple follow-ups)? | 🞏 Yes  🞏 Partially  🞏 No  *Coding guide:*  *YES: both are ticked*  *PARTIALLY: one box ticked*  *NO: no boxes ticked.* |
| *Comments (note important limitations or uncertainty):* | |
| A. Overall – how much confidence do you have in the methods used to identify, include and critically appraise studies?  *Note: summary assessment score A relates to the 7 questions above.*  *High confidence applicable when the summary answer to the questions in section A are all assessed as ‘yes’.*  *Low confidence applicable when any of the following are assessed as ‘NO’ above: not reporting study protocol (A1), not reporting explicit selection criteria (A2), not conducting reasonably comprehensive search (A3), not avoiding bias in selection of articles (A5), and not reporting methods to address dependency in findings (A6)*  *Medium confidence applicable for any other.* | 🞏 Low confidence (limitations are important enough that the results of the review are not reliable)  🞏 Medium confidence (limitations are important enough that it would be worthwhile to search for another systematic review and to interpret the results of this review cautiously, if a better review cannot be found)  🞏 High confidence (only minor limitations) |
| *Comments (note important limitations).* | |

***Section B: Methods used to analyse the findings in the quantitative analysis***

| B.1 Were the characteristics and results of the included studies reliably reported?  Was there:  🞏 Independent data extraction by at least two reviewers?  🞏 A table or summary of the characteristics of the participants, interventions and outcomes for the included studies?  🞏 A table or summary of the results of all the included studies? | 🞏 Yes  🞏 No  🞏 Partially  🞏 Not applicable (e.g., no included studies)  *Coding guide:*  *YES: All three should be yes*  *PARTIALLY: Criteria one and three are yes, but some information is lacking on second criteria, or independent data extraction performed for only a sub-sample of studies.*  *No: None of these are reported. If the review does not report whether data was independently extracted by 2 reviewers (possibly a reporting error), downgrade to NO.*  *NOT APPLICABLE: if no studies/no data* |
| --- | --- |
| *Comments (note important limitations or uncertainty)* | |
| B.2 Did the authors use appropriate criteria to analyse the risk of bias in the studies that are included?  🞏 Were the criteria used for assessing risk of bias reported?  🞏 Was a table or summary of the assessment of each included study for each criterion reported?  🞏 Were criteria used that focus on the quality of study implementation/ risk of bias (and not other aspects of the studies, such as precision, construct validity or external validity)? “Sensible” is defined as a recognised quality appraisal tool/ checklist, or similar tool which critically appraises methodological implementation of included studies (not just design characteristics), such as control for confounding, selection bias, bias in outcomes data measurement and bias in reporting. | 🞏 Yes  🞏 Partially  🞏 No  *Coding guide:*  *YES: All three should be yes*  *PARTIALLY: The first and third criteria should be reported. If the authors report the criteria for assessing risk of bias and report a summary of this assessment for each criterion, but the criteria may be only partially sensible (e.g., do not address all possible risks of bias, but do address some), we downgrade to PARTIALLY.*  *NO: Any other* |
| *Comments (note important limitations or uncertainty)* | |
| B.3 Were the methods used by the review authors to analyse the findings of the included studies clear, including methods for calculating effect sizes? | 🞏 Yes  🞏 Partially  🞏 No  🞏 Not applicable (e.g., no studies or no data)  *Coding guide:*  *YES: Methods used clearly reported. If it is clear that the authors use narrative synthesis, they don't need to say this explicitly.*  *PARTIALLY: Some reporting on methods but lack of clarity*  *NO: Nothing reported on methods*  *NOT APPLICABLE: if no studies/no data* |
| *Comments (note important limitations or uncertainty)* | |
| B.4 Did the review describe the extent of heterogeneity?  🞏 Did the review discuss the extent to which there were important differences in the results of the included studies?  🞏 If a meta-analysis was done, was the I^2^ and other appropriate statistic reported (Tau^2^)? | 🞏 Yes  🞏 Partially  🞏 No  🞏 Not applicable (e.g., no studies or no data)  *Coding guide:*  *YES: First category should be yes, and second category should be yes if applicable*  *PARTIALLY: The first category is yes*  *NO: Any other*  *NOT APPLICABLE: if no studies/no data* |
| *Comments (note important limitations or uncertainty)* | |
| B.5 Were the findings of the relevant studies combined (or not combined) appropriately relative to the primary question the review addresses and the available data?  Was the data analysis done:  🞏 Descriptive only?  🞏 Vote counting based on direction of effect and/or statistical significance?  🞏 Description of range of effect sizes?  🞏 Statistical meta-analysis or meta-regression of effect sizes?  🞏 Other (specify)?  🞏 Not applicable (e.g., no studies or no data)?  Were the studies weighted in the analysis:  🞏 Equal weights (e.g., this is what is done when vote counting is used)?  🞏 By quality or study design (this is rarely done)?  🞏 Inverse variance (this is what is typically done in a meta-analysis)?  🞏 Number of participants (sample size)?  🞏 Other (specify)?  🞏 Unclear?  🞏 Not applicable (e.g., no studies or no data)?  Did the review address unit of analysis errors?  🞏 Yes - took clustering of participants within the study into account in the analysis (e.g., used intra-cluster correlation coefficient to calculate effect size standard errors)  🞏 No, but acknowledged problem of unit of analysis errors  🞏 No mention of issue  🞏 Not applicable - no clustered trials or studies included | 🞏 Yes  🞏 Partially  🞏 No  🞏 Not applicable (e.g., no studies or no data)  🞏 Unclear  *Coding guide:*  *YES: If analysis based on effect size data (e.g., statistical meta-analysis), appropriate weights and unit of analysis errors addressed (if appropriate).*  *PARTIALLY: If appropriate table, graph or meta-analysis and appropriate weights are used, but unit of analysis errors not addressed (and should have been).*  *NO: If vote counting is used where quantitative analyses would have been possible.*  *NOT APPLICABLE: if no studies/no data*  *UNCLEAR: if unsure (note reasons in comments below)* |
| *Comments (note important limitations or uncertainty)* | |
| B.6 Does the review report evidence appropriately?  🞏 The review makes clear which evidence is subject to low risk of bias in assessing causality (attribution of outcomes to intervention), and which is likely to be biased, and does so appropriately.  🞏 Where studies of differing risk of bias are included, results are reported and analysed separately by risk of bias status. | 🞏 Yes  🞏 No  🞏 Partially  🞏 Not applicable  *Coding guide:*  *YES: Both criteria should be fulfilled (where applicable)*  *NO: Criteria not fulfilled*  *PARTIALLY: Only one criteria fulfilled, or when there is limited reporting of quality appraisal (the latter applies only when inclusion criteria for study design are appropriate)*  *NOT APPLICABLE: No included studies*  *Note on reporting evidence and risk of bias: For reviews of effects of ‘large n’ interventions, experimental, quasi-experimental, natural experimental and observational designs should be included (if available). For reviews of effects of ‘small n’ interventions, designs appropriate to attribute changes to the intervention should be included (e.g., pre-post with assessment of confounders)* |
| *Please specify included study designs and any other comments (note important limitations or uncertainty):* | |
| B.7 Did the review examine the extent to which specific factors might explain differences in the results of the included studies?  🞏 Were factors that the review authors considered as likely explanatory factors clearly described pre-hoc (in the protocol) and reported as being identified in protocol?  🞏 Were any factors that the review authors considered as likely explanatory factors that were identified post-hoc (after the protocol) clearly indicated as such?  🞏 Was a sensible method used to explore the extent to which key factors explained heterogeneity?  🞏 Descriptive/textual  🞏 Graphical  🞏 Meta-analysis by moderators and sub-groups  🞏 Meta-regression  🞏 Other | 🞏 Yes  🞏 Partially  🞏 No  🞏 Not applicable  *Coding guide:*  *YES: Explanatory factors clearly described and appropriate methods used to explore heterogeneity*  *PARTIALLY: Explanatory factors described but for meta-analyses, sub-group analysis or meta-regression not reported (when they should have been)*  *NO: No description or analysis of likely explanatory factors*  *NOT APPLICABLE: e.g., too few studies, no important differences in the results of the included studies, or the included studies were so dissimilar that it would not make sense to explore heterogeneity of the results* |
| *Comments (note important limitations or uncertainty):* | |
| B. Overall - how much confidence do you have in the methods used to analyse the findings relative to the primary question addressed in the review?  *Note: summary assessment score B relates to the 8 questions in this section, regarding the analysis.*  *High confidence applicable when all the answers to the questions in section B are assessed as ‘yes’.*  *Low confidence applicable when any of the following are assessed as ‘NO’ above: critical characteristics of the included studies not reported (B1), not describing the extent of heterogeneity (B4), combining results inappropriately (B5), reporting evidence inappropriately (B6).*  *Medium confidence applicable for any other: i.e., the “Partial” option is used for any of the 6 preceding questions or questions and/or B.2 and/ or B.3 and/ or B.7 are assessed as ‘no’.* | 🞏 Low confidence (limitations are important enough that the results of the review are not reliable)  🞏 Medium confidence (limitations are important enough that it would be worthwhile to search for another systematic review and to interpret the results of this review cautiously, if a better review cannot be found)  🞏 High confidence (only minor limitations) |
| *Use comments to specify if relevant, to flag uncertainty or need for discussion* | |

**Part 2: Overall assessment and causal pathway analysis**

***Section C: Overall assessment of the confidence in the review***

| C.1 Are there any other aspects of the review not mentioned before which lead you to question the results? | 🞏 Additional methodological concerns – only one person reviewing  🞏 Robustness  🞏 Interpretation  🞏 Conflicts of interest (of the review authors or for included studies)  🞏 Other  🞏 No other quality issues identified |
| --- | --- |
| C.2 Are there any mitigating factors which should be taken into account in determining the reviews reliability? | 🞏 Limitations acknowledged  🞏 No strong policy conclusions drawn (including in abstract/ summary)  🞏 Any other factors |
| *Use comments to specify if relevant, to flag uncertainty or need for discussion* | |
| C.3 Based on the assessments in sections A and B of the methods, describe the confidence in the review findings.  🞏 Low confidence in conclusions about effects:  The systematic review has the following major limitations (if applicable)...  🞏 Medium confidence in conclusions about effects:  The systematic review has the following limitations (if applicable)...  🞏 High confidence in conclusions about effects:  The review has the following minor limitations (if applicable)...  Coding guide:  High confidence in conclusions about effects: high confidence noted overall for sections A and B, unless moderated by answer to C1.  Medium confidence in conclusions about effects: medium confidence noted overall for sections A or B, unless moderated by answer to C1 or C2.  Low confidence in conclusions about effects: low confidence noted overall for sections A or B, unless moderated by answer to C1 or C2. | |
| *Comments (explain why or note uncertainty)* | |

***Section D: Methods used to analyse the causal pathway and reach conclusions***

| D.1 Does the review use a programme theory?  Did the authors present:  🞏 A logic model (e.g., articulating the intervention causal chain from inputs/activities through to outcomes)?  🞏 Assumptions, either in the logic model itself or discussed in supporting text?  🞏 A (middle-range) theory (economic theory, e.g., consumer theory; social theory, e.g., diffusion theory; etc.) which informed the logic models and/or from which inferences can be made about mechanisms and contexts under which outcomes might occur? | 🞏 Yes  🞏 No  🞏 Partially  *Coding guide:*  *YES: Some theory is used, whether an intervention level logic model or causal chain, or formal theory, and underlying assumptions are explicitly described.*  *NO: None are reported.*  *PARTIALLY: A theory is used but underlying assumptions are not reported.* |
| --- | --- |
| *Comments (note important limitations or uncertainty)* | |
| D.2 Were review questions reported transparently (primary and secondary questions)?  Did the authors specify a separate review question for each review component:  🞏 Question(s) about intervention design and/or implementation processes such as inputs, activities and outputs (e.g., implementation fidelity)?  🞏 Question(s) about participant or practitioner views such as on targeting, uptake and adherence (e.g., barriers and enablers/facilitators, willingness to pay)?  🞏 Question(s) about intervention effects on intermediate outcomes?  🞏 Question(s) about intervention effects on final/endpoint outcomes?  🞏 Other question(s) (specify)? | 🞏 Yes  🞏 Partially  🞏 No  *Coding guide:*  *YES: For any aspects of the causal chain answered using systematic evidence, the review should have clear questions.*  *NO: Clear questions are not asked.*  *PARTIALLY: Some questions are specified but not for all parts of the causal chain on which the study claims to report systematic evidence.* |
| *Comments (note important limitations or uncertainty)* | |
| D.3 Were study design criteria reported for review sub-components (primary and secondary questions)?  Did the authors specify study design criteria for answering different questions along causal chain:  🞏 Criteria for types of studies for answering questions about intervention design and/or implementation processes, such as inputs, activities and outputs (e.g., process evaluation)?  🞏 Criteria for types of studies for answering questions about participant or practitioner views such as on targeting, uptake and adherence (e.g., ethnographic studies, rapid appraisal methods e.g., participatory rural appraisal)?  🞏 Criteria for types of studies for answering questions of intervention effects on intermediate and final/endpoint outcomes (e.g., RCTs, non-randomised counterfactual-based studies)?  🞏 Criteria for types of studies for answering other questions (specify)? | 🞏 Yes  🞏 Partially  🞏 No  🞏 Not applicable  *Coding guide:*  *YES: For any aspects of the causal chain answered using systematic evidence, the review should have clear study design criteria.*  *NO: No boxes are ticked.*  *PARTIALLY: Study types for some review questions are specified but not others.*  *NOT APPLICABLE: No secondary review component.* |
| *Comments (note important limitations or uncertainty)* | |
| D.4 Was the qualitative evidence included?  Did the authors:  🞏 Search systematically for all qualitative evidence to answer the review question?  🞏 Search systematically for qualitative evidence relevant to the particular contexts in which evidence of effects is available?  🞏 Conduct targeted searches for specific types of evidence (e.g., project and implementation documents)?  🞏 Only include qualitative evidence contained in included quantitative study reports? | 🞏 Yes  🞏 Partially  🞏 No  🞏 Not applicable (e.g., no studies or no data)  *Coding guide:*  *YES: Systematic searches for qualitative evidence were made.*  *NO: Only evidence from quantitative reports included.*  *PARTIALLY: Systematic or targeted searches for qualitative evidence relevant to the contexts in which evidence of effects is available.*  *NOT APPLICABLE: No studies or data available.* |
| *Comments (note important limitations or uncertainty)* | |
| D.5 Did the review conduct analysis of along the causal pathway?  🞏 The review conducted analysis of primary endpoint outcomes.  🞏 The review conducted analysis of primary intermediate outcomes.  🞏 The review conducted analysis of secondary endpoint outcomes.  🞏 The review conducted analysis of secondary intermediate outcomes.  Note: Primary outcomes are outcomes that must be reported regardless of any other outcome. Any relevant study including a primary outcome is eligible for inclusion in the review. Secondary outcomes are outcomes that are only reported if primary outcomes are also reported. Any relevant study including a secondary outcome but not a primary outcome is ineligible for inclusion. | 🞏 Yes  🞏 Partially  🞏 No  🞏 Not applicable (e.g., no studies or no data)  *Coding guide:*  *YES: Boxes 1 and 2 are ticked*  *PARTIALLY: Boxes 1 and 4 or 2 and 3 are ticked.*  *NO: analysis of outcomes along causal chain is not undertaken and only endpoint outcomes are analysed (and outcomes at different stages of the causal chain were excluded).*  *NOT APPLICABLE: if no studies/no data.* |
| *Comments (note important limitations or uncertainty)* | |
| D.6 Did the review incorporate qualitative evidence in the analysis?  🞏 To answer specific review questions about intervention design (e.g., project portfolio information)?  🞏 To answer specific review questions about barriers and facilitators/enablers of implementation (assumptions or risks in the causal chain/ logic model)?  🞏 To answer specific review questions about adherence or participant views?  🞏 To provide evidence on ‘middle-range’ causal mechanisms or contextual factors (e.g., identification of moderators, policy context, general equilibrium effects, sustained adherence)?  🞏 To discuss the quantitative findings in light of qualitative evidence (i.e., in the discussion section only), not drawing on evidence collected systematically? | 🞏 Yes  🞏 No  🞏 Partially  🞏 Not applicable  *Coding guide:*  *YES: Boxes 1, 2, 3, or 4 are reported.*  *NO: None are reported.*  *PARTIALLY: Box 5 is reported only.* |
| *Comments (note important limitations or uncertainty)* | |
| D.7 Did the review integrate the findings from quantitative and qualitative evidence?  Is qualitative evidence:  🞏 Presented in a separate results section?  🞏 Integrated with evidence on effects using qualitative methods (e.g., further iterations of the theory of change)  🞏 Integrated with evidence on effects using quantitative methods (e.g., through formal statistical testing of moderators and sub-groups identified in qualitative analysis)  🞏 Some other method to integrate the evidence (specify)? | 🞏 Yes  🞏 No  🞏 Partially  🞏 Not applicable  *Coding guide:*  *YES: Boxes 1 and 2 or 3 are reported.*  *NO: None are reported.*  *PARTIALLY: 1 is reported only.*  *NOT APPLICABLE: if no studies/no data.* |
| *Comments (note important limitations or uncertainty)* | |
| D.8 Were methods used to form conclusions and implications:  🞏 Weight of quantitative evidence assessed using GRADE (Schünemann et al., 2008) or other methods?  🞏 Weight of qualitative evidence assessed using cerQUAL (Lewin et al., 2015) or other methods?  🞏 Some method is used to integrate findings from quantitative and qualitative approaches (e.g., summary of findings table) to determine conclusions and implications? | 🞏 Yes  🞏 No  🞏 Partially  🞏 Not applicable  *Coding guide:*  *YES: All are reported (or for reviews of quantitative evidence only, box 1 is reported).*  *NO: None are reported.*  *PARTIALLY: Boxes 1, 2 or 3 are reported.*  *NOT APPLICABLE: if no studies/no data.* |
| *Comments (note important limitations or uncertainty)* | |
| D.9 Overall – categorise the approach to incorporating theory and qualitative evidence in the review of effects (tick all that apply):  🞏 Use of explicit theory or logic model?  🞏 Collection and presentation of outcomes along causal pathway?  🞏 Incorporation of unsystematic qualitative evidence?  🞏 Incorporation of systematic qualitative evidence?  🞏 Systematic integration of quantitative and qualitative evidence? | 🞏 Traditional systematic review drawing solely on quantitative evidence of effects without explicit model of behaviour  🞏 Systematic review using explicit model of behaviour change and drawing exclusively on quantitative evidence for endpoint outcome(s)  🞏 Theory-based systematic review drawing exclusively on quantitative evidence along causal pathway (intermediate and endpoint outcome(s))  🞏 Theory-based systematic review drawing systematically on quantitative evidence and unsystematically on qualitative evidence along causal pathway  🞏 Theory-based systematic review drawing systematically on quantitative and qualitative evidence along causal pathway  🞏 Integrated, theory-based systematic review drawing systematically on quantitative and qualitative evidence along causal pathway  🞏 Other (specify) |
| *Use comments to specify if relevant, to flag uncertainty or need for discussion* | |

# Appendix D: Systematic review critical appraisals

**Table D1: Reviews of combined WASH on diarrhoea**

|  | Review name | Esrey et al. 1991 | Fewtrell and Colford 2004 | Waddington et al. 2009 | Cairncross et al. 2010 | Darvesh et al. 2017 | Wolf et al. 2018 |
| --- | --- | --- | --- | --- | --- | --- | --- |
|  | Final search date | 1989 | Jun-03 | Feb-09 | 2005 and 2008 | Sep-16 | Feb-16 |
| A.1 | Protocol | No | No | Partially | Partially | No | Partially |
| A.2 | PICOS specification | No | Partially | Yes | Yes | Yes | Yes |
| A.3 | Search comprehensiveness | No | Partially | Yes | Partially | No | No |
| A.4 | Time period | Yes | Yes | Yes | Partially | Yes | Yes |
| A.5 | Study inclusion decisions | No | No | Yes | Yes | Yes | No |
| A.6 | Dependency | Yes | Yes | Partially | Can't tell | No | Yes |
| A. Overall | Overall search and critical appraisal | Low confidence | Low confidence | Medium confidence | Medium confidence | Low confidence | Low confidence |
| B.1 | Reporting studies | No | No | Partially | Yes | Partially | Partially |
| B.2 | Risk of bias | Unclear | Yes | Partially | Yes | Partially | Partially |
| B.3 | Clarity in analysis including effect sizes | No | Yes | Yes | Yes | Yes | Yes |
| B.4 | Describing heterogeneity | No | Yes | Yes | Yes | Partially | Yes |
| B.5 | Synthesis including meta-analysis | No | Partially | Partially | Partially | Partially | Partially |
| B.6 | Reporting by RoB | Partially | Partially | Yes | Yes | Partially | Yes |
| B.7 | Exploring heterogeneity including moderator analysis | No | Yes | Yes | Yes | Partially | Yes |
| B. Overall | Overall quantitative analysis | Low confidence | Low confidence | Medium confidence | Medium confidence | Medium confidence | Medium confidence |
| C.3 | Confidence rating | Low confidence | Low confidence | Medium confidence | Medium confidence | Low confidence | Low confidence |
| D.1 | Theory | No | Partially | Yes | No | No | Partially |
| D.2 | Review questions | NA | NA | Yes | NA | NA | NA |
| D.3 | PICOS by review question | NA | NA | Yes | NA | NA | NA |
| D.4 | Qualitative evidence | No | No | Partially | No | No | No |
| D.5 | Quantitative causal chain analysis | No | No | Partially | No | No | No |
| D.6 | Qualitative causal chain analysis | NA | NA | Partially | NA | NA | NA |
| D.7 | Integration of results | NA | NA | Partially | NA | NA | NA |
| D.8 | Conclusions | No | No | No | No | Yes | No |

**Table D2: Reviews of hygiene, water or sanitation on diarrhoea**

|  |  | *Hygiene* | | | | *Water* | | | | *Sanitation* | | |
| --- | --- | --- | --- | --- | --- | --- | --- | --- | --- | --- | --- | --- |
|  | Review name | Curtis and Cairncross 2003 | Aiello et al. 2008 | Freeman et al. 2014 | Ejemot-Nwadiaro et al. 2015 | Gundry et al. 2004 | Arnold and Colford 2007 | Hunter 2009 | Clasen 2015 | Clasen et al. 2010 | Norman et al. 2010 | Heijnen 2014 |
|  | Final search date | 2002 | May-07 | Aug-13 | May-15 | Dec-00 | Mar-06 | Feb-09 | 11-Nov-14 | Unclear | Feb-10 | 01-Sep-13 |
| A.1 | Protocol | No | No | Partially | Yes | No | No | No | Yes | Yes | No | Partially |
| A.2 | PICOS specification | Partially | Yes | Yes | Yes | Yes | Yes | Yes | Yes | Yes | Yes | Yes |
| A.3 | Search comprehensiveness | Partially | No | No | Yes | Partially | Partially | Partially | Yes | Yes | No | Yes |
| A.4 | Time period | Unsure | Yes | Yes | Yes | Can’t tell | Can't tell | Yes | Yes | Yes | Yes | Yes |
| A.5 | Study inclusion decisions | No | Partially | No | Yes | No | Partially | Yes | Yes | Yes | Partially | Yes |
| A.6 | Dependency | Partially | No | No | Yes | Yes | Yes | Partially | No | Yes | Partially | Yes |
| A. Overall | Overall search and critical appraisal | Low confidence | Low confidence | Low confidence | High confidence | Medium confidence | Medium confidence | Medium confidence | Medium confidence | High confidence | Low confidence | High confidence |
| B.1 | Reporting studies | Partially | Yes | No | Yes | Partially | Yes | Yes | Yes | Yes | No | Yes |
| B.2 | Risk of bias | Yes | No | No | Yes | No | Partially | Partially | Yes | Yes | Partially | Yes |
| B.3 | Clarity in analysis including effect sizes | Yes | Yes | No | Yes | Yes | Yes | Yes | Yes | Yes | Yes | Partially |
| B.4 | Describing heterogeneity | Yes | Yes | No | Yes | No | Yes | Yes | Yes | Yes | No | Yes |
| B.5 | Synthesis including meta-analysis | Partially | Yes | No | Yes | Partially | Partially | Partially | Partially | Yes | Partially | Partially |
| B.6 | Reporting by RoB | Yes | No | No | Yes | Partially | Partially | Partially | Yes | Yes | Partially | Partially |
| B.7 | Exploring heterogeneity including moderator analysis | Yes | Yes | No | Yes | Yes | Yes | Yes | Yes | Yes | Yes | No |
| B. Overall | Overall quantitative analysis | Medium confidence | Medium confidence | Low confidence | High confidence | Medium confidence | Medium confidence | Medium confidence | Medium confidence | High confidence | Low confidence | Medium confidence |
| C.3 | Confidence rating | Medium confidence | Low confidence | Low confidence | High confidence | Medium confidence | Medium confidence | Medium confidence | Medium confidence | | Low confidence | Medium confidence |
| D.1 | Theory | No | No | No | No | No | No | No | No | No | No | No |
| D.2 | Review questions | NA | NA | NA | Yes | Yes | Yes | No | NA | NA | NA | NA |
| D.3 | PICOS by review question | NA | NA | NA | Yes | Yes | Yes | NA | NA | NA | NA | NA |
| D.4 | Qualitative evidence | No | No | No | No | No | No | No | No | No | No | No |
| D.5 | Quantitative causal chain analysis | No | No | No | Partially | Partially | Yes | Partially | No | No | No | No |
| D.6 | Qualitative causal chain analysis | NA | NA | NA | NA | NA | NA | NA | No | No | NA | NA |
| D.7 | Integration of results | NA | NA | NA | NA | NA | NA | NA | NA | NA | NA | NA |
| D.8 | Conclusions | No | No | No | Yes | No | No | No | Yes | Yes | No | No |

**Table D3: Reviews of WASH on helminth infections, trachoma and nutrition**

|  |  | *Helminth infections* | | | | *Trachoma* | | | *Nutrition* |
| --- | --- | --- | --- | --- | --- | --- | --- | --- | --- |
|  | Review name | Freeman et al. 2017 | Ziegelbauer et al. 2012 | Strunz et al. 2014 | Garn et al. 2017 | Rabiu et al. 2012 | Stocks et al. 2014 | Ejere et al. 2015 | Dangour et al. 2013 |
|  | Final search date | Dec-15 | Dec-10 | Oct-13 | Dec-15 | Sep-11 | 27-Oct-13 | Jan-15 | Jun-12 |
| A.1 | Protocol | Partially | Partially | Partially | Partially | Yes | Partially | Yes | Yes |
| A.2 | PICOS specification | Yes | Partially | Partially | Yes | Yes | Yes | Yes | Yes |
| A.3 | Search comprehensiveness | No | Partially | No | Yes | Partially | No | Yes | Yes |
| A.4 | Time period | Yes | Can't tell | Yes | Yes | Yes | Yes | Yes | Yes |
| A.5 | Study inclusion decisions | Yes | Yes | Yes | Yes | Yes | Yes | Yes | Yes |
| A.6 | Dependency | No | No | Yes | No | Partially | No | Partially | Yes |
| A. Overall | Overall search and critical appraisal | Low confidence | Low confidence | Low confidence | Medium confidence | High confidence | Low confidence | High confidence | High confidence |
| B.1 | Reporting studies | Yes | Yes | Yes | Partially | Yes | No | Yes | Yes |
| B.2 | Risk of bias | Partially | No | Yes | Partially | Yes | No | Yes | Yes |
| B.3 | Clarity in analysis including effect sizes | Yes | Yes | Yes | Yes | Yes | Yes | Yes | Yes |
| B.4 | Describing heterogeneity | Yes | Yes | Yes | Yes | Yes | Yes | Yes | Yes |
| B.5 | Synthesis including meta-analysis | Partially | Partially | Partially | Partially | Yes | Partially | Yes | Yes |
| B.6 | Reporting by RoB | No | No | Partially | Partially | Yes | No | Yes | Partially |
| B.7 | Exploring heterogeneity including moderator analysis | Yes | Partially | Yes | Yes | Yes | Partially | Yes | Partially |
| B. Overall | Overall quantitative analysis | Low confidence | Medium confidence | Medium confidence | Medium confidence | High confidence | Low confidence | High confidence | Medium confidence |
| C.3 | Confidence rating | Low confidence | Low confidence | Low confidence | Medium confidence | High confidence | Low confidence | High confidence | High confidence |
| D.1 | Theory | No | No | No | No | No | No | No | Partially |
| D.2 | Review questions | NA | NA | NA | NA | Yes | NA | NA | NA |
| D.3 | PICOS by review question | NA | NA | NA | NA | Yes | NA | NA | NA |
| D.4 | Qualitative evidence | No | No | No | No | No | No | No | No |
| D.5 | Quantitative causal chain analysis | Yes | Partially | No | No | Partially | No | No | No |
| D.6 | Qualitative causal chain analysis | NA | NA | NA | NA | NA | NA | NA | NA |
| D.7 | Integration of results | NA | NA | NA | NA | NA | NA | NA | NA |
| D.8 | Conclusions | Yes | No | Yes | Yes | No | No | No | Yes |

**Table D4: Reviews of WASH behaviour outcomes**

|  | Review name | Lucas et al. 2011 | Null et al. 2012 | Fiebelkorn et al. 2012 | Evans et al. 2014 | Annamalai et al. 2016 | Morita et al. 2016 | De Buck et al. 2017 | Watson et al. 2017 | Venkataraman et al. 2018 | Majorin et al. 2019 |
| --- | --- | --- | --- | --- | --- | --- | --- | --- | --- | --- | --- |
|  | Final search date | 2010 | Unclear | Jul-10 | Jun-13 | Dec-13 | Mar-16 | Mar-16 | Jul-16 | Mar-17 | Sep-18 |
| A.1 | Protocol | Partially | No | No | No | Partially | No | Yes | No | No | Yes |
| A.2 | PICOS specification | Yes | Yes | Yes | Partially | Yes | Partially | Yes | Yes | Yes | Yes |
| A.3 | Search comprehensiveness | Partially | Partially | No | No | Partially | No | Partial | No | Partially | Yes |
| A.4 | Time period | Can't tell | Unclear | Yes | Yes | Unclear | Yes | Yes | Yes | Yes | Yes |
| A.5 | Study inclusion decisions | Yes | Partially | Yes | Yes | Partially | No | Yes | Yes | Partially | Yes |
| A.6 | Dependency | Yes | Yes | Unclear | Partially | Unclear | Yes | Yes | Yes | Partially | Yes |
| A. Overall | Overall search and critical appraisal | Medium confidence | Medium confidence | Low confidence | Low confidence | Medium confidence | Low confidence | Low confidence | Low confidence | Low confidence | High confidence |
| B.1 | Reporting studies | Yes | No | No | Partially | Partially | No | Yes | No | Partially | Yes |
| B.2 | Risk of bias | Yes | No | No | No | Partially | Partially | Yes | Yes | No | Yes |
| B.3 | Clarity in analysis including effect sizes | Yes | Yes | No | No | Yes | Yes | Yes | Yes | Partially | Yes |
| B.4 | Describing heterogeneity | Yes | Yes | No | Partially | Partially | Yes | Yes | Yes | Partially | Yes |
| B.5 | Synthesis including meta-analysis | Partially | No | No | No | Partially | Partially | Yes | No | No | Yes |
| B.6 | Reporting by RoB | Yes | Partially | No | No | Partially | No | Yes | Yes | No | Partially |
| B.7 | Exploring heterogeneity including moderator analysis | Yes | Partially | Partially | Yes | Yes | No | Yes | Yes | Partially | Yes |
| B. Overall | Overall quantitative analysis | High confidence | Low confidence | Low confidence | Low confidence | Medium confidence | Low confidence | High confidence | Low confidence | Low confidence | Medium confidence |
| C.3 | Confidence | Medium confidence | Low confidence | Low confidence | Low confidence | Medium confidence | Low confidence | Medium confidence | Low confidence | Medium confidence | Medium confidence |
| D.1 | Theory | No | No | No | No | Partially | No | Yes | No | No | Partially |
| D.2 | Review questions | Partially | NA | NA | Partially | Yes | Partially | Yes | NA | No | NA |
| D.3 | PICOS by review question | Yes | NA | NA | No | Yes | Yes | Yes | NA | Partially | NA |
| D.4 | Qualitative evidence | Partially | No | Partially | No | Yes | No | Yes | No | Yes | No |
| D.5 | Quantitative causal chain analysis | Yes | No | No | Partially | No | Partially | Partially | Yes | Partially | Partially |
| D.6 | Qualitative causal chain analysis | No | NA | No | NA | Yes | NA | Yes | NA | Yes | NA |
| D.7 | Integration of results | No | NA | No | NA | Partially | NA | Partially | NA | Partially | NA |
| D.8 | Conclusions | No | No | No | No | No | No | Yes | No | No | Yes |
